# Bose–Einstein Condensation for Two Dimensional Bosons in the Gross–Pitaevskii Regime

**DOI:** 10.1007/s10955-021-02766-6

**Published:** 2021-05-20

**Authors:** Cristina Caraci, Serena Cenatiempo, Benjamin Schlein

**Affiliations:** 1 University of Zurich, Winterthurerstr. 190, 8057 Zurich, Switzerland; 2 Gran Sasso Science Institute, Viale F. Crispi 7, 67100 L’Aquila, Italy

**Keywords:** Two dimensional Bose gas, Bose–Einstein condensation, Gross-Pitaevskii regime, Interacting bosons, 46N50, 81V70, 81V73, 82B10, 82B27, 82D03

## Abstract

We consider systems of N bosons trapped on the two-dimensional unit torus, in the Gross-Pitaevskii regime, where the scattering length of the repulsive interaction is exponentially small in the number of particles. We show that low-energy states exhibit complete Bose–Einstein condensation, with almost optimal bounds on the number of orthogonal excitations.

## Introduction

We consider $N \in {\mathbb {N}}$ bosons trapped in the two-dimensional box $\varLambda = [-1/2;1/2]^2$ with periodic boundary conditions. In the Gross-Pitaevskii regime, particles interact through a repulsive pair potential, with a scattering length exponentially small in *N*. The Hamilton operator is given by

1 $$\begin{aligned} H_N = \sum _{j=1}^N -\varDelta _{x_j} + \sum _{i<j}^N e^{2N} V (e^N(x_i -x_j)) \end{aligned}$$

and acts on a dense subspace of $L^2_s (\varLambda ^N)$, the Hilbert space consisting of functions in $L^2 (\varLambda ^N)$ that are invariant with respect to permutations of the *N* particles. We assume here $V \in L^3 ({\mathbb {R}}^2)$ to be compactly supported and pointwise non-negative (i.e. $V(x) \ge 0$ for almost all $x \in {\mathbb {R}}^2$).

We denote by ${{\mathfrak {a}}}$ the scattering length of the unscaled potential *V*. We recall that in two dimensions and for a potential *V* with finite range $R_0$, the scattering length is defined by

2 $$\begin{aligned} \frac{2\pi }{\log (R/{{\mathfrak {a}}})} =\inf _{\phi } \int _{B_R} \left[ |\nabla \phi |^2 + \frac{1}{2} V |\phi |^2 \right] dx \end{aligned}$$

where $R > R_0$, $B_R$ is the disk of radius *R* centered at the origin and the infimum is taken over functions $\phi \in H^1(B_R)$ with $\phi (x)=1$ for all *x* with $|x|=R$. The unique minimizer of the variational problem on the r.h.s. of (2) is non-negative, radially symmetric and satisfies the scattering equation

$$\begin{aligned} - \varDelta \phi ^{(R)} + \frac{1}{2} V \phi ^{(R)} =0 \end{aligned}$$

in the sense of distributions. For $R_0 < |x| \le R$, we have

$$\begin{aligned} \phi ^{(R)} (x) = \frac{\log (|x|/{\mathfrak {a}})}{\log (R/{{\mathfrak {a}}}) }\,. \end{aligned}$$

By scaling, $\phi _N (x) := \phi ^{(e^N R)} (e^N x)$ is such that

$$\begin{aligned} -\varDelta \phi _N + \frac{1}{2} e^{2N} V (e^N x) \phi _N = 0 \end{aligned}$$

We have

$$\begin{aligned} \phi _{N}(x) =\frac{\log (|x|/{{\mathfrak {a}}}_N)}{\log (R/{{\mathfrak {a}}}_N) } \qquad \forall x \in {\mathbb {R}}^2 : e^{-N} R_0 < |x| \le R\,, \end{aligned}$$

for all $x \in {\mathbb {R}}^2$ with $e^{-N} R_0 < |x| \le R$. Here ${{\mathfrak {a}}}_N= e^{-N} {{\mathfrak {a}}}$.

The spectral properties of trapped two dimensional bosons in the Gross-Pitaevskii regime (in the more general case where the bosons are confined by external trapping potentials) have been first studied in [13, 14, 16]. These results can be translated to the Hamilton operator (1), defined on the torus, with no external potential. They imply that the ground state energy $E_N$ of (1) is such that

3 $$\begin{aligned} E_N = 2 \pi N \big ( 1 + O(N^{-1/5})\big )\,. \end{aligned}$$

Moreover, they imply Bose–Einstein condensation in the zero-momentum mode $\varphi _0 (x) = 1$ for all $x \in \varLambda$, for any approximate ground state of (1). More precisely, it follows from [13] that, for any sequence $\psi _N \in L^2_s (\varLambda ^N)$ with $\Vert \psi _N \Vert = 1$ and

4 $$\begin{aligned} \lim _{N \rightarrow \infty } \frac{1}{N} \langle \psi _N , H_N \psi _N \rangle = 2\pi , \end{aligned}$$

the one-particle reduced density matrix $\gamma _N = \mathrm{tr}_{2, \ldots , N} |\psi _N \rangle \langle \psi _N |$ is such that

5 $$\begin{aligned} 1 - \langle \varphi _0 , \gamma _N \varphi _0 \rangle \le CN^{-{\bar{\delta }}} \end{aligned}$$

for a sufficiently small ${\bar{\delta }}>0$. The estimate (5) states that, in many-body states satisfying (4) (approximate ground states), almost all particles are described by the one-particle orbital $\varphi _0$, with at most $N^{1-\delta } \ll N$ orthogonal excitations.

Similar results have been obtained starting from a three dimensional Bose gas, trapped by a potential which is strongly confining in one direction, so that the system becomes effectively two-dimensional [22]. Finally, let us also mention [5, 10], where rigorous results on the time-evolution in the two-dimensional Gross-Pitaevskii regime have been established (in [5], the focus is on the dynamics of a three-dimensional gas, with strong confinement in one direction).

For $V \in L^3({\mathbb {R}}^2)$, our main theorem improves (3) and (5) by providing more precise bounds on the ground state energy and on the number of excitations.

### Theorem 1

Let $V \in L^3 ({\mathbb {R}}^2)$ have compact support, be spherically symmetric and pointwise non-negative. Then there exists a constant $C > 0$ such that the ground state energy $E_N$ of (1) satisfies

6 $$\begin{aligned} 2\pi N - C \le E_N \le 2\pi N + C \log N\,. \end{aligned}$$

Furthermore, consider a sequence $\psi _N \in L^2_s (\varLambda ^N)$ with $\Vert \psi _N \Vert = 1$ and such that

7 $$\begin{aligned} \langle \psi _N , H_N \psi _N \rangle \le 2\pi N + K \end{aligned}$$

for a $K > 0$. Then the reduced density matrix $\gamma _N = \mathrm{tr}_{2,\ldots , N} | \psi _N \rangle \langle \psi _N |$ associated with $\psi _N$ is such that

8 $$\begin{aligned} 1 - \langle \varphi _0 , \gamma _N \varphi _0 \rangle \le \frac{C(1+ K)}{N} \end{aligned}$$

for all $N \in {\mathbb {N}}$ large enough.

### Remark

We expect that the bounds of Theorem 1 can be extended to two-dimensional systems of bosons trapped by an external potential (in three dimensions, similar estimates have been recently established in [7, 19]). In this case, the system exhibits condensation in the minimizer of the Gross-Pitaevskii energy functional, as shown in [13, 14, 16].

It is interesting to compare the Gross-Pitaevskii regime with the thermodynamic limit, where a Bose gas of *N* particles interacting through a fixed potential with scattering length ${{\mathfrak {a}}}$ is confined in a box with area $L^2$, so that $N, L \rightarrow \infty$ with the density $\rho =N/L^2$ kept fixed. Let $b=|\log (\rho {{\mathfrak {a}}}^2)|^{-1}$. Then, in the dilute limit $\rho {{\mathfrak {a}}}^2 \ll 1$, the ground state energy per particle in the thermodynamic limit is expected to satisfy

9 $$\begin{aligned} e_0(\rho ) = 4 \pi \rho ^2 b \Big ( 1 + b \log b + \big (1/2 + 2\gamma + \log \pi \big ) b + o(b) \Big )\,, \end{aligned}$$

with $\gamma$ the Euler’s constant. The leading order term on the r.h.s. of (9) has been first derived in [21] and then rigorously established in [15], with an error rate $b^{-1/5}$. The corrections up to order *b* have been predicted in [1, 18, 20]. To date, there is no rigorous proof of (9). Some partial result, based on the restriction to quasi-free states, has been recently obtained in [9, Theorem 1].

Extrapolating from (9), in the Gross-Pitaevskii regime we expect $|E_N - 2\pi N| \le C$. While our estimate (6) captures the correct lower bound, the upper bound is off by a logarithmic correction. Eq. (8), on the other hand, is expected to be optimal (but of course, by (6), we need to choose $K = C \log N$ to be sure that (7) can be satisfied). This bound can be used as starting point to investigate the validity of Bogoliubov theory for two dimensional bosons in the Gross-Pitaevskii regime, following the strategy developed in [3] for the three dimensional case; we plan to proceed in this direction in a separate paper.

The proof of Theorem 1 follows the strategy that has been recently introduced in [4] to prove condensation for three-dimensional bosons in the Gross-Pitaevskii limit. There are, however, additional obstacles in the two-dimensional case, requiring new ideas. To appreciate the difference between the Gross-Pitaevskii regime in two- and three-dimensions, we can compute the energy of the trivial wave function $\psi _N \equiv 1$. The expectation of (1) in this state is of order $N^2$. It is only through correlations that the energy can approach (6). Also in three dimensions, uncorrelated many-body wave functions have large energy, but in that case the difference with respect to the ground state energy is only of order *N* ($N \widehat{V} (0)/2$ rather than $4\pi \mathfrak {a} N$). This observation is a sign that correlations in two-dimensions are stronger and play a more important role than in three dimensions (this creates problems in handling error terms that, in the three dimensional setting, were simply estimated in terms of the integral of the potential).

The paper is organized as follows. In Sect. 2 we introduce our setting, based on a description of orthogonal excitations of the condensate on a truncated Fock space. Factoring out the condensate, we introduce an excitation Hamiltonian $\mathcal{L}_N$, unitarily equivalent to $H_N$. In Sects. 3 and 4 we define two additional unitary maps, modelling the correlation structure characterising low-energy states. The first map is a generalized Bogoliubov transformation, given by the exponential of an anti-symmetric operator *B*, quadratic in creation and annihilation operators, see Eq. (33). Its action on $\mathcal{L}_N$ leads to a second excitation Hamiltonian $\mathcal{G}_{N,\alpha }$, whose vacuum expectation matches (6), at leading order. Unfortunately, $\mathcal{G}_{N,\alpha }$ is not coercive enough to directly show Bose–Einstein condensation. To overcome this difficulty, we conjugate the main part of $\mathcal{G}_{N,\alpha }$ (later denoted by $\mathcal{G}_{N,\alpha }^{\text {eff}}$) with a second unitary map, given by the exponential of an operator *A*, cubic in creation and annihilation operators, see Eq. (44). This defines a renormalized excitation Hamiltonian $\mathcal{R}_{N,\alpha }$, where the singular interaction is regularized. In Sect. 5 we combine the bounds on $\mathcal{G}_{N,\alpha }$ and $\mathcal{R}_{N,\alpha }$ with a localization argument proposed in [11] for the number of excitations to conclude the proof of Theorem 1. Section 6 and App. 1 are devoted to the proof of the bounds on $\mathcal{G}_{N,\alpha }$ and on $\mathcal{R}_{N,\alpha }$ stated in Sects. 3 and 4, respectively. Finally, in App. 1, we establish some properties of the solution of the Neumann problem associated with the two-body potential *V*.

## The Excitation Hamiltonian

Low-energy states of (1) exhibit condensation in the zero-momentum mode $\varphi _0$ defined by $\varphi _0 (x) = 1$ for all $x \in \varLambda = [-1/2;1/2]^2$. Similarly as in [2, 4, 11], we are going to describe excitations of the condensate on the truncated bosonic Fock space

$$\begin{aligned} \mathcal{F}^{\le N}_+ = \bigoplus _{k=0}^N L^2_\perp (\varLambda )^{\otimes _s k} \end{aligned}$$

constructed on the orthogonal complement $L^2_\perp (\varLambda )$ of $\varphi _0$ in $L^2 (\varLambda )$. To reach this goal, we define a unitary map $U_N : L^2_s (\varLambda ^N) \rightarrow \mathcal{F}_+^{\le N}$ by requiring that $U_N \psi _N = \{ \alpha _0, \alpha _1, \ldots , \alpha _N \}$, with $\alpha _j \in L^2_\perp (\varLambda )^{\otimes _s j}$, if

$$\begin{aligned} \psi _N= \alpha _0 \varphi _0^{\otimes N} + \alpha _1 \otimes _s \varphi _0^{\otimes (N-1)} + \cdots + \alpha _N \end{aligned}$$

With the usual creation and annihilation operators, we can write

$$\begin{aligned} U_N \, \psi _N = \bigoplus _{n=0}^N (1-|\varphi _0 \rangle \langle \varphi _0|)^{\otimes n} \frac{a (\varphi _0)^{N-n}}{\sqrt{(N-n)!}} \, \psi _N \end{aligned}$$

for all $\psi _N \in L^2_s (\varLambda ^N)$. It is then easy to check that $U_N^* : \mathcal{F}_{+}^{\le N} \rightarrow L^2_s (\varLambda ^N)$ is given by

$$\begin{aligned} U_N^* \, \{ \alpha ^{(0)}, \ldots , \alpha ^{(N)} \} = \sum _{n=0}^N \frac{a^* (\varphi _0)^{N-n}}{\sqrt{(N-n)!}} \, \alpha ^{(n)} \end{aligned}$$

and that $U_N^* U_N = 1$, i.e. $U_N$ is unitary.

With $U_N$, we can define the excitation Hamiltonian $\mathcal{L}_N := U_N H_N U_N^*$, acting on a dense subspace of $\mathcal{F}_+^{\le N}$. To compute the operator $\mathcal{L}_N$, we first write the Hamiltonian (1) in momentum space, in terms of creation and annihilation operators $a_p^*, a_p$, for momenta $p \in \varLambda ^* = 2\pi {\mathbb {Z}}^2$. We find

10 $$\begin{aligned} H_N = \sum _{p \in \varLambda ^*} p^2 a_p^* a_p + \frac{1}{2}\sum _{p,q,r \in \varLambda ^*} \widehat{V} (r/e^N) a_{p+r}^* a_q^* a_{p} a_{q+r} \end{aligned}$$

where

$$\begin{aligned} \widehat{V} (k) = \int _{{\mathbb {R}}^2} V (x) e^{-i k \cdot x} dx \end{aligned}$$

is the Fourier transform of *V*, defined for all $k \in {\mathbb {R}}^2$ (in fact, (1) is the restriction of (10) to the *N*-particle sector of the Fock space). We can now determine $\mathcal{L}_N$ using the following rules, describing the action of the unitary operator $U_N$ on products of a creation and an annihilation operator (products of the form $a_p^* a_q$ can be thought of as operators mapping $L^2_s (\varLambda ^N)$ to itself). For any $p,q \in \varLambda ^*_+ = 2\pi {\mathbb {Z}}^2 \backslash \{ 0 \}$, we find (see [11]):

11 $$\begin{aligned} \begin{aligned} U_N \, a^*_0 a_0 \, U_N^*&= N- \mathcal{N}_+ \\ U_N \, a^*_p a_0 \, U_N^*&= a^*_p \sqrt{N-\mathcal{N}_+ } \\ U_N \, a^*_0 a_p \, U_N^*&= \sqrt{N-\mathcal{N}_+ } \, a_p \\ U_N \, a^*_p a_q \, U_N^*&= a^*_p a_q \,. \end{aligned} \end{aligned}$$

where $\mathcal{N}_+ = \sum _{p\in \varLambda ^*_+} a_p^* a_p$ is the number of particles operator on $\mathcal{F}_+^{\le N}$. We conclude that

12 $$\begin{aligned} \mathcal{L}_N = \mathcal{L}^{(0)}_{N} + \mathcal{L}^{(2)}_{N} + \mathcal{L}^{(3)}_{N} + \mathcal{L}^{(4)}_{N} \end{aligned}$$

with

13 $$\begin{aligned} \begin{aligned} \mathcal{L}_{N}^{(0)} =\;&\frac{1}{2} \widehat{V} (0) (N-1)(N-\mathcal{N}_+ ) + \frac{1}{2} \widehat{V} (0) \mathcal{N}_+ (N-\mathcal{N}_+ ) \\ \mathcal{L}^{(2)}_{N} =\;&\sum _{p \in \varLambda ^*_+} p^2 a_p^* a_p + N\sum _{p \in \varLambda _+^*} \widehat{V} (p/e^N) \left[ b_p^* b_p - \frac{1}{N} a_p^* a_p \right] \\&+ \frac{N}{2} \sum _{p \in \varLambda ^*_+} \widehat{V} (p/e^N) \left[ b_p^* b_{-p}^* + b_p b_{-p} \right] \\ \mathcal{L}^{(3)}_{N} =\;&\sqrt{N} \sum _{p,q \in \varLambda _+^* : p+q \not = 0} \widehat{V} (p/e^N) \left[ b^*_{p+q} a^*_{-p} a_q + a_q^* a_{-p} b_{p+q} \right] \\ \mathcal{L}^{(4)}_{N} =\;&\frac{1}{2}\sum _{\begin{array}{c} p,q \in \varLambda _+^*, r \in \varLambda ^*: \\ r \not = -p,-q \end{array}} \widehat{V} (r/e^N) a^*_{p+r} a^*_q a_p a_{q+r} \,, \end{aligned} \end{aligned}$$

where we introduced generalized creation and annihilation operators

$$\begin{aligned} b^*_p = U_N a_p^* U_N^* = a^*_p \, \sqrt{\frac{N-\mathcal{N}_+}{N}} , \qquad \text {and } \quad b_p = U_N a_p U_N^* = \sqrt{\frac{N-\mathcal{N}_+}{N}} \, a_p \end{aligned}$$

for all $$p \in \varLambda ^*_+$$.

On states exhibiting complete Bose–Einstein condensation in the zero-momentum mode $\varphi _0$, we have $a_0 , a_0^* \simeq \sqrt{N}$ and we can therefore expect that $b_p^* \simeq a_p^*$ and that $b_p \simeq a_p$. From the canonical commutation relations for the standard creation and annihilation operators $a_p, a_p^*$, we find

14 $$\begin{aligned} \begin{aligned} {[} b_p, b_q^* ]&= \left( 1 - \frac{\mathcal{N}_+}{N} \right) \delta _{p,q} - \frac{1}{N} a_q^* a_p\\ {[} b_p, b_q ]&= [b_p^* , b_q^*] = 0 \,. \end{aligned} \end{aligned}$$

Furthermore,

$$\begin{aligned} {[} b_p, a_q^* a_r ] = \delta _{pq} b_r, \qquad [b_p^*, a_q^* a_r] = - \delta _{pr} b_q^* \end{aligned}$$

for all $p,q,r \in \varLambda _+^*$; this implies in particular that $[b_p , \mathcal{N}_+] = b_p$, $[b_p^*, \mathcal{N}_+] = - b_p^*$. It is also useful to notice that the operators $b^*_p, b_p$, like the standard creation and annihilation operators $a_p^*, a_p$, can be bounded by the square root of the number of particles operators; we find

$$\begin{aligned} \Vert b_p \xi \Vert \le \Vert \mathcal{N}_+^{1/2} \xi \Vert \,, \qquad \Vert b^*_p \xi \Vert \le \Vert (\mathcal{N}_+ + 1)^{1/2} \xi \Vert \end{aligned}$$

for all $\xi \in \mathcal{F}^{\le N}_+$. Since $\mathcal{N}_+ \le N$ on $\mathcal{F}_+^{\le N}$, the operators $b_p^* , b_p$ are bounded, with $\Vert b_p \Vert , \Vert b^*_p \Vert \le (N+1)^{1/2}$.

## Quadratic Renormalization

From (13) we see that conjugation with $U_N$ extracts, from the original quartic interaction in (10), some large constant and quadratic contributions, collected in $\mathcal{L}^{(0)}_N$ and $\mathcal{L}^{(2)}_N$ respectively. In particular, the expectation of $\mathcal{L}_N$ on the vacuum state $\varOmega$ is of order $N^2$, this being an indication of the fact that there are still large contributions to the energy hidden among cubic and quartic terms in $\mathcal{L}^{(3)}_N$ and $\mathcal{L}^{(4)}_N$. Since $U_N$ only removes products of the zero-energy mode $\varphi _0$, correlations among particles remain in the excitation vector $U_N \psi _N$. Indeed, correlations play a crucial role in the two dimensional Gross-Pitaevskii regime and carry an energy of order $N^2$.

To take into account the short scale correlation structure on top of the condensate, we consider the solution $f_{\ell }$ of the equation

15 $$\begin{aligned} \Big ( -\varDelta + \frac{1}{2} V(x) \Big ) f_{\ell }(x) = \lambda _{\ell }\, f_{\ell }(x) \end{aligned}$$

associated with the smallest possible eigenvalue $\lambda _\ell$, on the ball $|x| \le e^N \ell$, with Neumann boundary conditions and normalized so that $f_{\ell }(x) = 1$ for $|x|= e^N\ell$. Here and in the following we omit the *N*-dependence in the notation for $f_\ell$ and for $\lambda _\ell$. By scaling, we observe that $f_{\ell }(e^N\cdot )$ satisfies

$$\begin{aligned} \Big ( -\varDelta + \frac{e^{2N}}{2} V(e^N x) \Big ) f_{\ell }(e^Nx) = e^{2N}\lambda _{\ell }\, f_{\ell }(e^Nx) \end{aligned}$$

on the ball $|x| \le \ell$. We choose $\ell < 1/2$, so that the ball of radius $\ell$ is contained in the box $\varLambda = [-1/2 ; 1/2]^2$. We extend then $f_\ell (e^N.)$ to $\varLambda$, by setting $f_{N,\ell } (x) = f_\ell (e^Nx)$, if $|x| \le \ell$ and $f_{N,\ell } (x) = 1$ for $x \in \varLambda$, with $|x| > \ell$. Then, assuming also that $R_0 e^{-N} < \ell$ (later we will choose $\ell = N^{-\alpha }$, so this condition is satisfied, for all *N* large enough),

16 $$\begin{aligned} \Big ( -\varDelta + \frac{e^{2N}}{2} V(e^N x ) \Big ) f_{N,\ell }(x) = e^{2N}\lambda _{\ell }\, f_{N,\ell }(x)\chi _\ell (x)\,, \end{aligned}$$

where $\chi _\ell$ is the characteristic function of the ball of radius $\ell$. The Fourier coefficients of the function $f_{N,\ell }$ are given by

$$\begin{aligned} \widehat{f}_{N,\ell } (p) := \int _\varLambda f_\ell (e^Nx) e^{-i p \cdot x} dx \end{aligned}$$

for all $p \in \varLambda ^*$. We introduce also the function $w_\ell (x)=1-f_\ell (x)$ for $|x| \le e^N\ell$ and extend it by setting $w_\ell (x)=0$ for $|x| >e^N\ell$. Its re-scaled version is defined by $w_{N,\ell }: \varLambda \rightarrow {\mathbb {R}}$ $w_{N,\ell }(x)=w_\ell (e^Nx)$ if $|x| \le \ell$ and $w_{N,\ell }=0$ if $x \in \varLambda$ with $|x| > \ell$.

The Fourier coefficients of the re-scaled function $w_{N,\ell }$ are given by

17 $$\begin{aligned} {{\widehat{w}}}_{N,\ell }(p)=\int _\varLambda w_\ell (e^Nx)e^{-ip\cdot x}dx = e^{-2N}{{\widehat{w}}}_\ell \left( e^{-N}p\right) . \end{aligned}$$

We find $\widehat{f}_{N,\ell }(p) = \delta _{p,0}- e^{-2N}{{\widehat{w}}}_{\ell }(e^{-N}p)$. From the Neumann problem (16) we obtain

18 $$\begin{aligned} -p^2e^{-2N}{{\widehat{w}}}_{\ell }(e^{-N}p) +\frac{1}{2}\sum _{q \in \varLambda ^*}\widehat{V}(e^{-N}(p-q))\widehat{f}_{N,\ell }(q) =e^{2N} \lambda _\ell \sum _{q\in \varLambda ^*}{\widehat{\chi }}_\ell (p-q)\widehat{f}_{N,\ell }(q).\nonumber \\ \end{aligned}$$

where we used the notation ${\widehat{\chi }}_\ell$ for the Fourier coefficients of the characteristic function on the ball of radius $\ell$. Note that ${\widehat{\chi }}_\ell (p)= \ell ^2\, \widehat{\chi }(\ell p)$ with ${{\widehat{\chi }}}(p)$ the Fourier coefficients of the characteristic function on the ball of radius one.

In the next lemma, we collect some important properties of the solution of (15).

### Lemma 1

Let $V\in L^3({\mathbb {R}}^2)$ be non-negative, compactly supported (with range $R_0$) and spherically symmetric, and denote its scattering length by ${{\mathfrak {a}}}$. Fix $0<\ell <1/2$, *N* sufficiently large and let $f_{\ell }$ denote the solution of (16). Then

(i) $$\begin{aligned} 0 \le f_{\ell }(x) \le 1 \qquad \forall \, |x| \le e^N \ell \,. \end{aligned}$$
(ii) We have
  19 $$\begin{aligned} \left| \lambda _{\ell } - \frac{2}{(e^N\ell )^2 \log (e^N\ell /{{\mathfrak {a}}})} \right| \le \frac{C}{(e^N\ell )^2 \log ^2(e^N\ell /{{\mathfrak {a}}})} \end{aligned}$$
(iii) There exists a constant $C>0$ such that
  20 $$\begin{aligned} \left| \int \text {d}x\, V(x) f_{\ell }(x) - \frac{4\pi }{\log (e^N\ell /{{\mathfrak {a}}})} \right| \le \frac{C}{\log ^2(e^N\ell /{{\mathfrak {a}}})} \end{aligned}$$
(iv) There exists a constant $C>0$ such that
  21 $$\begin{aligned} \begin{aligned} |w_{\ell }(x)|&\le \left\{ \begin{array}{ll} C \qquad &{}\text {if } |x| \le R_0 \\ C \, \frac{\log (e^N\ell /|x|) }{\log (e^N\ell /{{\mathfrak {a}}})} \quad &{} \text {if } R_0 \le |x|\le e^N \ell \end{array} \right. \\ |\nabla w_{\ell }(x)|&\le \frac{C}{\log (e^N\ell /{{\mathfrak {a}}})} \frac{1}{|x| + 1} \qquad \text {for all } \, |x| \le e^N \ell \end{aligned} \end{aligned}$$
(v) Let $w_{N,\ell }= 1- f_{N,\ell }$ with $f_{\ell , N}=f_\ell (e^N x)$. Then the Fourier coefficients of the function $w_{N,\ell }$ defined in (17) are such that
  22 $$\begin{aligned} |{{\widehat{w}}}_{N,\ell }(p)| \le \frac{C}{p^2 \log (e^N\ell /{{\mathfrak {a}}})}. \end{aligned}$$

### Proof

The proof of points (i)–(iv) is deferred in Appendix B. To prove point v) we use the scattering equation (18):

$$\begin{aligned} {{\widehat{w}}}_\ell (e^{-N}p) =\frac{e^{2N}}{2p^2}\sum _{q \in \varLambda ^*}\widehat{V}(e^{-N}(p-q))\widehat{f}_{N,\ell }(q) - \frac{e^{4N}}{p^2} \lambda _\ell \sum _{q\in \varLambda ^*}{\widehat{\chi }}_\ell (p-q)\widehat{f}_{N,\ell }(q). \end{aligned}$$

Using the fact that $e^{2N}\lambda _\ell \le C \ell ^{-2} |\ln (e^N\ell /{{\mathfrak {a}}})|^{-1}$ and that $0 \le f_\ell \le 1$, we end up with

$$\begin{aligned} \begin{aligned} |{{\widehat{w}}}_\ell (e^{-N}p)|&\le \frac{e^{2N}}{2p^2}\left[ \big |({\widehat{V}}(e^{-N}\cdot )*\widehat{f}_{N,\ell })(p)\big | + 2 e^{2N} \lambda _\ell \, \big | ({\widehat{\chi }}_\ell *\widehat{f}_{N,\ell })(p)\big |\right] \\&\le \frac{e^{2N}}{2p^2}\left[ \int V(x)f_{\ell }(x)dx + C \ell ^{-2} |\log (e^N\ell /{{\mathfrak {a}}})|^{-1} \,\int \chi _\ell (x)f_{\ell }(e^Nx)dx\right] \\&\le \frac{Ce^{2N}}{p^2\log (e^N\ell /{{\mathfrak {a}}})}. \end{aligned} \end{aligned}$$

$$\square $$

We now define ${\check{\eta }} : \varLambda \rightarrow {\mathbb {R}}$ through

23 $$\begin{aligned} {\check{\eta }}(x)= - N w_{N,\ell }(x) = -N w_\ell (e^N x) \,. \end{aligned}$$

With (21) we find

24 $$\begin{aligned} |{\check{\eta }}(x)| \le \left\{ \begin{array}{ll} C N \qquad &{}\text {if } |x| \le e^{-N} R_0 \\ C \log (\ell / |x| ) \qquad &{}\text {if } e^{-N} R_0 \le |x| \le \ell \end{array} \right. \end{aligned}$$

and in particular, recalling that $e^{-N}R_0 < \ell \le 1/2$,

25 $$\begin{aligned} |{\check{\eta }} (x)| \le C \max (N , \log (\ell / |x|)) \le C N \end{aligned}$$

for all $x \in \varLambda$. Using (24) we find

$$\begin{aligned} \Vert \eta \Vert ^2 = \Vert {\check{\eta }} \Vert ^2 \le C \int _{|x|\le \ell } |\log (\ell /|x|)|^2 d^2x \le C \ell ^2 \int _0^1 (\log r)^2 r dr \le C \ell ^2\,. \end{aligned}$$

In the following we choose $\ell =N^{-\alpha }$, for some $\alpha >0$ to be fixed later, so that

26 $$\begin{aligned} \Vert \eta \Vert \le C N^{-\alpha }\,. \end{aligned}$$

This choice of $\ell$ will be crucial for our analysis, as commented below. Notice, on the other hand, that the $H^1$-norms of $\eta$ diverge, as $N \rightarrow \infty$. From (23) and Lemma 1, part iv) we find

$$\begin{aligned} \begin{aligned} \Vert {\check{\eta }}\Vert ^2_{H_1} = \int _{|x|\le \ell } e^{2N} N^2 | (\nabla w_{\ell }) (e^N x )|^2 d^2x&= \int _{|x|\le e^{N}\ell } N^2 | \nabla w_{\ell }(x)|^2 d^2x \\&\le C \int _{|x|\le e^{N} \ell } \frac{1}{(|x|+1)^2}\,d^2x \le C N \end{aligned} \end{aligned}$$

for $N \in {\mathbb {N}}$ large enough. We denote with $\eta : \varLambda ^* \rightarrow {\mathbb {R}}$ the Fourier transform of ${\check{\eta }}$, or equivalently

27 $$\begin{aligned} \eta _p = -N \widehat{w}_{N,\ell } (p) = - N e^{-2N} \widehat{w}_\ell (p/e^N)\,. \end{aligned}$$

With (22) we can bound (since $\ell = N^{-\alpha }$)

28 $$\begin{aligned} |\eta _p| \le \frac{C}{|p|^2} \end{aligned}$$

for all $p \in \varLambda _+^*=2\pi {\mathbb {Z}}^2 \backslash \{0\}$, and for some constant $C>0$ independent of *N*, if *N* is large enough. From (26) we also have

29 $$\begin{aligned} \Vert \eta \Vert _\infty \le C N^{-\alpha }\,. \end{aligned}$$

Moreover, (18) implies the relation

30 $$\begin{aligned} \begin{aligned} p^2 \eta _p + \frac{N}{2} (\widehat{V} (./e^N) *\widehat{f}_{N,\ell }) (p) = Ne^{2N} \lambda _\ell ({\widehat{\chi }}_\ell * \widehat{f}_{N,\ell }) (p) \end{aligned} \end{aligned}$$

or equivalently, expressing also the other terms through the coefficients $\eta _p$,

31 $$\begin{aligned} \begin{aligned} p^2 \eta _p + \frac{N}{2} \widehat{V} (p/e^N)&+ \frac{1}{2} \sum _{q \in \varLambda ^*} \widehat{V} ((p-q)/e^N) \eta _q \\&\quad = Ne^{2N} \lambda _\ell {\widehat{\chi }}_\ell (p) +e^{2N} \lambda _\ell \sum _{q \in \varLambda ^*} {\widehat{\chi }}_\ell (p-q) \eta _q\,. \end{aligned} \end{aligned}$$

We will mostly use the coefficients $\eta _p$ with $p\ne 0$. Sometimes, however, it will be useful to have an estimate on $\eta _0$ (because Eq. (31) involves $\eta _0$). From (27) and Lemma 1, part iv) we find

32 $$\begin{aligned} |\eta _0| \le N \int _{|x|\le \ell } w_\ell (e^Nx) d^2x \le C \int _{|x|\le \ell } \log (\ell /|x|) d^2x + C N e^{-N} \le C \ell ^2\,. \end{aligned}$$

With the coefficients (27) we define the antisymmetric operator

33 $$\begin{aligned} B = \frac{1}{2} \sum _{p\in \varLambda ^*_+} \left( \eta _p b_p^* b_{-p}^* - {\bar{\eta }}_p b_p b_{-p} \right) \, \end{aligned}$$

and we consider the unitary operator

34 $$\begin{aligned} e^{B} = \exp \left[ \frac{1}{2} \sum _{p \in \varLambda ^*_+} \left( \eta _p b_p^* b_{-p}^* - {\bar{\eta }}_p b_p b_{-p} \right) \right] \,. \end{aligned}$$

We refer to operators of the form (34) as generalized Bogoliubov transformations. In contrast with the standard Bogoliubov transformations

35 $$\begin{aligned} e^{\widetilde{B}} = \exp \left[ \frac{1}{2} \sum _{p\in \varLambda ^*_+} \left( \eta _p a_p^* a_{-p}^* - {\bar{\eta }}_p a_p a_{-p} \right) \right] \end{aligned}$$

defined in terms of the standard creation and annihilation operators, operators of the form (34) leave the truncated Fock space $\mathcal{F}_+^{\le N}$ invariant. On the other hand, while the action of standard Bogoliubov transformation on creation and annihilation operators is explicitly given by

$$\begin{aligned} e^{-\widetilde{B}} a_p e^{\widetilde{B}} = \cosh (\eta _p) a_p + \sinh (\eta _p) a_{-p}^* \, \end{aligned}$$

there is no such formula describing the action of generalized Bogoliubov transformations.

Conjugation with (34) leaves the number of particles essentially invariant, as confirmed by the following lemma.

### Lemma 2

Assume *B* is defined as in (33), with $\eta \in \ell ^2 (\varLambda ^*)$ and $\eta _p = \eta _{-p}$ for all $p \in \varLambda ^*_+$. Then, for every $n \in {\mathbb {N}}$ there exists a constant $C > 0$ such that, on $\mathcal{F}_+^{\le N}$,

36 $$\begin{aligned} e^{-B} (\mathcal{N}_+ +1)^{n} e^{B} \le C e^{C \Vert \eta \Vert } (\mathcal{N}_+ +1)^{n} \,. \end{aligned}$$

as an operator inequality on $\mathcal{F}^{\le N}_+$.

The proof of (36) can be found in [6, Lemma 3.1] (a similar result has been previously established in [23]).

With the generalized Bogoliubov transformation $e^{B} : \mathcal{F}_+^{\le N} \rightarrow \mathcal{F}^{\le N}_+$, we define a new, renormalized, excitation Hamiltonian $\mathcal{G}_{N,\alpha } : \mathcal{F}^{\le N}_+ \rightarrow \mathcal{F}^{\le N}_+$ by setting

37 $$\begin{aligned} \mathcal{G}_{N,\alpha } = e^{-B} \mathcal{L}_N e^{B} = e^{-B} U_N H_N U_N^* e^{B}\,. \end{aligned}$$

In the next proposition, we collect important properties $\mathcal{G}_{N,\alpha }$. We will use the notation

38 $$\begin{aligned} \mathcal{K}= \sum _{p \in \varLambda ^*_+} p^2 a_p^* a_p \qquad \text {and } \quad \mathcal{V}_N = \frac{1}{2} \sum _{\begin{array}{c} p,q \in \varLambda _+^*, r \in \varLambda ^* : \\ r \not = -p, -q \end{array}} \widehat{V} (r/e^N) a_{p+r}^* a_q^* a_{q+r} a_p \end{aligned}$$

for the kinetic and potential energy operators, restricted on $\mathcal{F}_+^{\le N}$, and $\mathcal{H}_N = \mathcal{K}+ \mathcal{V}_N$. We also introduce a renormalized interaction potential $\omega _N\in L^\infty (\varLambda )$, which is defined as the function with Fourier coefficients ${\widehat{\omega }}_N$

39 $$\begin{aligned} {\widehat{\omega }}_N(p) := g_N \, {\widehat{\chi }}(p/N^\alpha )\,, \qquad g_N =2 N^{1-2\alpha } e^{2N} \lambda _\ell \end{aligned}$$

for any $p \in \varLambda ^*_+$, and

40 $$\begin{aligned} {\widehat{\omega }}_N(0)= g_N {\widehat{\chi }}(0) = \pi g_N\,. \end{aligned}$$

with ${{\widehat{\chi }}}(p)$ the Fourier coefficients of the characteristic function of the ball of radius one. From (19) and $\ell =N^{-\alpha }$ one has $|g_N|\le C$. Note in particular that the potential ${{\widehat{\omega }}}_N(p)$ decays on momenta of order $N^\alpha$, which are much smaller than $e^N$. From Lemma 1 parts (i) and (iii) we find

41 $$\begin{aligned} \big | {\widehat{\omega }}_N(0) - N \Vert V f_\ell \Vert _1 \big | \le \frac{C}{N}\,, \qquad \Big |\, {\widehat{\omega }}_N(0) - 4 \pi \left( 1 + \alpha \,\tfrac{\log N}{N} \right) \, \Big | \le \frac{ C }{N}\,. \end{aligned}$$

### Proposition 1

Let $V\in L^3({\mathbb {R}}^2)$ be compactly supported, pointwise non-negative and spherically symmetric. Let $\mathcal{G}_{N,\alpha }$ be defined as in (37) and define

42 $$\begin{aligned}&\mathcal{G}^{\text {eff}}_{N,\alpha } := \frac{1}{2} {\widehat{\omega }}_N(0) (N-1)\left( 1-\frac{\mathcal{N}_+}{N}\right) + \left[ 2 N\widehat{V} (0)-\frac{1}{2} {\widehat{\omega }}_N(0) \right] \, \mathcal{N}_+ \, \left( 1-\frac{\mathcal{N}_+}{N}\right) \nonumber \\&\quad + \frac{1}{2} \sum _{ p\in \varLambda _+^*}{\widehat{\omega }}_N(p)(b_pb_{-p}+\text{ h.c.}) + \sqrt{N} \sum _{\begin{array}{c} p,q \in \varLambda ^*_+ :\\ p + q \not = 0 \end{array}} \widehat{V} (p/e^N) \left[ b_{p+q}^* a_{-p}^* a_q + \text{ h.c. }\right] \nonumber \\&\quad +\mathcal{H}_N \,. \end{aligned}$$

Then there exists a constant $C > 0$ such that $\mathcal{E}_{\mathcal{G}}= \mathcal{G}_{N,\alpha } - \mathcal{G}^\text {eff}_{N,\alpha }$ is bounded by

43 $$\begin{aligned} \begin{aligned} | \langle \xi , \mathcal{E}_{\mathcal{G}}\, \xi \rangle | \le \;&C \big ( N^{1/2 -\alpha } + N^{-1}(\log N)^{1/2} \big ) \Vert \mathcal{H}_N^{1/2}\xi \Vert \Vert (\mathcal{N}_++1)^{1/2}\xi \Vert \\&\quad + C N^{1-\alpha }\Vert (\mathcal{N}_++1)^{1/2}\xi \Vert ^2 +C \Vert \xi \Vert ^2 \end{aligned} \end{aligned}$$

for all $\alpha >1$, $\xi \in \mathcal{F}_+^{\le N}$ and $N\in {\mathbb {N}}$ large enough.

The proof of Proposition 1 is very similar to the proof of [3, Prop. 4.2]. For completeness, we discuss the changes in Appendix A.

## Cubic Renormalization

Conjugation with the generalized Bogoliubov transformation (35) renormalizes constant and off-diagonal quadratic terms on the r.h.s. of (42). In order to estimate the number of excitations $\mathcal{N}_+$ through the energy and show Bose–Einstein condensation, we still need to renormalize the diagonal quadratic term (the part proportional to $N \widehat{V} (0) \mathcal{N}_+$, on the first line of (42)) and the cubic term on the second line of (42). To this end, we conjugate $\mathcal{G}_{N,\alpha }^{\text {eff}}$ with an additional unitary operator, given by the exponential of the anti-symmetric operator

44 $$\begin{aligned} A := \frac{1}{\sqrt{N}} \sum _{r, v \in \varLambda ^*_+} \eta _r \big [b^*_{r+v}a^*_{-r}a_v - \text {h.c.}\big ] \end{aligned}$$

with $\eta _p$ defined in (27).

An important observation is that while conjugation with $e^A$ allows to renormalize the large terms in $\mathcal{G}_{N,\alpha }$, it does not substantially change the number of excitations. The following proposition can be proved similarly to [4, Proposition 5.1].

### Proposition 2

Suppose that *A* is defined as in (44). Then, for any $k\in {\mathbb {N}}$ there exists a constant $C >0$ such that the operator inequality

$$\begin{aligned} e^{-A} (\mathcal{N}_++1)^k e^{A} \le C (\mathcal{N}_+ +1)^k \end{aligned}$$

holds true on $\mathcal{F}_+^{\le N}$, for any $\alpha > 0$ (recall the choice $\ell = N^{-\alpha }$ in the definition (27) of the coefficients $\eta _r$), and *N* large enough.

We will also need to control the growth of the expectation of the energy $\mathcal{H}_N$ with respect to the cubic conjugation. This is the content of the following proposition, which is proved in Sect. 6.1.

### Proposition 3

Let *A* be defined as in (44). Then there exists a constant $C > 0$ such that

45 $$\begin{aligned} e^{-sA} \mathcal{H}_N e^{sA} \le C \mathcal{H}_N + C N (\mathcal{N}_+ +1) \end{aligned}$$

for all $\alpha \ge 1$, $s \in [0;1]$ and $N \in {\mathbb {N}}$ large enough.

We use now the cubic phase $e^{A}$ to introduce a new excitation Hamiltonian, obtained by conjugating the main part $\mathcal{G}_{N, \alpha }^{\text {eff}}$ of $\mathcal{G}_{N,\alpha }$. We define

46 $$\begin{aligned} \mathcal{R}_{N, \alpha }:= e^{-A} \,\mathcal{G}_{N,\alpha }^{\text {eff}}\,e^{A} \end{aligned}$$

on a dense subset of $\mathcal{F}_+^{\le N}$. Conjugation with $e^{A}$ renormalizes both the contribution proportional to $\mathcal{N}_+$ (in the first line on the r.h.s. of (42)) and the cubic term on the r.h.s. of (42), effectively replacing the singular potential $\widehat{V} (p/e^N)$ by the renormalized potential ${{\widehat{\omega }}}_N(p)$ defined in (39). This follows from the following proposition.

### Proposition 4

Let $V\in L^3({\mathbb {R}}^2)$ be compactly supported, pointwise non-negative and spherically symmetric. Let $\mathcal{R}_{N,\alpha }$ be defined in (46) and define

47 $$\begin{aligned} \begin{aligned} \mathcal{R}_{N,\alpha }^{\text {eff}}=&\; \frac{1}{2} (N-1)\, {{\widehat{\omega }}}_N(0) (1-\mathcal{N}_+/N) + \frac{1}{2} {{\widehat{\omega }}}_N(0) \,\mathcal{N}_+ \left( 1 - \mathcal{N}_+/N \right) \\&+ {{\widehat{\omega }}}_N(0) \sum _{p\in \varLambda ^*_+}a^*_pa_p \Big (1-\frac{\mathcal{N}_+}{N} \Big )+ \frac{1}{2} \sum _{p\in \varLambda ^*_+} {\widehat{\omega }}_N(p)\big [ b^*_p b^*_{-p} + b_p b_{-p} \big ] \\&+\frac{1}{\sqrt{N}} \sum _{\begin{array}{c} r,v\in \varLambda ^*_+:\\ r\ne -v \end{array} } {\widehat{\omega }}_N(r)\big [ b^*_{r+v}a^*_{-r} a_v + \text {h.c.}\big ] + \mathcal{H}_N \,. \end{aligned} \end{aligned}$$

Then for $\ell =N^{-\alpha }$ and $\alpha >2$ there exists a constant $C>0$ such that $\mathcal{E}_\mathcal{R}= \mathcal{R}_{N,\alpha }- \mathcal{R}_{N,\alpha }^{\text {eff}}$ is bounded by

48 $$\begin{aligned} \pm \mathcal{E}_\mathcal{R}\le C [ N^{2-\alpha } + N^{-1/2} (\log N)^{1/2} ](\mathcal{H}_N +1) \, , \end{aligned}$$

for $N \in {\mathbb {N}}$ sufficiently large.

The proof of Proposition 4 will be given in Sect. 6. We will also need more detailed information on $\mathcal{R}_{N,\alpha }^{\text {eff}}$, as contained in the following proposition.

### Proposition 5

Let $\mathcal{R}_{N,\alpha }^{\text {eff}}$ be defined in (47). Then, for every $c > 0$ there is a constant $C > 0$ (large enough) such that

49 $$\begin{aligned} \begin{aligned} \mathcal{R}_{N,\alpha }^{\text {eff}}\ge&\;2\pi N + \frac{{\widehat{\omega }}_N (0)}{2} \,\mathcal{N}_+ + \frac{c}{\log N}\, \mathcal{H}_N - C (\log N)^2 \, \frac{\,\mathcal{N}_+^2}{N}\, - C \end{aligned}\end{aligned}$$

for all $\alpha >2$ and $N \in {\mathbb {N}}$ large enough.

Moreover, let $f,g : {\mathbb {R}}\rightarrow [0;1]$ be smooth, with $f^2 (x) + g^2 (x) =1$ for all $x \in {\mathbb {R}}$. For $M \in {\mathbb {N}}$, let $f_M := f(\mathcal{N}_+/M)$ and $g_M:= g(\mathcal{N}_+/M)$. Then there exists $C > 0$ such that

50 $$\begin{aligned} \mathcal{R}_{N,\alpha }^{\text {eff}} = f_M\, \mathcal{R}_{N, \alpha }^{\text {eff}}\, f_M + g_M\, \mathcal{R}_{N, \alpha }^{\text {eff}}\, g_M + \varTheta _{M} \end{aligned}$$

with

$$\begin{aligned} \pm \varTheta _M \le \frac{C\log N}{M^2}\big (\Vert f'\Vert ^2_{\infty } +\Vert g'\Vert ^2_{\infty }\big ) \big ( \mathcal{H}_N +1 \big ) \end{aligned}$$

for all $\alpha > 2$, $M \in {\mathbb {N}}$ and $N \in {\mathbb {N}}$ large enough.

### Proof

From (47), using that $| {{\widehat{\omega }}}_N(0) |\le C$ we have

51 $$\begin{aligned} \begin{aligned} \mathcal{R}_{N,\alpha }^{\text {eff}} \ge&\; \frac{N}{2} \, {{\widehat{\omega }}}_N(0) + {{\widehat{\omega }}}_N(0) \,\mathcal{N}_+ + \frac{1}{2} \sum _{p\in \varLambda ^*_+} {\widehat{\omega }}_N(p)\big [ b^*_p b^*_{-p} + b_p b_{-p} \big ] \\&+\frac{1}{\sqrt{N}} \sum _{\begin{array}{c} r,v\in \varLambda ^*_+:\\ r\ne -v \end{array} } {\widehat{\omega }}_N(r)\big [ b^*_{r+v}a^*_{-r} a_v + \text {h.c.}\big ] + \mathcal{H}_N - C \,\frac{\mathcal{N}_+^2}{N} - C\,. \end{aligned} \end{aligned}$$

For the cubic term on the r.h.s. of (51), with

52 $$\begin{aligned} \sum _{p \in \varLambda ^*_+} \frac{|{{\widehat{\omega }}}_N(p)|^2}{p^2} \le C \log N \end{aligned}$$

we can bound

53 $$\begin{aligned} \begin{aligned} \Big | \frac{1}{\sqrt{N}}&\sum _{\begin{array}{c} r,v\in \varLambda ^*_+\\ r \ne -v \end{array}} {\widehat{\omega }}_N(r) \langle \xi , b^*_{r+v} a^*_{-r} a_v\xi \rangle \Big | \\&\le \frac{1}{\sqrt{N}} \sum _{\begin{array}{c} r,v\in \varLambda ^*_+\\ r \ne -v \end{array}}|{\widehat{\omega }}_N(r) | \Vert (\mathcal{N}_+ + 1)^{-1/2} b_{r+v} a_{-r} \xi \Vert \Vert (\mathcal{N}_+ + 1)^{1/2} a_v \xi \Vert \\&\le \frac{1}{\sqrt{N}} \, \bigg [ \sum _{\begin{array}{c} r,v\in \varLambda ^*_+\\ r \ne -v \end{array}} |r|^2 \Vert (\mathcal{N}_++1)^{-1/2}b_{r+v} a_{-r} \xi \Vert ^2 \bigg ]^{1/2}\\&\quad \times \bigg [ \sum _{\begin{array}{c} r,v\in \varLambda ^*_+\\ r \ne -v \end{array}} \frac{|{\widehat{\omega }}_N(r) |^2}{|r|^2} \Vert (\mathcal{N}_++1)^{1/2}a_v \xi \Vert ^2 \bigg ]^{1/2}\\&\le \frac{C (\log N)^{1/2}}{\sqrt{N}} \,\Vert \mathcal{K}^{1/2}\xi \Vert \Vert (\mathcal{N}_++1)\xi \Vert \,. \end{aligned}\end{aligned}$$

As for the off-diagonal quadratic term on the r.h.s of (51), we combine it with part of the kinetic energy to estimate. For any $0< \mu < 1$, we have

54 $$\begin{aligned} \begin{aligned} \frac{1}{2} \sum _{p \in \varLambda _+^*} {\widehat{\omega }}_N (p)&\big [ b_p^* b_{-p}^* + b_{-p} b_p \big ] + (1- \mu ) \sum _{p \in \varLambda ^*_+} p^2 a_p^* a_p \\ = \;&(1-\mu ) \sum _{p \in \varLambda _+^*} p^2 \left[ b_p^* + \frac{ {\widehat{\omega }}_N (p)}{2(1-\mu ) p^2} b_{-p} \right] \left[ b_p + \frac{ {\widehat{\omega }}_N (p)}{2(1-\mu ) p^2} b^*_{-p} \right] \\&- \frac{1}{4(1-\mu )} \sum _{p \in \varLambda ^*_+} \frac{|{\widehat{\omega }}_N (p)|^2}{p^2} b_p b_p^* + (1-\mu ) \sum _{p \in \varLambda ^*_+} p^2 a_p^* \frac{\mathcal{N}_+}{N} a_p \end{aligned} \end{aligned}$$

since $a_p^* a_p - b_p^* b_p = a_p^* (\mathcal{N}_+ / N) a_p$. With (14), we conclude that

$$\begin{aligned} \begin{aligned} \frac{1}{2} \sum _{p \in \varLambda _+^*} {\widehat{\omega }}_N (p)&\big [ b_p^* b_{-p}^* + b_{-p} b_p \big ] + (1- \mu ) \sum _{p \in \varLambda ^*_+} p^2 a_p^* a_p \\ \ge \;&- \frac{1}{4(1-\mu )} \sum _{p \in \varLambda ^*_+} \frac{|{\widehat{\omega }}_N (p)|^2}{p^2} a^*_p a_p - \frac{1}{4(1-\mu )} \sum _{p \in \varLambda ^*_+} \frac{|{\widehat{\omega }}_N (p)|^2}{p^2}\,. \end{aligned} \end{aligned}$$

With the choice $\mu = C / \log N$ and with (52), we obtain

55 $$\begin{aligned} \begin{aligned} \frac{1}{2} \sum _{p \in \varLambda _+^*} \widehat{\omega }_N (p)&\big [ b_p^* b_{-p}^* + b_{-p} b_p \big ] + (1- \mu ) \sum _{p \in \varLambda ^*_+} p^2 a_p^* a_p \\\ge&- \frac{1}{4(1-\mu )} \sum _{p \in \varLambda ^*_+}\frac{| \widehat{\omega }_N (p)|^2}{p^2} a^*_p a_p - \frac{1}{4} \sum _{p \in \varLambda ^*_+} \frac{| \widehat{\omega }_N (p)|^2}{p^2} - C \,. \end{aligned} \end{aligned}$$

To bound the first terms on the r.h.s. of the last equation, we use the term ${\widehat{\omega }}_N (0) \mathcal{N}_+$, in (51). To this end, we observe that, with (41),

$$\begin{aligned} \frac{|{\widehat{\omega }}_N (p)|^2}{4 (1-\mu ) p^2} \le \frac{|{\widehat{\omega }}_N (0)|^2}{4 (1-\mu ) p^2} \le \frac{{\widehat{\omega }}_N (0)}{4 ( 1-\mu ) \pi } \left( 1 + C \frac{\log N}{N} \right) \le \frac{{\widehat{\omega }}_N (0)}{2} \end{aligned}$$

for every $p \in \varLambda ^*_+$ (notice that $|p| \ge 2\pi$, for every $p \in \varLambda ^*_+$) and for *N* large enough (recall the choice $\mu = C / \log N$). Inserting (53) and (55) in (51) and using the kinetic energy $\mu \mathcal{K}= C (\log N)^{-1} \mathcal{K}$ (remaining after subtracting the term $(1-\mu ) \mathcal{K}$ needed on the l.h.s. of (55)) to bound the r.h.s. of (53), we find

56 $$\begin{aligned} \begin{aligned} \mathcal{R}_{N,\alpha }^{\text {eff}} \ge&\; \frac{N}{2} \, {{\widehat{\omega }}}_N(0) - \frac{1}{4} \sum _{p \in \varLambda _+^*} \frac{|{\widehat{\omega }}_N (p)|^2}{p^2} + \frac{{{\widehat{\omega }}}_N(0)}{2} \,\mathcal{N}_+ + \frac{c}{\log N} \mathcal{H}_N \\&- C \frac{(\log N)^2}{N} \mathcal{N}_+^2 - C. \end{aligned} \end{aligned}$$

Let us now consider the second term on the r.h.s more carefully. Using that, from (39), ${\widehat{\omega }}_N (p) = g_N {\widehat{\chi }} (p/N^\alpha )$, we can bound, for any fixed $K > 0$,

$$\begin{aligned} \frac{1}{4} \sum _{p \in \varLambda _+^*} \frac{|{\widehat{\omega }}_N (p)|^2}{p^2} \le C + \frac{1}{4} \sum _{\begin{array}{c} p \in \varLambda _+^* : \\ K < |p| \le N^\alpha \end{array}} \frac{|{\widehat{\omega }}_N (p)|^2}{p^2}\,. \end{aligned}$$

With $|{\widehat{\omega }}_N (p) - {\widehat{\omega }}_N (0)|\le C |p|/ N^\alpha$, we obtain

57 $$\begin{aligned} \frac{1}{4} \sum _{p \in \varLambda _+^*} \frac{|{\widehat{\omega }}_N (p)|^2}{p^2} \le C + \frac{|{\widehat{\omega }}_N (0)|^2}{4} \sum _{\begin{array}{c} p \in \varLambda _+^* : \\ K< |p| \le N^\alpha \end{array}} \frac{1}{p^2} \le C + 4 \pi ^2 \sum _{\begin{array}{c} p \in \varLambda ^*_+ : \\ K < |p| \le N^\alpha \end{array}} \frac{1}{p^2}\,. \end{aligned}$$

For $q \in {\mathbb {R}}^2$, let us define $h(q) = 1/p^2$, if *q* is contained in the square of side length $2\pi$ centered at $p \in \varLambda ^*_+$ (with an arbitrary choice on the boundary of the squares). We can then estimate, for *K* large enough,

$$\begin{aligned} 4\pi ^2 \sum _{\begin{array}{c} p \in \varLambda _+^* : \\ K< |p| \le N^\alpha \end{array}} \frac{1}{p^2} \le \int _{K/2 < |q| \le N^\alpha + K} h(q) dq\,. \end{aligned}$$

For *q* in the square centered at $p \in \varLambda ^*_+$, we bound

$$\begin{aligned} \left| h(q) - \frac{1}{q^2} \right| = \frac{|p^2 - q^2|}{p^2 \,q^2} \le \frac{C}{|q|^3}\,. \end{aligned}$$

Hence

$$\begin{aligned} 4 \pi ^2 \sum _{\begin{array}{c} p \in \varLambda _+^* : \\ K< |p| \le N^\alpha \end{array}} \frac{1}{p^2} \le \int _{K/2< |q| < N^\alpha + K} \frac{1}{q^2} dq + C \le 2\pi \alpha \log N + C\,. \end{aligned}$$

Inserting in (57), we conclude that

$$\begin{aligned} \frac{1}{4} \sum _{p \in \varLambda _+^*} \frac{|{\widehat{\omega }}_N (p)|^2}{p^2} \le 2\pi \alpha \log N +C\,. \end{aligned}$$

Combining the last bound with (41) (and noticing that the contribution proportional to $\log N$ cancels exactly), from (56) we obtain

$$\begin{aligned} \mathcal{R}_{N,\alpha }^{\text {eff}} \ge \; 2\pi N + \frac{\widehat{\omega }_N(0)}{2} \,\mathcal{N}_+ + \frac{c}{\log N} \mathcal{H}_N - C \frac{(\log N)^2}{N} \mathcal{N}_+^2 - C \end{aligned}$$

which proves (49).

Next we prove (50). From (47), with $|\widehat{\omega }_N(0)|\le C$, the bound (53) and since, by (52),

$$\begin{aligned} \begin{aligned} \left| \sum _{p \in \varLambda _+^*} {\widehat{\omega }}_N (p) \langle \xi , b_p^* b_{-p}^* \xi \rangle \right|&\le \sum _{p \in \varLambda _+^*} |{\widehat{\omega }}_N (p)| \Vert b_p \xi \Vert \Vert (\mathcal{N}_+ + 1)^{1/2} \xi \Vert \\&\le \left[ \sum _{p \in \varLambda ^*_+} \frac{|{\widehat{\omega }}_N (p)|^2}{p^2} \right] ^{1/2} \Vert (\mathcal{N}_+ + 1)^{1/2} \xi \Vert \Vert \mathcal{K}^{1/2} \xi \Vert \\&\le C (\log N)^{1/2} \Vert (\mathcal{N}_+ + 1)^{1/2} \xi \Vert \Vert \mathcal{K}^{1/2} \xi \Vert \end{aligned} \end{aligned}$$

it follows that

58 $$\begin{aligned} \mathcal{R}_{N,\alpha }^{\text {eff}} = 2\pi N +\mathcal{H}_N + \theta _{N,\alpha } \end{aligned}$$

where for arbitrary $\delta > 0$, there exists a constant $C>0$ such that

59 $$\begin{aligned} \pm \theta _{N,\alpha } \le \delta \mathcal{H}_N + C (\log N) \,(\mathcal{N}_+ +1) \,. \end{aligned}$$

We now note that for $f: {\mathbb {R}}\rightarrow {\mathbb {R}}$ smooth and bounded and $\theta _{N,\alpha }$ defined above, there exists a constant $C>0$ such that

60 $$\begin{aligned} \pm [f(\mathcal{N}_+/M), [f(\mathcal{N}_+/M), \theta _{N,\alpha }]] \le C\, \frac{\log N}{M^{2}} \Vert f' \Vert _\infty ^2 (\mathcal{H}_N +1) \end{aligned}$$

for all $\alpha >2$ and $N \in {\mathbb {N}}$ large enough. The proof of (60) follows analogously to the one for (59), since the bounds leading to (59) remain true if we replace the operators $b_p^\#$, $\#=\{ \cdot , *\}$, and $a_p^* a_q$ with $[f(\mathcal{N}_+/M), [f(\mathcal{N}_+/M), b^\#_p ]]$ or $[f(\mathcal{N}_+/M), [f(\mathcal{N}_+/M), a^*_p a_q ]]$ respectively, provided we multiply the r.h.s. by an additional factor $M^{-2}\Vert f'\Vert _\infty ^2$, since, for example

$$\begin{aligned}{}[f(\mathcal{N}_+/M), [f(\mathcal{N}_+/M), b_p ]] = \big ( f(\mathcal{N}_+/M) - f((\mathcal{N}_++1)/M) \big )^2 b_p \end{aligned}$$

and $\Vert f(\mathcal{N}_+/M) - f((\mathcal{N}_++1)/M) \Vert \le C M^{-1} \Vert f'\Vert _\infty$. With an explicit computation we obtain

$$\begin{aligned} \begin{aligned} \mathcal{R}_{N,\alpha }^{\text {eff}}=f_M \mathcal{R}_{N,\alpha }^{\text {eff}}f_M +g_M \mathcal{R}_{N,\alpha }^{\text {eff}}g_M+\frac{1}{2}\Big ([f_M,[f_M,\mathcal{R}^{\text {eff}}_{N,\alpha }]]+[g_M,[g_M,\mathcal{R}^{\text {eff}}_{N,\alpha }]]\Big ) \end{aligned} \end{aligned}$$

Writing $\mathcal{R}_{N,\alpha }^{\text {eff}}$ as in (58) and using (60) we get

$$\begin{aligned} \begin{aligned} \pm \Big ([f_M,[f_M,\mathcal{R}_{N,\alpha }^{\text {eff}}]]+[g_M,[g_M,\mathcal{R}_{N,\alpha }^{\text {eff}}]]\Big )\le \frac{C\log N}{M^2} \big (\Vert f'\Vert ^2_{\infty } +\Vert g'\Vert ^2_{\infty }\big ) \big ( \mathcal{H}_N +1 \big )\,. \end{aligned} \end{aligned}$$

$$\square $$

## Proof of Theorem 1

The next proposition combines the results of Propositions 1, 4 and 5. Its proof makes use of localization in the number of particle and is an adaptation of the proof of [4, Proposition 6.1]. The main difference w.r.t. [4] is that here we need to localize on sectors of $\mathcal{F}^{\le N}$ where the number of particles is *o*(*N*), in the limit $N \rightarrow \infty$.

### Proposition 6

Let $V\in L^3({\mathbb {R}}^2)$ be compactly supported, pointwise non-negative and spherically symmetric. Let $\mathcal{G}_{N,\alpha }$ be the renormalized excitation Hamiltonian defined as in (37). Then, for every $\alpha \ge 5/2$, there exist constants $C,c > 0$ such that

61 $$\begin{aligned} \mathcal{G}_{N,\alpha } -2\pi N \ge c\, \mathcal{N}_+ - C \end{aligned}$$

for all $N \in {\mathbb {N}}$ sufficiently large.

### Proof

Let $f,g: {\mathbb {R}}\rightarrow [0;1]$ be smooth, with $f^2 (x) + g^2 (x)= 1$ for all $x \in {\mathbb {R}}$. Moreover, assume that $f (x) = 0$ for $x > 1$ and $f (x) = 1$ for $x < 1/2$. For a small $\varepsilon >0$, we fix $M = N^{1-\varepsilon }$ and we set $f_M = f (\mathcal{N}_+ / M), g_M = g (\mathcal{N}_+ / M)$. It follows from Proposition 5 that

62 $$\begin{aligned} \begin{aligned} \mathcal{R}_{N,\alpha }^{\text {eff}} -2\pi N \ge \;&f_M \big (\mathcal{R}^{\text {eff}}_{N,\alpha } -2\pi N \big ) f_M + g_M \big (\mathcal{R}^{\text {eff}}_{N,\alpha } -2\pi N \big ) g_M \\&- CN^{2\varepsilon -2} (\log N) (\mathcal{H}_N + 1) \end{aligned} \end{aligned}$$

Let us consider the first term on the r.h.s. of (62). From Proposition 5, for all $\alpha >2$ there exist $c, C>0$ such that

63 $$\begin{aligned} \mathcal{R}_{N,\alpha }^{\text {eff}} -2\pi N \ge c\, \mathcal{N}_+ - \frac{C}{N} \,(\log N)^2 \, \mathcal{N}_+^{\,2} - C \,. \end{aligned}$$

On the other hand, with (58) and (59) we also find

64 $$\begin{aligned} \mathcal{R}_{N,\alpha }^{\text {eff}} -2\pi N \ge c \mathcal{H}_N - C (\log N)\,(\mathcal{N}_++1) \end{aligned}$$

for all $\alpha > 2$ and *N* large enough. Moreover, due to the choice $M=N^{1-\varepsilon }$, we have

$$\begin{aligned} \frac{(\log N)^2}{N} f_M \mathcal{N}_+^2 f_M \le \frac{(\log N)^{2} }{N^\varepsilon } f^2_M \mathcal{N}_+ \,. \end{aligned}$$

With the last bound, Eq. (63) implies that

65 $$\begin{aligned} f_M \Big (\mathcal{R}^{\text {eff}}_{N,\alpha } -2\pi N\Big ) f_M \ge c f^2_M \mathcal{N}_+ - C \end{aligned}$$

for *N* large enough.

Let us next consider the second term on the r.h.s. of (62). We claim that there exists a constant $c > 0$ such that

66 $$\begin{aligned} g_M \Big (\mathcal{R}^{\text {eff}}_{N,\alpha } -2\pi N \Big ) g_M \ge c N g_M^2 \end{aligned}$$

for all *N* sufficiently large. To prove (66) we observe that, since $g(x) = 0$ for all $x \le 1/2$,

$$\begin{aligned} \begin{aligned} g_M \Big ( \mathcal{R}^{\text {eff}}_{N,\alpha } -2\pi N&\Big ) g_M \ge \left[ \inf _{\xi \in \mathcal{F}_{\ge M/2}^{\le N} : \Vert \xi \Vert = 1} \frac{1}{N} \langle \xi , \mathcal{R}^{\text {eff}}_{N,\alpha } \xi \rangle - 2\pi \right] N g_M^2 \end{aligned} \end{aligned}$$

where $\mathcal{F}_{\ge M/2}^{\le N} = \{ \xi \in \mathcal{F}_+^{\le N} : \xi = \chi (\mathcal{N}_+ \ge M/2) \xi \}$ is the subspace of $\mathcal{F}_+^{\le N}$ where states with at least *M*/2 excitations are described (recall that $M = N^{1-\varepsilon }$). To prove (66) it is enough to show that there exists $C > 0$ with

67 $$\begin{aligned} \inf _{\xi \in \mathcal{F}_{\ge M/2}^{\le N} : \Vert \xi \Vert = 1} \frac{1}{N} \langle \xi , \mathcal{R}^{\text {eff}}_{N,\alpha } \xi \rangle -2\pi \ge C \end{aligned}$$

for all *N* large enough. On the other hand, using the definitions of $\mathcal{G}_{N,\alpha }$ in (42), $\mathcal{R}_{N,\alpha }$ and $\mathcal{R}^{\text {eff}}_{N,\alpha }$ in (47), we obtain that the ground state energy $E_N$ of the system is given by

$$\begin{aligned} \begin{aligned} E_N&= \inf _{\xi \in \mathcal{F}_+^{\le N} : \Vert \xi \Vert =1} {\bigl \langle {\xi , e^{-A}\mathcal{G}_{N,\alpha }e^A \xi }\bigr \rangle }= \inf _{\xi \in \mathcal{F}_+^{\le N} : \Vert \xi \Vert =1} {\bigl \langle {\xi , \big (\mathcal{R}^{\text {eff}}_{N,\alpha } + \mathcal{E}_L \big ) \xi }\bigr \rangle } \end{aligned} \end{aligned}$$

with $\mathcal{E}_{L} = \mathcal{E}_{\mathcal{R}} +e^{-A} \mathcal{E}_{\mathcal{G}}e^A$. The bounds (43) and (48), together with Propositions 2 and 3, imply that for any $\alpha \ge 5/2$ there exists $C>0$ such that

$$\begin{aligned} \begin{aligned} \pm \mathcal{E}_{L}&\le C N^{-1/2}(\log N)^{1/2} \big [ ( \mathcal{H}_N +1 ) + e^{-A}\big ( N^{-1}(\mathcal{H}_N+1) + (\mathcal{N}_++1) \big ) e^A \big ] + C \\&\le C N^{-1/2}(\log N)^{1/2} ( \mathcal{H}_N +1 ) + C \end{aligned} \end{aligned}$$

With (64) we obtain

68 $$\begin{aligned} \pm \mathcal{E}_L \le C N^{-1/2} (\log N)^{1/2} \big (\mathcal{R}^{\text {eff}}_{N,\alpha } -2\pi N \big ) + C N^{-1/2} (\log N)^{3/2} \mathcal{N}_+ + C \,, \end{aligned}$$

and therefore, with $\mathcal{N}_+\le N$

$$\begin{aligned} E_N - 2\pi N \le C \inf _{\xi \in \mathcal{F}_+^{\le N} : \Vert \xi \Vert =1} {\bigl \langle {\xi , \big (\mathcal{R}^{\text {eff}}_{N,\alpha } - 2 \pi N \big ) \xi }\bigr \rangle } +C N^{1/2} (\log N)^{3/2} + C\,. \end{aligned}$$

From the result (3) of [13, 14, 16]

$$\begin{aligned} \begin{aligned} \inf _{\xi \in \mathcal{F}_{\ge M/2}^{\le N} : \Vert \xi \Vert = 1} \frac{1}{N} \langle \xi , \mathcal{R}^{\text {eff}}_{N,\alpha } \xi \rangle -2 \pi&\ge \inf _{\xi \in \mathcal{F}_{+}^{\le N} : \Vert \xi \Vert = 1} \frac{1}{N} \langle \xi , \big (\mathcal{R}^{\text {eff}}_{N,\alpha } - 2 \pi N \big ) \xi \rangle \\&\ge c \left( \frac{E_N}{N} -2\pi \right) - \frac{C}{\sqrt{N}}\,(\log N)^{3/2} - C N^{-1} \rightarrow 0 \end{aligned} \end{aligned}$$

as $N \rightarrow \infty$. If we assume by contradiction that (67) does not hold true, then we can find a subsequence $N_j \rightarrow \infty$ with

$$\begin{aligned} \inf _{\xi \in \mathcal{F}_{\ge M_j/2}^{\le N_j} : \Vert \xi \Vert = 1} \frac{1}{N_j} \langle \xi , \mathcal{R}_{N_j ,\alpha }^{\text {eff}} \xi \rangle -2\pi \rightarrow 0 \end{aligned}$$

as $j \rightarrow \infty$ (here we used the notation $M_j = N_j^{1-\varepsilon }$). This implies that there exists a sequence ${\tilde{\xi }}_{N_j} \in \mathcal{F}^{\le N_j}_{ \ge M_j /2}$ with $\Vert {\tilde{\xi }}_{N_j} \Vert = 1$ for all $j \in {\mathbb {N}}$ such that

$$\begin{aligned} \lim _{j \rightarrow \infty } \frac{1}{N_j} \langle {\tilde{\xi }}_{N_j}, \mathcal{R}^{\text {eff}}_{N_j, \alpha } {\tilde{\xi }}_{N_j} \rangle = 2\pi \, . \end{aligned}$$

On the other hand, using the relation $\mathcal{R}^{\text {eff}}_{N_j,\alpha } = e^{-A}\mathcal{G}_{N_j,\alpha } e^A - \mathcal{E}_{L,j}$ with $\mathcal{E}_{L,j}$ satisfying the bound (68) (with $\mathcal{N}_+ \le N_j$), we obtain that there exist constants $c_1, c_2, C>0$ such that

$$\begin{aligned} \begin{aligned}&c_1 \langle {\tilde{\xi }}_{N_j}, \big ( \mathcal{R}^{\text {eff}}_{N,\alpha }- 2 \pi N_j \big ) {\tilde{\xi }}_{N_j} \rangle - C N_j^{1/2} (\log N_j)^{3/2} \\&\quad \le \langle e^{A} {\tilde{\xi }}_{N_j}, \big (\mathcal{G}_{N_j,\alpha } -\; 2 \pi N_j \big ) e^A {\tilde{\xi }}_{N_j} \rangle \\&\quad \le c_2 \langle {\tilde{\xi }}_{N_j}, \big (\mathcal{R}^{\text {eff}}_{N,\alpha } - 2 \pi N_j \big ) {\tilde{\xi }}_{N_j} \rangle + C N_j^{1/2} (\log N_j)^{3/2} \end{aligned} \end{aligned}$$

Hence for $\xi _{N_j} = e^{A} {\tilde{\xi }}_{N_j}$ we have

$$\begin{aligned} \lim _{N_j \rightarrow \infty } \frac{1}{N_j} \langle \xi _{N_j}, \mathcal{G}_{N_j,\alpha } \xi _{N_j} \rangle = 2\pi \,. \end{aligned}$$

Let now $S:= \{N_j: j\in {\mathbb {N}}\} \subset {\mathbb {N}}$ and denote by $\xi _N$ a normalized minimizer of $\mathcal{G}_{N,\alpha }$ for all $N\in {\mathbb {N}}\setminus S$. Setting $\psi _N = U_N^* e^{B} \xi _N$, for all $N \in {\mathbb {N}}$, we obtain that $\Vert \psi _N \Vert = 1$ and that

69 $$\begin{aligned} \lim _{N \rightarrow \infty } \frac{1}{N} \langle \psi _N, H_N \psi _N \rangle = \lim _{N \rightarrow \infty } \frac{1}{N} \langle \xi _N, \mathcal{G}_{N,\alpha } \xi _N \rangle = 2\pi \end{aligned}$$

Eq. (69) shows that the sequence $\psi _N$ is an approximate ground state of $H_N$. From (5), we conclude that $\psi _N$ exhibits complete Bose–Einstein condensation in the zero-momentum mode $\varphi _0$, and in particular that there exists ${{\bar{\delta }}} >0$ such that

$$\begin{aligned} \left| 1 - \langle \varphi _0, \gamma _N \varphi _0 \rangle \right| \le CN^{-{\bar{\delta }}}\,. \end{aligned}$$

Using Lemma 2, Proposition 2 and the rules (11), we observe that

70 $$\begin{aligned} \begin{aligned} \frac{1}{N} \langle \xi _N, \mathcal{N}_+ \xi _N \rangle&= \frac{1}{N} \langle e^{-B} U_N \psi _N , \mathcal{N}_+ e^{-B} U_N \psi _N \rangle \\&\le \frac{C}{N} \langle \psi _N , U_N^* (\mathcal{N}_+ +1) U_N \psi _N \rangle \\&= \frac{C}{N} + C \left[ 1 - \frac{1}{N} \langle \psi _N, a^* (\varphi _0) a(\varphi _0) \psi _N \rangle \right] \\&= \frac{C}{N} + C \left[ 1 - \langle \varphi _0 , \gamma _N \varphi _0 \rangle \right] \le CN^{-{\bar{\delta }}} \end{aligned} \end{aligned}$$

as $$N \rightarrow \infty $$.

On the other hand, for $N \in S = \{ N_j : j \in {\mathbb {N}}\}$, we have $\xi _N = \chi (\mathcal{N}_+ \ge M/2) \xi _N$ and therefore

$$\begin{aligned} \frac{1}{N} \langle \xi _N, \mathcal{N}_+ \xi _N \rangle \ge \frac{M}{2N} = \frac{N^{-\varepsilon }}{2}\,. \end{aligned}$$

Choosing $\varepsilon < {\bar{\delta }}$ and *N* large enough we get a contradiction with (70). This proves (67), (66) and therefore also

71 $$\begin{aligned} g_M \Big ( \mathcal{R}^{\text {eff}}_{N,\alpha } -2\pi N \Big ) g_M \ge c \mathcal{N}_+ g_M^2\,. \end{aligned}$$

Inserting (65) and (71) on the r.h.s. of (62), we obtain that

72 $$\begin{aligned} \mathcal{R}_{N,\alpha }^{\text {eff}} -2\pi N \ge c \mathcal{N}_+ - C (\log N ) N^{2\varepsilon -2} (\mathcal{H}_N + 1) - C \end{aligned}$$

for *N* large enough. With (64), (72) implies

$$\begin{aligned} \mathcal{R}^{\text {eff}}_{N,\alpha } -2\pi N \ge c \mathcal{N}_+ - C. \end{aligned}$$

To conclude, we use the relation $e^{-A}\mathcal{G}_{N,\alpha }e^A=\mathcal{R}^{\text {eff}}_{N,\alpha } + \mathcal{E}_L$ and the bound (68). We have that for $\alpha \ge 5/2$ there exist $c, C>0$ such that

$$\begin{aligned} \begin{aligned} \mathcal{G}_{N,\alpha } - 2 \pi N&\ge c e^A \big (\mathcal{R}^{\text {eff}}_{N,\alpha } -2\pi N \big ) e^{-A} - C N^{-1/2} (\log N)^{3/2} e^A \mathcal{N}_+ e^A - C \\&\ge c \,e^{A} \mathcal{N}_+ e^{-A} -C \ge c \mathcal{N}_+ - C \end{aligned}\end{aligned}$$

where we used (72) and Proposition 2. $\square$

We are now ready to show our main theorem.

### Proof of Theorem 1

Let $E_N$ be the ground state energy of $H_N$. Evaluating (42) and (43) on the vacuum $\varOmega \in \mathcal{F}^{\le N}_+$ and using (40), we obtain the upper bound

$$\begin{aligned} E_N \le 2\pi N+ C \log N \,. \end{aligned}$$

Notice that we cannot reach the expected optimal upper bound $E_N \le 2 \pi N + C$ because of the logarithmic correction in ${\hat{\omega }}_N (0)$ (see (40)). In the lower bound, this logarithmic factor is compensated by the contribution arising from the off-diagonal quadratic term, extracted starting from (54). To obtain the same term for the upper bound, we would have to modify our trial state (diagonalizing the quadratic terms in $\mathcal{R}_{N,\alpha }$); this, however, would produce even larger contributions arising from the potential energy.

With Eq. (61) we also find the lower bound $E_N \ge 2\pi N - C$. This proves (6).

Let now $\psi _N \in L^2_s (\varLambda ^N)$ with $\Vert \psi _N \Vert =1$ and

73 $$\begin{aligned} \langle \psi _N , H_N \psi _N \rangle \le 2\pi N + K\,. \end{aligned}$$

We define the excitation vector $\xi _N = e^{-B} U_N \psi _N$. Then $\Vert \xi _N \Vert = 1$ and, recalling that $\mathcal{G}_{N,\alpha } = e^{-B} U_N H_N U_N^* e^{B}$ we have, with (61),74From Eqs. (73) and (74) we conclude that

$$\begin{aligned} \langle \xi _N, \mathcal{N}_+ \xi _N \rangle \le C (1 +K)\,. \end{aligned}$$

If $\gamma _N$ denotes the one-particle reduced density matrix associated with $\psi _N$, using Lemma 2 we obtain

$$\begin{aligned} \begin{aligned} 1 - \langle \varphi _0, \gamma _N \varphi _0 \rangle&= 1 - \frac{1}{N} \langle \psi _N, a^* (\varphi _0) a (\varphi _0) \psi _N \rangle \\&= 1 - \frac{1}{N} \langle U_N^* e^{B} \xi _N, a^* (\varphi _0) a(\varphi _0) U_N^* e^{B} \xi _N \rangle \\&= \frac{1}{N} \langle e^{B} \xi _N, \mathcal{N}_+ e^{B} \xi _N \rangle \le \frac{C}{N} \langle \xi _N , \mathcal{N}_+ \xi _N \rangle \le \frac{C( 1 +K)}{N} \end{aligned} \end{aligned}$$

which concludes the proof of (8). $\square$

## Analysis of the Excitation Hamiltonian $\mathcal{R}_{N}$

In this section, we show Proposition 4, where we establish a lower bound for the operator $\mathcal{R}_{N,\alpha } = e^{-A} \mathcal{G}_{N,\alpha }^\text {eff} e^A$, with $\mathcal{G}^\text {eff}_{N,\alpha }$ as defined in (42) and with

75 $$\begin{aligned} A = \frac{1}{\sqrt{N}} \sum _{r, v \in \varLambda ^*_+} \eta _r \big [b^*_{r+v}a^*_{-r}a_v - \text {h.c.}\big ] \, . \end{aligned}$$

We decompose

76 $$\begin{aligned} \mathcal{G}_{N,\alpha }^\text {eff} = \mathcal{O}_{N} + \mathcal{K}+\mathcal{Z}_N+ \mathcal{C}_{N} + \mathcal{V}_N \end{aligned}$$

with $\mathcal{K}$ and $\mathcal{V}_N$ as in (38), and with

77 $$\begin{aligned} \begin{aligned} \mathcal{O}_{N} =&\;\frac{1}{2} {\widehat{\omega }}_N(0) (N-1) \Big (1-\frac{\mathcal{N}_+}{N}\Big ) + \big [ 2 N{\widehat{V}}(0)- \frac{1}{2} {\widehat{\omega }}_N(0) \big ]\mathcal{N}_+ \Big (1-\frac{\mathcal{N}_+}{N}\Big ) , \\ \mathcal{Z}_N =&\; \frac{1}{2} \sum _{ p\in \varLambda _+^*}{\widehat{\omega }}_N(p)(b_pb_{-p}+\text{ h.c.})\\ \mathcal{C}_{N} =&\; \sqrt{N} \sum _{p,q \in \varLambda ^*_+ : p + q \not = 0} \widehat{V} (p/e^N) \left[ b_{p+q}^* a_{-p}^* a_q + \text{ h.c. }\right] . \end{aligned} \end{aligned}$$

We will analyze the conjugation of all terms on the r.h.s. of (76) in Sects. 6.2–6.6. The estimates emerging from these subsections will then be combined in Sect. 6.6 to conclude the proof of Proposition 4. Throughout the section, we will need Proposition 3 to control the growth of the expectation of the energy $\mathcal{H}_N = \mathcal{K}+ \mathcal{V}_N$ under the action of (75); the proof of Proposition 3 is contained in Sect. 6.1.

In this section, we will always assume that $V \in L^3 ({\mathbb {R}}^2)$ is compactly supported, pointwise non-negative and spherically symmetric.

### A Priori Bounds on the Energy

In this section, we show Proposition 3. To this end, we will need the following proposition.

#### Proposition 7

Let $\mathcal{V}_N$ and *A* be defined in (38) and (44) respectively. Then, there exists a constant $C > 0$ such that

$$\begin{aligned}&[&\mathcal{V}_N,A] = \frac{1}{N^{1/2}}\sum _{\begin{array}{c} u,r,v \in \varLambda _+^*\\ u\ne -v \end{array}} {\widehat{V}}((u-r)/e^N) \eta _r\big [b^*_{u+v}a^*_{-u} a_v +\text{ h.c. }\big ] + \delta _{\mathcal{V}_N} \end{aligned}$$

where

78 $$\begin{aligned} \begin{aligned} | \langle \xi , \delta _{\mathcal{V}_N} \xi \rangle | \le&\; C (\log N)^{1/2} N^{1/2 - \alpha } \Vert \mathcal{H}_N^{1/2} \xi \Vert ^2 \end{aligned} \end{aligned}$$

for any $\alpha > 0$, for all $\xi \in \mathcal{F}^{\le N}_+$, and $N \in {\mathbb {N}}$ large enough.

#### Proof

We proceed as in [4, Prop. 8.1], computing $[ a_{p+u}^* a_{q}^* a_{p}a_{q+u}, b^*_{r+v} a^*_{-r}a_{v}]$. We obtain

$$\begin{aligned}{}[\mathcal{V}_N, A] = \frac{1}{N^{1/2}}\sum ^*_{u\in \varLambda ^*, r, v \in \varLambda ^*_+} \widehat{V}((u-r)/e^N)\eta _r b^*_{u+v} a_{-u}^*a_v + \varTheta _1 + \varTheta _2 + \varTheta _3 + \text{ h.c. }\end{aligned}$$

with

79 $$\begin{aligned} \begin{aligned} \varTheta _1&:=\;\frac{1}{\sqrt{N}}\sum ^*_{\begin{array}{c} u\in \varLambda ^*\\ r,p,v \in \varLambda ^*_+ \end{array}}\widehat{V} (u/e^N) \eta _r b_{p+u}^* a_{r+v-u}^*a_{-r}^*a_{p}a_{v}\,, \\ \varTheta _2&:=\;\frac{1}{\sqrt{N}}\sum ^*_{\begin{array}{c} u\in \varLambda ^*\\ p,r,v\in \varLambda ^*_+ \end{array}}\widehat{V} (u/e^N) \eta _r b_{r+v}^* a_{p+u}^*a_{-r-u}^*a_{p}a_{v} \,, \\ \varTheta _3&:=\;-\frac{1}{\sqrt{N}}\sum ^*_{\begin{array}{c} u\in \varLambda ^*,p,r,v\in \varLambda _+^* \end{array}}\widehat{V} (u/e^N) \eta _r b_{r+v}^* a_{-r}^*a_{p+u}^*a_{p}a_{v+u} \,. \end{aligned} \end{aligned}$$

and with $\sum ^*$ running over all momenta, except choices for which the argument of a creation or annihilation operator vanishes. We conclude that $\delta _{\mathcal{V}_N} = \varTheta _1 + \varTheta _2 + \varTheta _3 + \text{ h.c. }$. Next, we show that each error term $\varTheta _j$, with $j=1,2,3$, satisfies (78). To bound $\varTheta _1$ we switch to position space and apply Cauchy–Schwarz. We find

$$\begin{aligned} \begin{aligned} |\langle \xi , \varTheta _1\xi \rangle |\le&\; \frac{1}{\sqrt{N}} \int _{\varLambda ^2}dxdy\; e^{2N}V(e^N(x-y)) \Vert {\check{a}}(\check{ \eta }_y){\check{a}}_y {\check{a}}_x \xi \Vert \Vert {\check{a}}_y {\check{a}}_x \xi \Vert \\ \le&\; C \Vert \eta \Vert \int _{\varLambda ^2}dx dy \,e^{2N}V(e^N(x-y))\Vert {\check{a}}_y {\check{a}}_x \xi \Vert ^2\\ \le&CN^{-\alpha }\Vert \mathcal{V}_N^{1/2}\xi \Vert ^2\,, \end{aligned} \end{aligned}$$

for any $\xi \in \mathcal{F}_+^{\le N}$ The term $\varTheta _3$ can be controlled similarly. We find

$$\begin{aligned}\begin{aligned} |\langle \xi , \varTheta _3\xi \rangle |=&\; \bigg | \frac{1}{\sqrt{N} } \int _{\varLambda ^2}dxdy \;e^{2N}V(e^N(x-y))\langle \xi , {\check{b}}_x^*{\check{a}}^*({\check{\eta }}_x){\check{a}}^*_y {\check{a}}_x {\check{a}}_y \xi \rangle \bigg | \\&\le C N^{-\alpha }\Vert \mathcal{V}_N^{1/2}\xi \Vert ^2\,. \end{aligned} \end{aligned}$$

It remains to bound the term $\varTheta _2$ on the r.h.s. of (79). Passing to position space we obtain, by Cauchy–Schwarz,

$$\begin{aligned}\begin{aligned} |\langle \xi , \varTheta _2\xi \rangle |=&\; \bigg | \frac{1}{ \sqrt{N} } \int _{\varLambda ^3}dxdydz \;e^{2N}V(e^N(y-z)) {\check{\eta }}(x-z)\langle \xi , {\check{b}}_{x}^*{\check{a}}_y^*{\check{a}}^*_z {\check{a}}_x{\check{a}}_y\xi \rangle \bigg | \\ \le&\; CN^{-1/2} \int _{\varLambda ^3}dxdydz\; e^{2N} V(e^N(y-z)) |{\check{\eta }}(x-z)|\Vert {\check{a}}_x {\check{a}}_y {\check{a}}_z \xi \Vert \Vert {\check{a}}_x {\check{a}}_y \xi \Vert \\ \le&\; CN^{-1/2}\Vert \mathcal{V}_N^{1/2}\mathcal{N}_+^{1/2}\xi \Vert \left[ \int _{\varLambda ^3}dxdydz \, e^{2N}V(e^N(y-z))|{\check{\eta }}(x-z)|^2\Vert {\check{a}}_x {\check{a}}_y \xi \Vert ^2\right] ^{1/2}\,, \end{aligned} \end{aligned}$$

To bound the term in the square bracket, we write it in first quantized form and, for any $2< q < \infty$, we apply Hölder inequality and the Sobolev inequality $\Vert u \Vert _{q} \le C \sqrt{q} \, \Vert u \Vert _{H^1}$ to estimate (denoting by $1< q' < 2$ the dual index to *q*),

80 $$\begin{aligned} \begin{aligned} \sum _{n=2}^N&\sum _{i<j}^n \int \left[ e^{2N} V (e^N \cdot ) * |{\check{\eta }}|^2 \right] (x_i - x_j) \, | \xi ^{(n)} (x_1, \ldots , x_n)|^2 dx_1 \ldots dx_n \\&\le C q \Vert e^{2N} V (e^N \cdot ) * |{\check{\eta }}|^2 \Vert _{q'} \\&\quad \times \sum _{n=2}^N n \sum _{i=1}^n \int \left[ |\nabla _{x_i} \xi ^{(n)} (x_1, \ldots , x_n) |^2 + |\xi ^{(n)} (x_1, \ldots , x_n)|^2 \right] dx_1 \ldots dx_n \\&\le C q \Vert {\check{\eta }} \Vert _{2q'}^2 \Vert (\mathcal{K}+ \mathcal{N}_+)^{1/2} \mathcal{N}_+^{1/2} \xi \Vert ^2. \end{aligned} \end{aligned}$$

With the bounds (25), (26),

$$\begin{aligned} \Vert {\check{\eta }} \Vert _{2q'}^{2} \le \Vert {\check{\eta }} \Vert _2^{2/q'} \Vert {\check{\eta }} \Vert _\infty ^{2(q'-1)/q'} \le N^{-2\alpha /q'} N^{2(q'-1)/q'} \end{aligned}$$

we conclude that

$$\begin{aligned} \begin{aligned} |\langle \xi , \varTheta _2 \xi \rangle |&\le C q^{1/2} N^{-1/2} N^{-\alpha /q'} N^{1/q} \Vert \mathcal{V}_N^{1/2} \mathcal{N}_+^{1/2} \xi \Vert \Vert (\mathcal{K}+ \mathcal{N}_+)^{1/2} \mathcal{N}_+^{1/2} \xi \Vert \\&\le C q^{1/2} N^{1/2} N^{-\alpha /q'} N^{1/q} \Vert \mathcal{V}_N^{1/2} \xi \Vert \Vert \mathcal{K}^{1/2} \xi \Vert \end{aligned} \end{aligned}$$

for any $2< q < \infty$, if $1/q + 1/q' = 1$. Choosing $q = \log N$, we obtain that

$$\begin{aligned} |\langle \xi , \varTheta _2 \xi \rangle | \le C (\log N)^{1/2} N^{1/2 -\alpha } \Vert \mathcal{H}_N^{1/2} \xi \Vert ^2\, . \end{aligned}$$

$$\square $$

Using Proposition 7, we can now show Proposition 3.

#### Proof of Proposition 3

The proof follows a strategy similar to [4, Lemma 8.2]. For fixed $\xi \in \mathcal{F}_+^{\le N}$ and $s\in [0; 1]$, we define

$$\begin{aligned} f_\xi (s) := \langle \xi , e^{-sA} \mathcal{H}_N e^{sA} \xi \rangle \,. \end{aligned}$$

We compute

81 $$\begin{aligned} f'_\xi (s) = \langle \xi , e^{-sA} [\mathcal{K}, A] e^{sA} \xi \rangle + \langle \xi , e^{-sA} [\mathcal{V}_N, A] e^{sA} \xi \rangle \,. \end{aligned}$$

With Proposition 7, we have

$$\begin{aligned}{}[\mathcal{V}_N, A] = \frac{1}{\sqrt{N}} \sum _{\begin{array}{c} u, v\in \varLambda ^*_+, u\ne -v \end{array}} (\widehat{V}(\cdot /e^N)*\eta )(u) \left[ b^*_{u+v} a^*_{-u} a_v + \text{ h.c. }\right] + \delta _{\mathcal{V}_N} \end{aligned}$$

with $\delta _{\mathcal{V}_N}$ satisfying (78). Switching to position space and using Proposition 2 we find , using (25) to bound $\Vert {\check{\eta }} \Vert _\infty \le C N$,

82 $$\begin{aligned} \begin{aligned}&\bigg | \frac{1}{\sqrt{N}}\sum _{\begin{array}{c} u,v\in \varLambda _+^* \end{array}}(\widehat{V}(\cdot /e^N)*\eta )(u) \langle \xi ,e^{-sA}b^*_{u+v}a^*_{-u} a_v e^{sA}\xi \rangle \bigg | \\&= \bigg | \frac{1}{\sqrt{N}}\int _{\varLambda ^2} dx dy\; e^{2N} V(e^N(x-y)){{\check{\eta }}}(x-y) \langle \xi , e^{-sA} {\check{a}}^*_{x} {\check{a}}^*_{y} {\check{a}}_{y} e^{sA}\xi \rangle \bigg |\\&\le N^{1/2} \bigg [ \int _{\varLambda ^2}dxdy\; e^{2N}V(e^N(x-y))\Vert {\check{a}}_x{\check{a}}_ye^{sA}\xi \Vert ^2\bigg ]^{1/2}\\&\quad \times \bigg [ \int _{\varLambda ^2}dxdy\; e^{2N}V(e^N(x-y))\Vert {\check{a}}_ye^{sA}\xi \Vert ^2\bigg ]^{1/2}\\&\le CN^{1/2} \Vert \mathcal{V}_N^{1/2} e^{sA} \xi \Vert \Vert \mathcal{N}_+^{1/2} e^{sA} \xi \Vert \end{aligned} \end{aligned}$$

Together with (78) we conclude that for any $\alpha > 1/2$

83 $$\begin{aligned} \Big | \langle \xi , e^{-sA} [\mathcal{V}_N, A] e^{sA} \xi \rangle \Big | \le C \langle \xi , e^{-sA} \mathcal{H}_N e^{sA} \xi \rangle + CN\langle \xi , e^{-sA} (\mathcal{N}_++1) e^{sA} \xi \rangle \end{aligned}$$

if *N* is large enough. Next, we analyze the first term on the r.h.s. of (81). We compute

84 $$\begin{aligned} \begin{aligned} {[}\mathcal{K},A]&= \frac{1}{\sqrt{N}}\sum _{r, v\in \varLambda ^*_+ } 2r^2\eta _r \big [b^*_{r+v}a^*_{-r} a_v +\text {h.c.}\big ]\\&\quad + \frac{2}{\sqrt{N}}\sum _{r, v\in \varLambda ^*_+ } r\cdot v \;\eta _r\big [b^*_{r+v}a^*_{-r} a_v +\text {h.c.}\big ] \\&=: \text {T}_1 + \text {T}_2 \,. \end{aligned} \end{aligned}$$

With (31), we write

85 $$\begin{aligned} \begin{aligned} \text {T}_1 = \;&-\sqrt{N}\sum _{\begin{array}{c} r,v\in \varLambda _+^*\\ r\ne -v \end{array} } ({\widehat{V}}(\cdot /e^N)*{\widehat{f}}_{N,\ell })(r) \big [b^*_{r+v}a^*_{-r} a_v +\text {h.c.}\big ]\\&+ 2\sqrt{N}\sum _{r, v\in \varLambda ^*_+ } e^{2N} \lambda _\ell ({\widehat{\chi }}_\ell * \widehat{f}_{N,\ell })(r) \big [b^*_{r+v}a^*_{-r} a_v +\text {h.c.}\big ]\\ =:&\; \text {T}_{11} + \text {T}_{12}\,. \end{aligned}\end{aligned}$$

The contribution of $\text {T}_{11}$ can be estimated similarly as in (82); switching to position space and using (20), we obtain

86 $$\begin{aligned} \begin{aligned} \big | \langle \xi _1, \text {T}_{11} \, \xi _2 \rangle \big |&\le C\sqrt{N}\int dxdye^{2N}V(e^N(x-y))f_{\ell }(e^N(x-y))\Vert {\check{a}}_x{\check{a}}_y \xi \Vert \Vert a_y \xi \Vert \\&\le C\sqrt{N}\, \Big [\int dxdye^{2N}V(e^N(x-y)) \Vert {\check{a}}_x{\check{a}}_y \xi \Vert ^2 \Big ]^{1/2}\\&\quad \times \Big [ \int dxdye^{2N}V(e^N(x-y))f_{\ell }(e^N(x-y))\Vert a_y \xi \Vert ^2 \Big ]^{1/2}\\&\le C \Vert \mathcal{V}_N^{1/2} \xi \Vert \Vert \mathcal{N}_+^{1/2} \xi \Vert \,. \end{aligned} \end{aligned}$$

for any $\xi \in \mathcal{F}^{\le N}_+$. The second term in (85) can be controlled using that for any $\xi \in \mathcal{F}^{\le N}_+$ and $2 \le q < \infty$ we have

87 $$\begin{aligned} \begin{aligned}&N^{2\alpha } \int _{\varLambda ^2} dx dy \, \chi (|x-y|\le N^{-\alpha }) \Vert {\check{a}}_x {\check{a}}_y \xi \Vert \Vert {\check{a}}_x \xi \Vert \\&\le N^{2\alpha } \int _{\varLambda ^2} dx \Vert {\check{a}}_x\xi \Vert \left( \int dy \, \chi (|x-y|\le N^{-\alpha })\right) ^{1-1/q}\left( \int dy \Vert {\check{a}}_x{\check{a}}_y\xi \Vert ^q\right) ^{1/q}\\&\le C N^{2\alpha /q}q^{1/2}\left[ \int dx\Vert {\check{a}}_x\xi \Vert ^2 \right] ^{1/2}\left[ \int dxdy \Vert {\check{a}}_x\nabla _y{\check{a}}_y\xi \Vert ^2+\int dxdy\Vert {\check{a}}_x{\check{a}}_y\xi \Vert ^2\right] ^{1/2}\\&\le C N^{2\alpha /q}q^{1/2}\Vert (\mathcal{N}_++1)^{1/2}\xi \Vert \left[ \Vert \mathcal{K}^{1/2}(\mathcal{N}_++1)^{1/2}\xi \Vert +\Vert (\mathcal{N}_++1)\xi \Vert \right] . \end{aligned} \end{aligned}$$

Hence, choosing $$q=\log N$$,

88 $$\begin{aligned} \begin{aligned} \big | \langle \xi ,&\text {T}_{12} \xi \rangle \big | \\&= \Big |\sqrt{N} e^{2N} \lambda _\ell \int _{\varLambda ^2} dx dy \, \chi (|x-y|\le N^{-\alpha })f_{N,\ell }(x-y) {\bigl \langle {\xi , {\check{b}}^*_x{\check{a}}^*_y{\check{a}}_x \xi }\bigr \rangle }\Big |\\&\le C N^{2\alpha -1/2} \int _{\varLambda ^2} dx dy \, \chi (|x-y|\le N^{-\alpha }) \Vert {\check{a}}_x {\check{a}}_y \xi \Vert \Vert {\check{a}}_x \xi \Vert \\&\le C (\log N)^{1/2} \Vert (\mathcal{N}_++1)^{1/2}\xi \Vert \left[ \Vert \mathcal{K}^{1/2}\xi \Vert +\Vert (\mathcal{N}_++1)^{1/2}\xi \Vert \right] \,, \end{aligned} \end{aligned}$$

With (86) and (88) we conclude that

89 $$\begin{aligned} |\langle \xi , e^{-A} \text {T}_1 e^A \xi \rangle | \le C (\log N)^{1/2}\Vert (\mathcal{H}_N + 1)^{1/2} e^{sA}\xi \Vert \Vert (\mathcal{N}_++1)^{1/2} e^{sA} \xi \Vert \,. \end{aligned}$$

for all $\xi \in \mathcal{F}_+^{\le N}$. As for the second term on the r.h.s. of (84) we have

90 $$\begin{aligned} \begin{aligned}&\big | \langle \xi , \text {T}_2 \xi \rangle \big | \\&\quad \le \frac{C}{\sqrt{N}}\bigg [\sum _{r\in \varLambda ^*_+ } |r|^2 \Vert \mathcal{N}_+^{1/2} a_{-r} \xi \Vert ^2 \bigg ]^{1/2}\bigg [ \sum _{r, v\in \varLambda ^*_+ } |v|^{2}\eta _r^2\Vert a_{v} \xi \Vert ^2 \bigg ]^{1/2}\\&\quad \le CN^{-\alpha } \Vert \mathcal{K}^{1/2}\xi \Vert ^2. \end{aligned} \end{aligned}$$

for any $\xi \in \mathcal{F}^{\le N}_+$. With (89) and Proposition 2, we conclude that

$$\begin{aligned} |\langle \xi , e^{-sA} [\mathcal{K}, A] e^{sA} \xi \rangle | \le C \langle \xi , e^{-sA} \mathcal{H}_N e^{sA}\xi \rangle + C \log N \langle \xi , e^{-sA} \mathcal{N}_+ e^{sA} \xi \rangle \,. \end{aligned}$$

Combining with Eq. (83) we obtain

$$\begin{aligned} |\langle \xi , e^{-sA} [\mathcal{H}_N, A] e^{sA} \xi \rangle | \le C \langle \xi , e^{-sA} \mathcal{H}_N e^{sA}\xi \rangle + C N \langle \xi , e^{-sA} \mathcal{N}_+ e^{sA}\xi \rangle \,. \end{aligned}$$

With Proposition 2 we obtain the differential inequality

$$\begin{aligned} | f'_\xi (s) | \le C f_\xi (s) + CN \langle \xi , (\mathcal{N}_+ + 1) \xi \rangle \,. \end{aligned}$$

By Gronwall’s Lemma, we find (45). $\square$

### Analysis of $e^{-A} \mathcal{O}_N e^{A}$

In this section we study the contribution to $\mathcal{R}_{N,\alpha }$ arising from the operator $\mathcal{O}_N$, defined in (77). To this end, it is convenient to use the following lemma.

#### Lemma 3

Let *A* be defined in (44). Then, there exists a constant $C > 0$ such that

$$\begin{aligned} \sum _{p\in \varLambda _+^*} F_p\, e^{-A} a^*_p a_p e^{A} = \sum _{p\in \varLambda _+^*} F_p\, a^*_p a_p + \mathcal{E}_F \end{aligned}$$

where

$$\begin{aligned} | {\bigl \langle {\xi _1, \mathcal{E}_F \xi _2 }\bigr \rangle }|\le CN^{-\alpha } \Vert F\Vert _\infty \Vert (\mathcal{N}_++1)^{1/2}\xi _1\Vert \Vert (\mathcal{N}_++1)^{1/2}\xi _2\Vert \end{aligned}$$

for all $\alpha > 0$, $\xi _1, \xi _2 \in \mathcal{F}_+^{\le N}$, $F \in \ell ^{\infty } (\varLambda ^*_+)$, and $N \in {\mathbb {N}}$ large enough.

#### Proof

The lemma is analogous to [4, Lemma 8.6]. We estimate

$$\begin{aligned}\begin{aligned} \Big | \sum _{p\in \varLambda _+^*} F_p&( \langle \xi _1, e^{-A} a^*_p a_p e^{A} \xi _2 \rangle - \langle \xi _1 , a^*_p a_p \xi _2 \rangle ) \Big | \\&= \Big | \int _0^1ds\;\sum _{p\in \varLambda _+^*} F_p \langle \xi _1 , e^{-sA}[a^*_p a_p, A] e^{sA} \xi _2 \rangle \Big | \\&\le \frac{1}{\sqrt{N}} \int _0^1 ds \sum _{r, v\in \varLambda ^*_+ } | F_{r+v}+F_{-r} - F_v | |\eta _r| |\langle e^{sA} \xi _1, b^*_{r+v}a^*_{-r}a_v e^{sA} \xi _2 \rangle | \\&\le C \Vert \eta \Vert \Vert F \Vert _\infty \Vert (\mathcal{N}_++1)^{1/2}\xi _1\Vert \Vert (\mathcal{N}_++1)^{1/2}\xi _2\Vert \,. \end{aligned} \end{aligned}$$

where we used Proposition 2. $\square$

We consider now the action of $e^A$ on the operator $\mathcal{O}_N$, as defined in (77).

#### Proposition 8

Let *A* be defined in (44). Then there exists a constant $C>0$ such that

$$\begin{aligned} \begin{aligned} e^{-A} \mathcal{O}_N e^A =&\; \frac{1}{2} {\widehat{\omega }}_N(0)(N-1) \left( 1-\frac{\mathcal{N}_+}{N}\right) + \big [2N{\widehat{V}}(0)- \frac{1}{2} {\widehat{\omega }}_N(0) \big ] \mathcal{N}_+ (1-\mathcal{N}_+/N) \\ {}&+ \delta _{\mathcal{O}_N} \end{aligned} \end{aligned}$$

where

$$\begin{aligned} \pm \delta _{\mathcal{O}_N} \le CN^{1-\alpha } (\mathcal{N}_+ +1) \end{aligned}$$

for all $\alpha > 0$, and $N \in {\mathbb {N}}$ large enough.

#### Proof

The proof is very similar to [4, Prop. 8.7]. First of all, with Lemma 3 we can bound

$$\begin{aligned}\begin{aligned} \pm \bigg \{ e^{-A}&\left[ \frac{1}{2} {\widehat{\omega }}_N(0) (N-1)\left( 1-\frac{\mathcal{N}_+}{N}\right) + \big [2N{\widehat{V}}(0)- \frac{1}{2} {\widehat{\omega }}_N(0)\big ]\mathcal{N}_+ \right] e^{A} \\&\quad - \left[ \frac{1}{2} {\widehat{\omega }}_N(0) (N-1)\left( 1-\frac{\mathcal{N}_+}{N}\right) + \big [2 N {\widehat{V}}(0)- \frac{1}{2} {\widehat{\omega }}_N(0) \big ]\mathcal{N}_+ \right] \bigg \}\\&\quad \le C N^{1-\alpha }(\mathcal{N}_++1)\,. \end{aligned} \end{aligned}$$

Moreover, for the contribution quadratic in $\mathcal{N}_+$, we can decompose

$$\begin{aligned} \begin{aligned}&\left\langle \xi , \left[ e^{-A} \mathcal{N}_+^2 e^A - \mathcal{N}_+^2 \right] \xi \right\rangle \\&\quad = \left\langle \xi _1, \left[ e^{-A} \mathcal{N}_+ e^A - \mathcal{N}_+ \right] \xi \right\rangle + \left\langle \xi , \left[ e^{-A} \mathcal{N}_+ e^A - \mathcal{N}_+ \right] \xi _2 \right\rangle \end{aligned} \end{aligned}$$

with $\xi _1 = e^{-A} \mathcal{N}_+ e^A \xi$ and $\xi _2 = \mathcal{N}_+ \xi$, and estimate, again with Lemma 3,

$$\begin{aligned}\begin{aligned}&\left| \left\langle \xi , \left[ e^{-A} \mathcal{N}_+^2 e^A - \mathcal{N}_+^2 \right] \xi \right\rangle \right| \\&\quad \le C N^{-\alpha } \Vert (\mathcal{N}_+ + 1)^{1/2} \xi \Vert \left[ \Vert (\mathcal{N}_+ + 1)^{1/2} \xi _1 \Vert + \Vert (\mathcal{N}_+ + 1)^{1/2} \xi _2 \Vert \right] \,. \end{aligned} \end{aligned}$$

With Proposition 2, we have $\Vert (\mathcal{N}_+ + 1)^{1/2} \xi _1 \Vert \le C \Vert (\mathcal{N}_+ +1)^{3/2} \xi \Vert$. $\square$

### Contributions from $e^{-A} \mathcal{K}e^{A}$

In Sect. 6.6 we will analyse the contributions to $\mathcal{R}_{N,\alpha }$ arising from conjugation of the kinetic energy operator $\mathcal{K}= \sum _{p \in \varLambda _+^*} p^2 a_p^* a_p$. To this aim we will exploit properties of the commutator $[\mathcal{K}, A]$, collected in the following proposition.

#### Proposition 9

Let *A* be defined as in (44) and ${\widehat{\omega }}_N(r)$ be defined in (39). Then there exists a constant $C>0$ such that

$$\begin{aligned} \begin{aligned} {[}\mathcal{K}, A] =&\; -\sqrt{N}\sum _{p,q\in \varLambda _+^*, p \ne -q } ({\widehat{V}}(\cdot /e^N)*{\widehat{f}}_{N,\ell })(p) ( b^*_{p+q}a^*_{-p} a_q+ \text{ h.c.})\\&\;+ \frac{1}{\sqrt{N}} \sum _{p, q \in \varLambda ^*_+, p \ne -q } {\widehat{\omega }}_N(p) \big [ b^*_{p+q}a^*_{-p} a_q + \text{ h.c. }\big ] + \delta _\mathcal{K}\end{aligned} \end{aligned}$$

where

91 $$\begin{aligned} \big | \langle \xi , \delta _\mathcal{K}\xi \rangle \big | \le C N^{-1} (\log N)^{1/2} \Vert \mathcal{K}^{1/2}\xi \Vert \Vert \mathcal{N}_+^{1/2}\xi \Vert + C N^{-\alpha } \Vert \mathcal{K}^{1/2}\xi \Vert ^2 \end{aligned}$$

for all $\alpha >1$, $\xi \in \mathcal{F}_+^{\le N}$, and $N \in {\mathbb {N}}$ large enough. Moreover, the operator

$$\begin{aligned} \varDelta _\mathcal{K}= \frac{1}{\sqrt{N}} \sum _{p, q \in \varLambda ^*_+, p \ne -q } {\widehat{\omega }}_N(p) \big [ b^*_{p+q}a^*_{-p} a_q , A \big ] \end{aligned}$$

satisfies

92 $$\begin{aligned} \big | \langle \xi , \varDelta _\mathcal{K}\xi \rangle \big | \le C N^{-\alpha } (\log N)^{1/2} \Vert \mathcal{K}^{1/2}\xi \Vert ^2 + C N^{-1} \Vert (\mathcal{N}_+ + 1)^{1/2} \xi \Vert ^2 \end{aligned}$$

for all $\alpha > 1$, $\xi \in \mathcal{F}_+^{\le N}$, and $N \in {\mathbb {N}}$ large enough.

#### Proof

To show (91) we recall from Eqs. (84), (85) that

$$\begin{aligned} \begin{aligned} {[}\mathcal{K},A] =\;&-\sqrt{N}\sum _{\begin{array}{c} r,v\in \varLambda _+^*\\ r\ne -v \end{array} } ({\widehat{V}}(\cdot /e^N)*{\widehat{f}}_{N,\ell })(r) \big [b^*_{r+v}a^*_{-r} a_v +\text {h.c.}\big ]\\&+ 2\sqrt{N}\sum _{r, v\in \varLambda ^*_+ } e^{2N} \lambda _\ell ({\widehat{\chi }}_\ell * \widehat{f}_{N,\ell })(r) \big [b^*_{r+v}a^*_{-r} a_v +\text {h.c.}\big ]\\&\quad + \frac{2}{\sqrt{N}}\sum _{r, v\in \varLambda ^*_+ } r\cdot v \;\eta _r\big [b^*_{r+v}a^*_{-r} a_v +\text {h.c.}\big ] \\ =\;&\text {T}_{11} + \text {T}_{12}+ \text {T}_2 \,. \end{aligned} \end{aligned}$$

with $\text {T}_2$ satisfying (90). Using the definition ${\widehat{\omega }}_{N}(p)=2 N e^{2N} \lambda _\ell \widehat{\chi }_\ell (p)$ we write

$$\begin{aligned}\begin{aligned} \text {T}_{12} =&\frac{1}{\sqrt{N}}\sum _{p, q \in \varLambda ^*_+, p \ne -q } {\widehat{\omega }}_N(p) \big [ b^*_{p+q}a^*_{-p} a_q + \text{ h.c. }\big ] \\&+\frac{2}{\sqrt{N}} \,e^{2N} \lambda _\ell \sum _{p, q \in \varLambda ^*_+, p \ne -q } (\widehat{\chi _\ell }*\eta )(p) \big [ b^*_{p+q}a^*_{-p} a_q + \text{ h.c. }\big ] \\ =&\text {T}_{121}+\text {T}_{122}. \end{aligned}\end{aligned}$$

Hence, $\delta _K = T_2 + T_{122}$. To bound $T_{122}$ we switch to position space:

$$\begin{aligned} \begin{aligned}&| \langle \xi , \text {T}_{122} \xi \rangle | \\&\le CN^{2\alpha -3/2} \int _{\varLambda ^2} \chi _\ell (x-y) {\check{\eta }} (x-y) \Vert {\check{a}}_x{\check{a}}_y\xi \Vert \Vert {\check{a}}_x\xi \Vert \\&\le CN^{2\alpha -3/2} \left[ \int _{\varLambda ^2} \chi _\ell (x-y) \Vert {\check{a}}_x{\check{a}}_y\xi \Vert ^2 dx dy \right] ^{1/2} \left[ \int _{\varLambda ^2} |{\check{\eta }} (x-y)|^2 \Vert {\check{a}}_x \xi \Vert ^2 dx dy \right] ^{1/2} \\&\le CN^{\alpha -3/2} \Vert \mathcal{N}^{1/2}_+ \xi \Vert \left[ \int _{\varLambda ^2} \chi _\ell (x-y) \Vert {\check{a}}_x{\check{a}}_y\xi \Vert ^2 dx dy \right] ^{1/2}. \end{aligned} \end{aligned}$$

To bound the term in the parenthesis, we proceed similarly as in (80). We find

$$\begin{aligned} \int _{\varLambda ^2} \chi _\ell (x-y) \Vert {\check{a}}_x{\check{a}}_y\xi \Vert ^2 dx dy \le C q \Vert \chi _\ell \Vert _{q'} \Vert \mathcal{K}^{1/2} \mathcal{N}_+^{1/2} \xi \Vert ^2 \le C q N^{1-2\alpha /q'} \Vert \mathcal{K}^{1/2} \xi \Vert ^2 \end{aligned}$$

for any $q > 2$ and $1< q' < 2$ with $1/q+ 1/q' =1$. Choosing $q = \log N$, we obtain

$$\begin{aligned} |\langle \xi , \text {T}_{122} \xi \rangle | \le C N^{-1} (\log N)^{1/2} \Vert \mathcal{N}^{1/2}_+ \xi \Vert \Vert \mathcal{K}^{1/2} \xi \Vert \end{aligned}$$

With (90), this implies (91).

Let us now focus on (92). We have

$$\begin{aligned} \begin{aligned} \frac{1}{\sqrt{N}}&\sum _{p, q \in \varLambda ^*_+, p \ne -q } {\widehat{\omega }}_N(p) \big [ b^*_{p+q}a^*_{-p} a_q , A \big ]\\&= \frac{1}{N} \sum _{\begin{array}{c} r, p,q, v\in \varLambda ^*_+, \\ p \ne -q, r\ne -v \end{array}} {\widehat{\omega }}_N(p) \eta _r \big [b^*_{p+q}a^*_{-p} a_q , b^*_{r+v}a^*_{-r} a_v - a_v^* a_{-r}b_{r+v} \big ]\,. \end{aligned} \end{aligned}$$

With the commutators from the proof of Proposition 8.8 in [4], we arrive at

$$\begin{aligned} \frac{1}{\sqrt{N}} \sum _{p, q \in \varLambda ^*_+, p \ne -q } {\widehat{\omega }}_N(p) \big [ b^*_{p+q}a^*_{-p} a_q , A \big ]+ \text {h.c.} = \sum _{j=1}^{12} \varUpsilon _j +\text{ h.c. }\end{aligned}$$

where

93 $$\begin{aligned} \begin{aligned} \varUpsilon _1:=&\; - \frac{1}{N} \sum _{\begin{array}{c} q,r,v \in \varLambda ^*_+,\\ q\ne v, r \ne -v \end{array}} \big ( {\widehat{\omega }}_N(v-q) + {\widehat{\omega }}_N(v) \big ) \eta _r b^*_{r+v}b^*_{-r} a^*_{q-v} a_q \,,\\ \varUpsilon _2:=&\; \frac{1}{N} \sum _{\begin{array}{c} q,r,v \in \varLambda ^*_+,\\ r\ne -v, r \ne -q \end{array} } {\widehat{\omega }}_N(r+q)\eta _r (1-\mathcal{N}_+/N)a^*_{v}a^*_{r+q} a_q a_{r+v}\,,\\ \varUpsilon _3:=&\; \frac{1}{N} \sum _{\begin{array}{c} r,v \in \varLambda ^*_+\,,\\ r \ne -v \end{array}} \big ( {\widehat{\omega }}_N(r+v)+ {\widehat{\omega }}_N(r) \big )\eta _r(1-\mathcal{N}_+/N)a^*_{v} a_v,\\ \varUpsilon _4:=&\;\frac{1}{N} \sum _{\begin{array}{c} q,r,v \in \varLambda ^*_+\,,\\ q\ne v, r \ne -v \end{array} } {\widehat{\omega }}_N(r+v-q)\eta _r(1-\mathcal{N}_+/N)a_v^* a^*_{q-r-v} a_{-r} a_q,\\ \varUpsilon _5:=&\; - \frac{1}{N^{2}} \sum _{\begin{array}{c} p,q,r,v \in \varLambda ^*_+,\\ p\ne -q, r \ne -v \end{array}} {\widehat{\omega }}_N(p) \eta _ra_v^* a^*_{p+q}a^*_{-p}a_{-r} a_{r+v} a_q\,,\\ \varUpsilon _6:=&\; - \frac{1}{N^2}\sum _{\begin{array}{c} q,r,v \in \varLambda ^*_+,\\ q\ne r+v \end{array}} {\widehat{\omega }}_N(r+v)\eta _r a_v^*a^*_{q-r-v}a_{-r} a_q, \\ \varUpsilon _7:=&\; - \frac{1}{N^2} \sum _{\begin{array}{c} q,r,v \in \varLambda ^*_+,\\ q\ne -r, r \ne -v \end{array}} {\widehat{\omega }}_N(r)\eta _r a_v^*a^*_{q+r}a_{r+v}a_q\,,\\ \varUpsilon _8:=&\; \frac{1}{N} \sum _{\begin{array}{c} r,v,p \in \varLambda ^*_+,\\ p \ne -r-v \end{array} } {\widehat{\omega }}_N(p)\eta _r b^*_{p+r+v}b^*_{-p} a^*_{-r}a_{v} \,,\\ \varUpsilon _{9}:=&\; \frac{1}{N} \sum _{\begin{array}{c} p,r,v \in \varLambda ^*_+,\\ p\ne r, r\ne -v \end{array} } {\widehat{\omega }}_N(p)\eta _r b^*_{p-r}b^*_{r+v} a^*_{-p}a_{v} \,,\\ \varUpsilon _{10}:=&\; \frac{1}{N} \sum _{\begin{array}{c} q,r,v \in \varLambda ^*_+,\\ q\ne -r, r\ne -v \end{array} } {\widehat{\omega }}_N(r)\eta _r b^*_{q+r}a^*_{v} a_{q}b_{r+v} \,,\\ \varUpsilon _{11}:=&\; -\frac{1}{N} \sum _{\begin{array}{c} p,r,v \in \varLambda ^*_+,\\ p\ne -v,r\ne -v \end{array} } {\widehat{\omega }}_N(p)\eta _r b^*_{p+v}a^*_{-p} a_{-r}b_{r+v} \,,\\ \varUpsilon _{12}:=&\; \frac{1}{N} \sum _{\begin{array}{c} q, r,v \in \varLambda ^*_+\\ r \ne q-v, -v \end{array} } {\widehat{\omega }}_N(r+v)\eta _r b^*_{q-r-v}a^*_{v} a_{-r}b_{q} \,.\\ \end{aligned} \end{aligned}$$

To conclude the proof of Proposition 9, we show that all operators in (93) satisfy (92). To study all these terms it is convenient to switch to position space. We recall that ${{\widehat{\omega }}}_N(p)= g_N {{\widehat{\chi }}}(\ell p)$ with $|g_N|\le C$ and $\ell = N^{-\alpha }$. Using (87) we find:

$$\begin{aligned}\begin{aligned} \big | \langle \xi , \varUpsilon _1 \xi \rangle \big |&\le C N^{2\alpha -1} \int _{\varLambda ^2}dx dy \, \chi _\ell (x-y)\Vert {\check{b}}(\check{ \eta }_x){\check{b}}_x{\check{a}}_y\xi \Vert \left[ \Vert {\check{a}}_x \xi \Vert + \Vert {\check{a}}_y\xi \Vert \right] \\&\le CN^{2\alpha -1}\Vert \eta \Vert \int _{\varLambda ^2}dx dy \, \chi _\ell (x-y)\Vert {\check{b}}_x{\check{a}}_y(\mathcal{N}_++1)^{1/2} \xi \Vert \Vert {\check{a}}_x\xi \Vert \\&\le C N^{-\alpha } (\log N)^{1/2} \Vert (\mathcal{N}_++1)^{1/2}\xi \Vert \Vert \mathcal{K}^{1/2}\xi \Vert \,. \end{aligned}\end{aligned}$$

The expectation of $\varUpsilon _2$ is bounded following the same strategy used to show (87). For any $2\le q < \infty$ we have

$$\begin{aligned}\begin{aligned}&\big | \langle \xi , \varUpsilon _2 \xi \rangle \big | \\&\le CN^{2\alpha -1}\int _{\varLambda ^3}dxdydz \chi _\ell (z-y)|{\check{\eta }}(z-x)| \Vert {\check{a}}_x{\check{a}}_y\xi \Vert \Vert {\check{a}}_z{\check{a}}_x\xi \Vert \\&\le CN^{2\alpha -1}\int _{\varLambda ^2}dxdz |{\check{\eta }}(z-x)|\Vert {\check{a}}_z{\check{a}}_x\xi \Vert \\&\quad \times \left( \int _{\varLambda }dy \, \chi (|z-y|\le N^{-\alpha })\right) ^{1-1/q} \left( \int _{\varLambda }dy\Vert {\check{a}}_x{\check{a}}_y\xi \Vert ^q\right) ^{1/q}\\&\le Cq^{1/2}N^{2\alpha /q-1}\Vert \eta \Vert \Vert (\mathcal{N}_++1)\xi \Vert \left[ \int _{\varLambda ^2} dxdy\Vert {\check{a}}_x\nabla _y{\check{a}}_y\xi \Vert ^2+\int _{\varLambda ^2} dxdy\Vert {\check{a}}_x{\check{a}}_y\xi \Vert ^2\right] ^{1/2}\\&\le CN^{-\alpha }(\log N)^{1/2}\Vert (\mathcal{N}_++1)^{1/2}\xi \Vert \Vert \mathcal{K}^{1/2}\xi \Vert \,, \end{aligned}\end{aligned}$$

where in the last line we chose $q=\log N$. The term $\varUpsilon _3$ is of lower order; using that $\big | \sum _r {\widehat{\omega }}_N (r) \eta _r \big | \le \Vert {\widehat{\chi }} (./N^\alpha ) \Vert _2 \Vert \eta \Vert _2 \le C$ and Cauchy–Schwarz, we easily obtain

$$\begin{aligned} \big | \langle \xi , \varUpsilon _{3} \xi \rangle \big | \le CN^{-1}\Vert (\mathcal{N}_++1)^{1/2}\xi \Vert ^2\,. \end{aligned}$$

The term $\varUpsilon _4$ can be estimated as $\varUpsilon _1$ using (87):

$$\begin{aligned}\begin{aligned} \big | \langle \xi , \varUpsilon _4 \xi \rangle \big |&\le CN^{2\alpha -1}\int _{\varLambda ^2}dxdy \, \chi _\ell (x-y)\Vert {\check{a}}_x{\check{a}}_y\xi \Vert \Vert {\check{a}}({\check{\eta }}_y){\check{a}}_y\xi \Vert \\&\le CN^{2\alpha -1}\Vert \eta \Vert \int _{\varLambda ^2}dxdy \,\chi _\ell (x-y)\Vert {\check{a}}_x{\check{a}}_y\xi \Vert \Vert {\check{a}}_y (\mathcal{N}_++1)^{1/2}\xi \Vert \\&\le CN^{-\alpha } (\log N)^{1/2}\Vert (\mathcal{N}_++1)^{1/2} \xi \Vert \Vert \mathcal{K}^{1/2}\xi \Vert \,. \end{aligned}\end{aligned}$$

The term $\varUpsilon _5$ is bounded similarly to $\varUpsilon _2$; with $q=\log N$ we have

$$\begin{aligned}\begin{aligned} \big |\langle \xi , \varUpsilon _5 \xi \rangle \big |&\le CN^{2\alpha -2}\Vert \eta \Vert \int _{\varLambda ^3} dxdydz \, \chi _\ell (y-z)\Vert {\check{a}}_x{\check{a}}_y{\check{a}}_z\xi \Vert \Vert \mathcal{N}_+^{1/2}{\check{a}}_x{\check{a}}_y\xi \Vert \\&\le CN^{2\alpha -3/2}\Vert \eta \Vert \int _{\varLambda ^2} dxdy \, \Vert {\check{a}}_x{\check{a}}_y\xi \Vert \\&\quad \times \left( \int _{\varLambda } dz \, \chi (|y-z|\le N^{-\alpha })\right) ^{1-1/q}\left( \int _{\varLambda } dz \, \Vert {\check{a}}_x{\check{a}}_y{\check{a}}_z\xi \Vert ^q\right) ^{1/q}\\&\le CN^{-\alpha } (\log N)^{1/2}\Vert (\mathcal{N}_++1)^{1/2}\xi \Vert \Vert \mathcal{K}^{1/2}\xi \Vert \,. \end{aligned}\end{aligned}$$

The terms $\varUpsilon _6$ and $\varUpsilon _7$ are of smaller order and can be bounded with Cauchy–Schwarz; we have

$$\begin{aligned}\begin{aligned} \big |\langle \xi , \varUpsilon _6 \xi \rangle \big |&\le CN^{2\alpha -2} \int _{\varLambda ^2} dxdydz \,\chi _\ell (x-y)\Vert {\check{a}}_x{\check{a}}_y\xi \Vert \Vert {\check{a}}({\check{\eta }}_x){\check{a}}_y\xi \Vert \\&\le CN^{\alpha -3/2}\left( \int _{\varLambda ^2} dxdy \, \Vert {\check{a}}_x{\check{a}}_y\xi \Vert ^2\right) ^{1/2}\left( \int _{\varLambda ^2} dxdy \, \chi (|x-y|\le N^{-\alpha }) \Vert {\check{a}}_y\xi \Vert ^2\right) ^{1/2}\\&\le CN^{-1}\Vert (\mathcal{N}_++1)^{1/2}\xi \Vert ^2, \end{aligned}\end{aligned}$$

and

$$\begin{aligned}\begin{aligned} \big |\langle \xi , \varUpsilon _7 \xi \rangle \big |&\le CN^{2\alpha -2}\int _{\varLambda ^3} dxdydz \, \chi _\ell (y-z)|{\check{\eta }}(z-x)|\Vert {\check{a}}_x{\check{a}}_y\xi \Vert ^2\\&\le CN^{2\alpha -2}\left( \int _{\varLambda ^3} dxdydz \, \chi _\ell (y-z) \Vert {\check{a}}_x{\check{a}}_y\xi \Vert ^2\right) ^{1/2} \\&\quad \times \left( \int _{\varLambda ^3} dxdydz \, |{\check{\eta }}(z-x)|^2 \Vert {\check{a}}_x{\check{a}}_y\xi \Vert ^2\right) ^{1/2}\\&\le CN^{-1}\Vert (\mathcal{N}_++1)^{1/2}\xi \Vert ^2\,. \end{aligned}\end{aligned}$$

The terms $\varUpsilon _8, \varUpsilon _{11}, \varUpsilon _{12}$ are again bounded, as $\varUpsilon _1$, using (87). We find

$$\begin{aligned}\begin{aligned} \big |\langle \xi , \big (\varUpsilon _8 + \varUpsilon _{11} + \varUpsilon _{12} \big ) \xi \rangle \big |&\le CN^{2\alpha -1}\Vert \eta \Vert \int _{\varLambda ^2} dxdy \, \chi _\ell (x-y)\Vert \mathcal{N}_+^{1/2}{\check{a}}_x{\check{a}}_y\xi \Vert \Vert {\check{a}}_x\xi \Vert \\&\le CN^{-\alpha } (\log N)^{1/2}\Vert (\mathcal{N}_++1)^{1/2}\xi \Vert \Vert \mathcal{K}^{1/2}\xi \Vert \,. \end{aligned}\end{aligned}$$

It remains to bound $\varUpsilon _9$ and $\varUpsilon _{10}$. The term $\varUpsilon _9$ is bounded analogously to $\varUpsilon _2$:

$$\begin{aligned}\begin{aligned}&\big |\langle \xi , \varUpsilon _{9} \xi \rangle \big | \\&\; \le CN^{2\alpha -1}\int _{\varLambda ^3} dxdydz \, \chi _\ell (x-z)|{\check{\eta }}(x-y)|\Vert {\check{a}}_x{\check{a}}_y{\check{a}}_z\xi \Vert \Vert {\check{a}}_y\xi \Vert \\&\;\le CN^{2\alpha -1}\int _{\varLambda ^2} dxdy \, |{\check{\eta }}(x-y)|\Vert {\check{a}}_y\xi \Vert \left( \int _{\varLambda } dz \, \chi (|y-z|\le N^{-\alpha })\right) ^{1-1/q}\\&\quad \times \left( \int _{\varLambda } dz \, \Vert {\check{a}}_x{\check{a}}_y{\check{a}}_z\xi \Vert ^q\right) ^{1/q}\\&\;\le Cq^{1/2}N^{2\alpha /q-1}\left[ \int _{\varLambda ^2}dxdy \, |{\check{\eta }}(x-y)|^2 \Vert {\check{a}}_y\xi \Vert ^2\right] ^{1/2}\left[ \int _{\varLambda ^3}dxdy \, \Big \Vert \Vert {\check{a}}_x{\check{a}}_y{\check{a}}_z \xi \Vert \Big \Vert _{L^q_z}^2\right] ^{1/2}\\&\;\le CN^{-\alpha }(\log N)^{1/2}\Vert (\mathcal{N}_++1)^{1/2}\xi \Vert \Vert \mathcal{K}^{1/2}\xi \Vert \,. \end{aligned}\end{aligned}$$

As for $\varUpsilon _{10}$, we find

$$\begin{aligned} \big |\langle \xi , \varUpsilon _{10} \xi \rangle \big | \le CN^{2\alpha -1}\int _{\varLambda ^3} dx dy dz \, \chi _\ell (y-z) |{\check{\eta }}(x-z)| \Vert {\check{a}}_x{\check{a}}_y\xi \Vert ^2\end{aligned}$$

Proceeding as in (80), we obtain

$$\begin{aligned} \big |\langle \xi , \varUpsilon _{10} \xi \rangle \big | \le C q N^{2\alpha } \Vert \chi _\ell * |{\check{\eta }}| \Vert _{q'} \Vert \mathcal{K}^{1/2} \xi \Vert ^2 \le C q \Vert {\check{\eta }} \Vert _{q'} \Vert \mathcal{K}^{1/2} \xi \Vert ^2 \end{aligned}$$

for any $q > 2$, and $q' < 2$ with $1/q + 1/q' = 1$. Since, for an arbitrary $q' < 2$, $\Vert {\check{\eta }} \Vert _{q'} \le \Vert {\check{\eta }} \Vert _2 = \Vert \eta \Vert _2 \le N^{-\alpha }$, we obtain

$$\begin{aligned} \big | \langle \xi , \varUpsilon _{10} \xi \rangle \big | \le C N^{-\alpha } \Vert \mathcal{K}^{1/2} \xi \Vert ^2 \end{aligned}$$

We conclude that for any $\alpha >1$

$$\begin{aligned} \left| \langle \xi , \sum _{j=1}^{12}\varUpsilon _{i} \xi \rangle \right| \le C N^{-\alpha } (\log N )^{1/2} \, \Vert (\mathcal{K}+1)^{1/2}\xi \Vert ^2 + C N^{-1} \Vert (\mathcal{N}_+ + 1)^{1/2} \xi \Vert ^2 \,. \end{aligned}$$

$$\square $$

### Analysis of $e^{-A} \mathcal{Z}_N e^{A}$

In this subsection, we consider contributions to $\mathcal{R}_{N,\alpha }$ arising from conjugation of $\mathcal{Z}_{N}$, as defined in (77).

#### Proposition 10

Let *A* be defined in (44). Then, there exists a constant $C>0$ such that

$$\begin{aligned} e^{A} \mathcal{Z}_N e^{-A} =\frac{1}{2} \sum _{\begin{array}{c} p \in \varLambda ^*_+ \end{array}} {\widehat{\omega }}_N(p)\; \big ( b^*_p b^*_{-p} + b_p b_{-p} \big ) +\delta _{\mathcal{Z}_N} \end{aligned}$$

where

$$\begin{aligned} \pm \delta _{\mathcal{Z}_{N}} \le C N^{1-\alpha }(\mathcal{H}_N+1) \end{aligned}$$

for all $\alpha > 0$, and $N \in {\mathbb {N}}$ large enough.

#### Proof

We have

94 $$\begin{aligned} \begin{aligned} \frac{1}{2} \sum _{\begin{array}{c} p \in \varLambda ^*_+ \end{array}} {\widehat{\omega }}_N (p)&\; \big [e^{-A}\big ( b^*_p b^*_{-p} + b_p b_{-p} \big )e^{A}-\big ( b^*_p b^*_{-p} + b_p b_{-p} \big ) \big ]\\&= \frac{1}{2} \int _0^1ds\sum _{\begin{array}{c} p \in \varLambda ^*_+ \end{array}} {\widehat{\omega }}_N (p) \; e^{-sA}\big [ b^*_p b^*_{-p} + b_p b_{-p},A \big ]e^{sA}. \end{aligned} \end{aligned}$$

We compute

95 $$\begin{aligned} \begin{aligned} \frac{1}{2} \sum _{\begin{array}{c} p \in \varLambda ^*_+ \end{array}}&{{\widehat{\omega }}}_N(p) \big [ b^*_p b^*_{-p} , b^*_{r+v} a^*_{-r}a_v - a^*_v a_{-r} b_{r+v} \big ] \\ =\;&- {{\widehat{\omega }}}_N(v) b^*_{r+v}b^*_{-v}b^*_{-r} + {{\widehat{\omega }}}_N(r) b^*_{v} \Big ( b^*_{r}b_{r+v}-\frac{2}{N} a^*_r a_{r+v}\Big )\\&+{{\widehat{\omega }}}_N(r+v) \Bigg (1-\frac{\mathcal{N}_+}{N} \Bigg )b^*_{-r-v}a^*_va_{-r} -\frac{1}{N} \sum _{\begin{array}{c} p \in \varLambda ^* \end{array}} {{\widehat{\omega }}}_N(p) b^*_{p}a^*_{-p}a^*_{v}a_{-r}a_{r+v}. \end{aligned} \end{aligned}$$

With (95) we write

$$\begin{aligned} \frac{1}{2} \sum _{\begin{array}{c} p\in \varLambda ^* \end{array}} {\widehat{\omega }}_N (p) \big [ b^*_p b^*_{-p} + b_p b_{-p} ,A \big ]= \sum _{j=1}^4 \varPi _j + \text{ h.c. }\end{aligned}$$

with

$$\begin{aligned} \begin{aligned} \varPi _1 =\;&- \frac{1}{\sqrt{N}}\sum _{\begin{array}{c} r,v \in \varLambda ^*_+\\ r\ne -v \end{array}} {\widehat{\omega }}_N (v)\eta _{r} b^*_{r+v}b^*_{-v}b^*_{-r}\,,\\ \varPi _2 =\;&\frac{1}{\sqrt{N}} \sum _{\begin{array}{c} r,v \in \varLambda ^*_+: \\ r \ne -v \end{array}} {\widehat{\omega }}_N(r)\eta _{r} b^*_{v}\Big ( b^*_{r}b_{r+v}-\frac{2}{N} a^*_r a_{r+v}\Big )\,, \\ \varPi _3 =\;&\frac{1}{\sqrt{N}}\sum _{\begin{array}{c} r,v \in \varLambda ^*_+ \\ r \ne -v \end{array}} {\widehat{\omega }}_N (r+v) \eta _{r} \Bigg (1-\frac{\mathcal{N}_+}{N} \Bigg )b^*_{-r-v}a^*_va_{-r}\,, \\ \varPi _4 =\;&- \frac{1}{N^{3/2}}\sum _{\begin{array}{c} r,v,p \in \varLambda ^*_+: \\ r \ne -v \end{array}} {\widehat{\omega }}_N (p)\eta _{r} b^*_{p}a^*_{-p}a^*_{v}a_{-r}a_{r+v} \,.\\ \end{aligned} \end{aligned}$$

To bound the first term, we observe, with (52),

$$\begin{aligned} \begin{aligned} |\langle \xi , \varPi _1 \xi \rangle |&\le \frac{\Vert \eta \Vert }{\sqrt{N}} \Vert \mathcal{K}^{1/2} \mathcal{N}_+^{1/2} \xi \Vert \Vert (\mathcal{N}_+ + 1)^{1/2} \xi \Vert \left[ \sum _{v \in \varLambda ^*_+} \frac{|{\widehat{\omega }}_N (v)|^2}{v^2} \right] ^{1/2} \\&\le C N^{-\alpha } (\log N)^{1/2} \Vert \mathcal{K}^{1/2} \xi \Vert \Vert (\mathcal{N}_+ + 1)^{1/2} \xi \Vert \,. \end{aligned} \end{aligned}$$

The term $\varPi _3$ can be bounded similarly to $\varPi _1$, with (52). We find

$$\begin{aligned} \big | \langle \xi , \varPi _3 \xi \rangle \big | \le C N^{-\alpha } (\log N)^{1/2} \Vert (\mathcal{N}_+ + 1)^{1/2} \xi \Vert \Vert \mathcal{K}^{1/2} \xi \Vert \,. \end{aligned}$$

With $|{\widehat{\omega }}_N (r)| \le C$, we similarly obtain

$$\begin{aligned} \begin{aligned} |\langle \xi , \varPi _2 \xi \rangle |&\le N^{-1/2} \Vert \eta \Vert \Vert \mathcal{K}^{1/2} \mathcal{N}_+^{1/2} \xi \Vert \Vert (\mathcal{N}_+ + 1)^{1/2} \xi \Vert \\&\le C N^{-\alpha } \Vert \mathcal{K}^{1/2} \xi \Vert \Vert (\mathcal{N}_+ + 1)^{1/2} \xi \Vert \,. \end{aligned} \end{aligned}$$

Finally, we estimate, using again (52),

$$\begin{aligned} \begin{aligned} \big | \langle \xi , \varPi _4 \xi \rangle \big |&\le N^{-3/2} \Big ( \sum _{r,v,p \in \varLambda _+^*} p^2 |\eta _r|^2 \Vert a_{-p} a_v (\mathcal{N}_+ + 1)^{1/2} \xi \Vert ^2 \Big )^{1/2} \\&\quad \times \Big ( \sum _{r,v,p \in \varLambda _+^*} \frac{|{\widehat{\omega }}_N (p)|^2}{p^2} \Vert a_{-r} a_{r+v} \xi \Vert ^2 \Big )^{1/2} \\&\le C N^{-3/2} \Vert \eta \Vert (\log N)^{1/2} \Vert \mathcal{K}^{1/2} (\mathcal{N}_+ + 1) \xi \Vert \Vert (\mathcal{N}_+ + 1) \xi \Vert \\&\le C N^{-\alpha } (\log N)^{1/2} \Vert \mathcal{K}^{1/2} \xi \Vert \Vert (\mathcal{N}_+ + 1)^{1/2} \xi \Vert \,. \end{aligned} \end{aligned}$$

With (94), we conclude that

$$\begin{aligned} \begin{aligned}&\bigg | \frac{1}{2} \sum _{\begin{array}{c} p \in \varLambda ^* \end{array}} {\widehat{\omega }}_N (p)\; \big [ \langle \xi , e^{-A}\big ( b^*_p b^*_{-p} + b_p b_{-p} \big )e^{A} \xi \rangle - \langle \xi , \big ( b^*_p b^*_{-p} + b_p b_{-p} \big ) \xi \rangle \big ]\bigg |\\&\quad \le C N^{-\alpha } (\log N)^{1/2} \int _0^1 ds\; \Vert \mathcal{K}^{1/2} e^{sA} \xi \Vert \Vert (\mathcal{N}_++1)^{1/2} e^{sA} \xi \Vert \,. \end{aligned} \end{aligned}$$

With Proposition 2, Lemma 3, we conclude that

$$\begin{aligned} \begin{aligned} \Bigg |\frac{1}{2} \sum _{\begin{array}{c} p \in \varLambda ^* \end{array}} {\widehat{\omega }}_N (p)\;&\big [ \langle \xi , e^{-A}\big ( b^*_p b^*_{-p} + b_p b_{-p} \big )e^{A} \xi \rangle - \langle \xi , \big ( b^*_p b^*_{-p} + b_p b_{-p} \big ) \xi \rangle \big ]\Bigg |\\&\le C N^{-\alpha } (\log N)^{1/2} \left[ \Vert \mathcal{H}_N^{1/2} \xi \Vert + N^{1/2} \Vert \mathcal{N}_+^{1/2} \xi \Vert \right] \Vert (\mathcal{N}_++1)^{1/2} \xi \Vert \\&\le C N^{1-\alpha } \Vert (\mathcal{H}_N + 1)^{1/2} \xi \Vert ^2 \,. \end{aligned} \end{aligned}$$

$$\square $$

### Contributions from $e^{-A}\mathcal{C}_N e^{A}$

In Sect. 6.6 we will analyse the contributions to $\mathcal{R}_{N,\alpha }$ arising from conjugation of the cubic operator $\mathcal{C}_N$ defined in (77). To this aim we will need some properties of the commutator $[\mathcal{C}_N, A]$, as established in the following proposition.

#### Proposition 11

Let *A* be defined in (44). Then, there exists a constant $C>0$ such that

$$\begin{aligned} \begin{aligned} \big [\mathcal{C}_N, A \big ] =&\; 2\sum _{r, v\in \varLambda ^*_+} \big [\widehat{V}(r/e^N)\eta _r+\widehat{V}((r+v)/e^N)\eta _r\big ]a^*_va_v\Big (1-\frac{\mathcal{N}_+}{N}\Big ) +\delta _{\mathcal{C}_N} \\ \end{aligned} \end{aligned}$$

where

96 $$\begin{aligned} | \langle \xi , \delta _{\mathcal{C}_N} \xi \rangle |\le \; C N^{3/2-\alpha } \Vert \mathcal{H}_N^{1/2}\xi \Vert \Vert ( \mathcal{N}_++1)^{1/2}\xi \Vert \end{aligned}$$

for all $\alpha > 0$, $\xi \in \mathcal{F}^{\le N}_+$, and $N \in {\mathbb {N}}$ large enough.

#### Proof

We consider the commutator

$$\begin{aligned} \big [\mathcal{C}_N, A \big ] = \sum _{\begin{array}{c} p,q \in \varLambda _+^* : p+q \not = 0 \\ r,v \in \varLambda ^*_+ \end{array}} \widehat{V} (p/e^N) \eta _r \big [ b_{p+q}^* a_{-p}^* a_q , b_{r+v}^* a_{-r}^* a_v - a_v^* a_{-r} b_{r+v} \big ] + \text{ h.c. }\,. \end{aligned}$$

As in the proof of Proposition 9, we use the commutators from the proof of Proposition 8.8 in [4] to conclude that

$$\begin{aligned} \big [\mathcal{C}_N, A \big ] = 2\sum _{r, v\in \varLambda ^*_+} \big [\widehat{V}(r/e^N)\eta _r+\widehat{V}((r+v)/e^N)\eta _r\big ]a^*_va_v\frac{N-\mathcal{N}_+}{N} + \sum _{j=1}^{12} ( \varXi _j + \text{ h.c.}) \end{aligned}$$

where

$$\begin{aligned} \begin{aligned} \varXi _1 :=&\; -\sum _{\begin{array}{c} r, v,p\in \varLambda ^*_+, \\ p\ne v \end{array}}\widehat{V}(p/e^N) \eta _r b^*_{r+v}b^*_{-r} a^*_{-p} a_{v-p},\\ \varXi _2 :=&\; \sum _{\begin{array}{c} r,v,p\in \varLambda ^*_+ \\ r \ne -p \end{array}}\widehat{V}(p/e^N)\eta _r (1-\mathcal{N}_+/N)a^*_{v}a^*_{-p} a_{-r-p} a_{r+v} ,\\ \varXi _3 :=&\; \sum _{\begin{array}{c} r, v, p\in \varLambda _+^*: \\ r+v\ne p \end{array}} \widehat{V}(p/e^N)\eta _r (1-\mathcal{N}_+/N)a_v^* a^*_{-p} a_{-r} a_{r+v-p} ,\\ \varXi _4 :=&\; -\frac{1}{N}\sum _{\begin{array}{c} r,v,p,q\in \varLambda _+^*: p+q \ne 0 \end{array}}\widehat{V}(p/e^N)\eta _r a_v^* a^*_{p+q}a^*_{-p}a_{-r} a_{r+v} a_q,\\ \varXi _5 :=&\; -\frac{1}{N}\sum _{\begin{array}{c} r, v, q \in \varLambda _+^*:\\ r+v \ne q \end{array}} \widehat{V}((r+v)/e^N) \eta _r a_v^*a^*_{q-r-v}a_{-r} a_q , \\ \varXi _6 :=&\; -\frac{1}{N}\sum _{\begin{array}{c} r,v,q \in \varLambda _+^*:\\ r \ne -q \end{array}} \widehat{V}(r/e^N) \eta _r a_v^*a^*_{q+r}a_{r+v} a_q \\ \varXi _7 :=&\; \sum _{\begin{array}{c} r,v, p \in \varLambda _+^*: \\ r+v \ne -p \end{array}} \widehat{V}(p/e^N) \eta _r b^*_{p+r+v} b^*_{-p} a^*_{-r} a_v ,\\ \varXi _{8} :=&\;\sum _{\begin{array}{c} r, v, p\in \varLambda _+^*:\\ r\ne -p \end{array}} \widehat{V}(p/e^N) \eta _r b^*_{p-r}b^*_{r+v}a^*_{-p} a_v ,\\ \varXi _{9} :=&\; -\sum _{\begin{array}{c} r, v, q \in \varLambda _+^*:\\ q\ne v \end{array}} \widehat{V}(v/e^N) \eta _r b^*_{q-v}b^*_{r+v}a^*_{-r} a_q ,\\ \varXi _{10} :=&\; \sum _{\begin{array}{c} r, v, q\in \varLambda _+^*: \\ r\ne -q \end{array}} \widehat{V}(r/e^N) \eta _r b^*_{q+r}a_v^*a_qb_{r+v} ,\\ \varXi _{11} :=&\; -\sum _{\begin{array}{c} r, v, p\in \varLambda _+^*: \\ p\ne -v \end{array}} \widehat{V}(p/e^N) \eta _r b^*_{p+v}a^*_{-p} a_{-r}b_{r+v} ,\\ \varXi _{12} :=&\; \sum _{\begin{array}{c} r, v , q\in \varLambda _+^*: \\ q\ne r+v \end{array}} \widehat{V}((r+v)/e^N) \eta _r b^*_{q-r-v}a_v^* a_{-r} b_q \,. \end{aligned} \end{aligned}$$

To prove the proposition, we have to show that all terms $\varXi _j$, $j=1,\ldots , 12$, satisfy the bound (96). We bound $\varXi _1$ in position space, with Cauchy–Schwarz, by

$$\begin{aligned} \big | \langle \xi , \varXi _1 \xi \rangle \big |\le & {} C\int _{\varLambda ^3}dxdydz e^{2N}V(e^N(x-y))|{\check{\eta }}(x-z)|\Vert {\check{a}}_x\xi \Vert \Vert {\check{a}}_x {\check{a}}_y{\check{a}}_z\xi \Vert \\&\le C\left[ \int _{\varLambda ^3}dxdydz \, e^{2N}V(e^N(x-y))\Vert {\check{a}}_x {\check{a}}_y{\check{a}}_z\xi \Vert ^2\right] ^{1/2}\\&\quad \times \left[ \int _{\varLambda ^3}dxdydz \, e^{2N}V(e^N(x-y))|{\check{\eta }}(x-z)|^2\Vert {\check{a}}_x\xi \Vert ^2\right] ^{1/2}\\&\le C\Vert \eta \Vert \Vert (\mathcal{N}_++1)^{1/2}\xi \Vert \Vert \mathcal{V}_N^{1/2}\mathcal{N}_+^{1/2}\xi \Vert \\&\le CN^{1/2-\alpha }\Vert (\mathcal{N}_++1)^{1/2}\xi \Vert \Vert \mathcal{V}_N^{1/2}\xi \Vert . \end{aligned}$$

We can proceed similarly to control $\varXi _9$. We obtain

$$\begin{aligned} \big | \langle \xi , \varXi _9 \xi \rangle \big | \le C N^{1/2-\alpha } \Vert (\mathcal{N}_++1)^{1/2}\xi \Vert \Vert \mathcal{V}_N^{1/2}\xi \Vert . \end{aligned}$$

The expectations of the terms $\varXi _3$ and $\varXi _{12}$ can be bounded analogously:

$$\begin{aligned}\begin{aligned} \big |&\langle \xi , \varXi _3 \xi \rangle \big | +\big | \langle \xi , \varXi _{12}\xi \rangle \big | \\&\le C\int _{\varLambda ^3}dxdydz \, e^{2N}V(e^N(x-y))(|\eta (x-z)|+|\eta (y-z)|)\Vert {\check{a}}_x{\check{a}}_y\xi \Vert \Vert {\check{a}}_x {\check{a}}_z\xi \Vert \\&\le C\left[ \int _{\varLambda ^3}dxdydz \, e^{2N}V(e^N(x-y))\Vert {\check{a}}_x {\check{a}}_y\xi \Vert ^2(|\eta (x-z)|^2+|\eta (y-z)|^2)\right] ^{1/2}\\&\quad \times \left[ \int _{\varLambda ^3}dxdydz \, e^{2N}V(e^N(x-y))\Vert {\check{a}}_x{\check{a}}_z\xi \Vert ^2\right] ^{1/2}\\&\le C\Vert \eta \Vert \Vert (\mathcal{N}_++1)\xi \Vert \Vert \mathcal{V}_N^{1/2}\xi \Vert \\&\le CN^{1/2-\alpha }\Vert (\mathcal{N}_++1)^{1/2}\xi \Vert \Vert \mathcal{V}_N^{1/2}\xi \Vert . \end{aligned}\end{aligned}$$

As for $\varXi _4$, we find

$$\begin{aligned}\begin{aligned} |\langle \xi , \varXi _4 \xi \rangle | =&\; \bigg |\frac{1}{N} \int _{\varLambda ^2}dxdydz \, e^{2N} V(e^N(y-z)) \langle \xi , {\check{a}}^*_{x}{\check{a}}^*_{y}{\check{a}}^*_{z}{\check{a}}({\check{\eta }}_x){\check{a}}_{x}{\check{a}}_{y} \xi \rangle \bigg |\\&\le C N^{-1}\Vert \eta \Vert \int _{\varLambda ^2}dxdydz \, e^{2N} V(e^N(y-z)) \Vert {\check{a}}_{x}{\check{a}}_{y} {\check{a}}_{z}\xi \Vert \Vert \mathcal{N}_+^{1/2} {\check{a}}_x{\check{a}}_{y}\xi \Vert \\&\le C N^{-1} \Vert \eta \Vert \left[ \int _{\varLambda ^2}dxdydz\, e^{2N} V(e^N(y-z)) \Vert {\check{a}}_{x}{\check{a}}_{y} {\check{a}}_{z}\xi \Vert ^2\right] ^{1/2}\\ {}&\quad \times \left[ \int _{\varLambda ^2}dxdydz\, e^{2N} V(e^N(y-z)) \Vert \mathcal{N}_+^{1/2} {\check{a}}_x{\check{a}}_{y}\xi \Vert ^2\right] ^{1/2}\\&\le C N^{1/2-\alpha }\Vert \mathcal{V}_N^{1/2}\xi \Vert \Vert \mathcal{N}_+^{1/2}\xi \Vert \,. \end{aligned}\end{aligned}$$

The terms $\varXi _5$ and $\varXi _6$ can be bounded in momentum space, using (154). Hence,

$$\begin{aligned}\begin{aligned}&| \langle \xi , \varXi _5 \xi \rangle | + | \langle \xi , \varXi _6 \xi \rangle |\\&\le CN^{-1}\sum _{r, v, q \in \varLambda _+^*} \!\!\! \Bigg ( \frac{\widehat{V}((v+r)/e^N)}{|v|} |\eta _r| |v|\Vert a_{v} a_{q-r -v} \xi \Vert \Vert a_{-r} a_q \xi \Vert \\&\quad + \frac{\widehat{V}(r/e^N)}{|r+v|}|\eta _r| |r+v| \Vert a_{r+q}a_v \xi \Vert \Vert a_{q}a_{r+v} \xi \Vert \Bigg )\\&\le CN^{1/2-\alpha } \Vert (\mathcal{N}_++1)^{1/2}\xi \Vert \Vert \mathcal{K}^{1/2}\xi \Vert . \end{aligned}\end{aligned}$$

Similarly we have

$$\begin{aligned}\begin{aligned} | \langle \xi , \varXi _2 \xi \rangle | + | \langle \xi , \varXi _{10} \xi \rangle |&\le \sum _{r, v, p \in \varLambda _+^*} \!\!\! \Bigg ( \frac{\widehat{V}(p/e^N)}{|p|}|\eta _r| |p|\Vert a_{v}a_{-p} \xi \Vert \Vert a_{r+v}a_{-r-p} \xi \Vert \\&\quad + \frac{\widehat{V}(r/e^N)}{|r+v|}|\eta _r| |r+v| \Vert a_{q}a_{r+v} \xi \Vert \Vert a_{r+q}a_v \xi \Vert \Bigg )\\&\le CN^{3/2-\alpha } \Vert (\mathcal{N}_++1)^{1/2}\xi \Vert \Vert \mathcal{K}^{1/2}\xi \Vert . \end{aligned}\end{aligned}$$

Next, we rewrite $\varXi _7$, $\varXi _8$ and $\varXi _{11}$ as

$$\begin{aligned}\begin{aligned} \varXi _7 =&\; \int _{\varLambda ^2}dxdy\; e^{2N} V(e^N(x-y)){\check{b}}^*_{x}{\check{b}}^*_{y} a^*({\check{\eta }}_x){\check{a}}_{x}\,, \\ \varXi _8 =&\; \int _{\varLambda ^2}dxdydz\; e^{2N} V(e^N(x-y)){\check{\eta }}(z-x){\check{b}}^*_{x}{\check{b}}^*_{z}{\check{a}}^*_y{\check{a}}_{z} \,, \\ \varXi _{11} =&\; - \int _{\varLambda ^2}dxdy\; e^{2N} V(e^N(x-y)){\check{b}}^*_{x}{\check{a}}^*_{y}{\check{a}}(\check{ \eta }_x){\check{b}}_{x} \,. \end{aligned}\end{aligned}$$

Thus, we obtain

$$\begin{aligned}\begin{aligned} |\langle \xi , \varXi _7 \xi \rangle |&\le C\Vert \eta \Vert \int _{\varLambda ^2}dxdy\; e^{2N} V(e^N(x-y))\; \Vert \mathcal{N}_+^{1/2} {\check{a}}_{x}{\check{a}}_{y} \xi \Vert \Vert {\check{a}}_{x} \xi \Vert \\&\le C\Vert \eta \Vert \Vert \mathcal{N}_+^{1/2}\mathcal{V}_N^{1/2}\xi \Vert \Vert \mathcal{N}_+^{1/2}\xi \Vert \\&\le CN^{1/2-\alpha }\Vert \mathcal{V}_N^{1/2}\xi \Vert \Vert \mathcal{N}_+^{1/2}\xi \Vert \,, \end{aligned}\end{aligned}$$

as well as

$$\begin{aligned}\begin{aligned}&|\langle \xi , \varXi _8 \xi \rangle |\\&\le C\int _{\varLambda ^2}dxdydz\; e^{2N} V(e^N(x-y)) |{\check{\eta }}(x-z)|\Vert {\check{a}}_{x}{\check{a}}_{y} {\check{a}}_{z}\xi \Vert \Vert {\check{a}}_z\xi \Vert \\&\le C\left[ \int _{\varLambda ^2}dxdydz\; e^{2N} V(e^N(x-y)) \Vert {\check{a}}_{x}{\check{a}}_{y} {\check{a}}_{z}\xi \Vert ^2\right] ^{1/2}\\ {}&\quad \times \left[ \int _{\varLambda ^2}dxdydz\; e^{2N} V(e^N(x-y)) |\eta (x-z)|^2\Vert {\check{a}}_z\xi \Vert ^2\right] ^{1/2}\\&\le C N^{1/2-\alpha }\Vert \mathcal{V}_N^{1/2}\xi \Vert \Vert \mathcal{N}_+^{1/2}\xi \Vert , \end{aligned}\end{aligned}$$

and

$$\begin{aligned}\begin{aligned} |\langle \xi , \varXi _{11} \xi \rangle |&\le C\Vert \eta \Vert \int _{\varLambda ^2}dxdy\; e^{2N} V(e^N(x-y))\, \Vert {\check{a}}_{x}{\check{a}}_{y} \xi \Vert \Vert \mathcal{N}_+^{1/2}{\check{a}}_{x} \xi \Vert \\&\le C\Vert \eta \Vert \Vert \mathcal{V}_N^{1/2}\xi \Vert \Vert \mathcal{N}_+\xi \Vert \le CN^{1/2-\alpha }\Vert \mathcal{V}_N^{1/2}\xi \Vert \Vert \mathcal{N}_+^{1/2}\xi \Vert . \end{aligned}\end{aligned}$$

Collecting all the bounds above, we arrive at (96). $\square$

### Proof of Proposition 4

With the results of Sects. 6.1–6.5, we can now show Proposition 4. We assume $\alpha > 2$. From Eq. (76), Propositions 8 and 10 we obtain that

$$\begin{aligned} \begin{aligned} \mathcal{R}_{N,\alpha } =\;&e^{-A}\mathcal{G}^{\text {eff}}_{N,\alpha } e^A \\ = \;&\frac{1}{2}\, {\widehat{\omega }}_N(0) (N-1) (1-\mathcal{N}_+/N)+ \left[ 2 N {\widehat{V}}(0)- \frac{1}{2} {\widehat{\omega }}_N(0) \right] \mathcal{N}_+ ( 1- \mathcal{N}_+/N ) \\&+ \frac{1}{2} \sum _{p\in \varLambda ^*_+} {\widehat{\omega }}_N(p)\big [ b^*_p b^*_{-p} + b_p b_{-p} \big ] + \mathcal{K}+ \mathcal{C}_N+ \mathcal{V}_N \\&+ \int _0^1 ds\; e^{-sA}\big [ \mathcal{K}+ \mathcal{C}_N+ \mathcal{V}_N, A \big ]e^{sA} +\mathcal{E}_{\mathcal{R}}^{(1)} \end{aligned} \end{aligned}$$

with

$$\begin{aligned} \pm \mathcal{E}_{\mathcal{R}}^{(1)} \le C N^{1-\alpha } (\mathcal{H}_N + 1). \end{aligned}$$

From Propositions 7, 9 and 11, we can write, for *N* large enough,

$$\begin{aligned} \begin{aligned}&[ \mathcal{K}+ \mathcal{C}_N+ \mathcal{V}_N, A \big ] \\&\quad = \frac{1}{\sqrt{N}} \sum _{\begin{array}{c} r,v\in \varLambda _+^* \end{array}} \!\!\!{\widehat{\omega }}_N(r) \big [ b^*_{r+v}a^*_{-r} a_v+ \text {h.c.}\big ]-\sqrt{N} \sum _{\begin{array}{c} r,v, \in \varLambda ^*_+,\\ p \ne -q \end{array} }\widehat{V}(r/e^N) \big [ b^*_{r+v}a^*_{-r} a_v + \text {h.c.}\big ] \\&\qquad + 2\sum _{r,v \in \varLambda ^*_+} \big [\widehat{V}(r/e^N)\eta _r+\widehat{V}((r+v)/e^N)\eta _r\big ] a^*_v a_v (1-\mathcal{N}_+/N) +\mathcal{E}_{\mathcal{R}}^{(2)} \end{aligned} \end{aligned}$$

where

$$\begin{aligned} \begin{aligned} | \langle \xi , \mathcal{E}_{\mathcal{R}}^{(2)} \xi \rangle | \le&C N^{1/2-\alpha } (\log N)^{1/2} \Vert \mathcal{H}_N^{1/2} \xi \Vert ^2 + C N^{3/2-\alpha } \Vert \mathcal{H}_N^{1/2} \xi \Vert \Vert (\mathcal{N}_++1)^{1/2}\xi \Vert \\&+ C N^{-1}(\log N)^{1/2} \Vert \mathcal{H}_N^{1/2} \xi \Vert \Vert (\mathcal{N}_++1)^{1/2}\xi \Vert \,. \end{aligned}\end{aligned}$$

for all $\xi \in \mathcal{F}^{\le N}_+$. From Proposions 2, 3 and recalling the definition (77) of the operator $\mathcal{C}_N$, we deduce that

97 $$\begin{aligned} \begin{aligned}&\int _0^1 ds\; e^{-sA}[ \mathcal{K}+ \mathcal{C}_N+ \mathcal{V}_N, A \big ] e^{sA} \\&= \int _0^1 ds \; e^{-sA} \Big [-\mathcal{C}_N + \frac{1}{\sqrt{N}}\sum _{\begin{array}{c} r, v\in \varLambda ^*_+ \end{array} } {\widehat{\omega }}_N(r)\big [ b^*_{r+v}a^*_{-r} a_v + \text {h.c.}\big ] \\&\quad + 2\sum _{r,v\in \varLambda ^*_+} \big [\widehat{V}(r/e^N)\eta _r+\widehat{V}((r+v)/e^N)\eta _r\big ] a^*_v a_v\Big (1-\frac{\mathcal{N}_+}{N}\Big )\Big ]e^{sA} + \mathcal{E}_{\mathcal{R}}^{(3)} \end{aligned} \end{aligned}$$

with

$$\begin{aligned} \pm \mathcal{E}_{\mathcal{R}}^{(3)} \le C [ N^{2-\alpha } + N^{-1/2} (\log N)^{1/2} ] (\mathcal{H}_N +1) \end{aligned}$$

for $N \in {\mathbb {N}}$ sufficiently large.

We now rewrite

98 $$\begin{aligned} \begin{aligned} 2\sum _{r,v\in \varLambda ^*_+}&\big [\widehat{V}(r/e^N) \eta _r+\widehat{V}((r+v)/e^N)\eta _r\big ] a^*_v a_v\Big (1-\frac{\mathcal{N}_+}{N}\Big ) \\ =\;&4 \sum _{r,v\in \varLambda ^*_+} \widehat{V}(r/e^N)\eta _r a^*_v a_v\Big (1-\frac{\mathcal{N}_+}{N}\Big ) \\&+ 2\sum _{r,v\in \varLambda ^*_+} \big [\widehat{V}((r+v)/e^N)-\widehat{V}(r/e^N)\big ] \eta _ra^*_v a_v\Big (1-\frac{\mathcal{N}_+}{N}\Big ) := \text {Q}_1 + \text {Q}_2\,. \end{aligned} \end{aligned}$$

With Lemma 1, part (iii) we get

99 $$\begin{aligned} \begin{aligned}&\bigg |2 \sum _{r\in \varLambda ^*} \widehat{V}(r/e^N)\eta _r - \big [ 2 {{\widehat{\omega }}}_N(0) - 2N\widehat{V}(0) \big ] \bigg | \le \frac{C}{N}\,, \end{aligned} \end{aligned}$$

and therefore, using Lemma 3 and (99)

100 $$\begin{aligned} \begin{aligned}&\pm \bigg [ e^{-sA} \text {Q}_1 e^{sA} - 2\big [ 2 {{\widehat{\omega }}}_N(0) - 2N\widehat{V}(0) \big ]\sum _{v\in \varLambda ^*_+}a^*_v a_v\bigg (1-\frac{\mathcal{N}_+}{N}\bigg )\bigg ] \\&\quad \le C N^{1-\alpha } (\mathcal{N}_++1) + \frac{C}{N} \,\mathcal{N}_+\,. \end{aligned} \end{aligned}$$

On the other hand it is easy to check that $e^{-sA} \text {Q}_2 e^{sA}$ is an error term; to this aim we notice that

$$\begin{aligned} \begin{aligned}&\bigg | \sum _{r\in \varLambda ^*} \big [ \widehat{V}(r/e^N)\eta _r- \widehat{V}((r+v)/e^N)\eta _r\big ] \bigg | \le C N |v| e^{-N}\,. \end{aligned} \end{aligned}$$

Hence with Props. 2 and 3 we find

101 $$\begin{aligned} \pm \big [ e^{-sA} \text {Q}_2 e^{sA} \big ] \le C N e^{-N} e^{-sA} \mathcal{N}_+^{1/2} \mathcal{K}^{1/2} e^{sA} \le C N^{2} e^{-N} (\mathcal{H}_N +1) \,. \end{aligned}$$

To handle the second term on the second line of (97), we apply Proposition 9 and then Propositions 2 and 3

102 $$\begin{aligned} \begin{aligned}&\pm \bigg (\frac{1}{\sqrt{N}} \int _0^1 ds\; \sum _{\begin{array}{c} r,v \in \varLambda ^*_+ \end{array} }{\widehat{\omega }}_N(r) \Big [e^{-sA} b^*_{r+v}a^*_{-r} a_ve^{sA}- b^*_{r+v}a^*_{-r} a_v \Big ] + \text {h.c.}\bigg )\\&\quad = \pm \bigg ( \frac{1}{\sqrt{N}}\int _0^1 ds\; \int _0^s dt\;\sum _{\begin{array}{c} r, v\in \varLambda ^*_+ \end{array}} {\widehat{\omega }}_N(r) e^{-tA} \Big [ b^*_{r+v}a^*_{-r} a_v , A \Big ] e^{tA}\bigg ) \\&\quad \le C \int _0^1 ds\; \int _0^s dt\, e^{-tA} \big (N^{-\alpha } (\log N)\, \mathcal{K}+ N^{-1}(\mathcal{N}_++1) \big ) e^{tA} \\&\quad \le C N^{1 -\alpha } \log N (\mathcal{H}_N +1) \,. \end{aligned} \end{aligned}$$

As for the first term on the second line of (97), we use again Proposition 11. Using (98), (100) and (101) we have

103 $$\begin{aligned} \begin{aligned} \int _0^1 ds\; e^{-sA} \mathcal{C}_Ne^{sA } - \mathcal{C}_N&= \int _0^1 ds\; \int _0^{s}dt \; e^{-tA } [\mathcal{C}_N, A] e^{tA} \\&= \big [ 2 {{\widehat{\omega }}}_N(0) - 2N\widehat{V}(0) \big ]\sum _{p\in \varLambda ^*_+}a^*_pa_p\Big (1-\frac{\mathcal{N}_+}{N}\Big ) + \mathcal{E}_{\mathcal{R}}^{(4)} \end{aligned} \end{aligned}$$

with $$\pm \mathcal{E}_{\mathcal{R}}^{(4)} \le C N^{2-\alpha } (\mathcal{H}_N+1) + C N^{-1} (\mathcal{N}_++1)$$.

Inserting the bounds (100), (101), (102) and (103) into (97) we arrive at

$$\begin{aligned} \begin{aligned} \mathcal{R}_{N,\alpha }=&\; \frac{1}{2} (N-1)\, {{\widehat{\omega }}}_N(0) (1-\mathcal{N}_+/N) + \frac{1}{2} {{\widehat{\omega }}}_N(0) \,\mathcal{N}_+ \left( 1 - \mathcal{N}_+/N \right) \\&+ {{\widehat{\omega }}}_N(0) \sum _{p\in \varLambda ^*_+}a^*_pa_p \Big (1-\frac{\mathcal{N}_+}{N} \Big )+ \frac{1}{2} \sum _{p\in \varLambda ^*_+} {\widehat{\omega }}_N(p)\big [ b^*_p b^*_{-p} + b_p b_{-p} \big ] \\&+\frac{1}{\sqrt{N}} \sum _{\begin{array}{c} r,v\in \varLambda ^*_+:\\ r\ne -v \end{array} } {\widehat{\omega }}_N(r)\big [ b^*_{r+v}a^*_{-r} a_v + \text {h.c.}\big ] + \mathcal{H}_N + \mathcal{E}_\mathcal{R}\end{aligned} \end{aligned}$$

with

$$\begin{aligned} \pm \mathcal{E}_\mathcal{R}\le C [ N^{2-\alpha } + N^{-1/2} (\log N)^{1/2} ] (\mathcal{H}_N +1) \end{aligned}$$

for $N \in {\mathbb {N}}$ sufficiently large.

## Appendix A: Analysis of $\mathcal{G}_{N,\alpha }$

The aim of this section is to show Proposition 1. From (12) and (37), we can decompose

$$\begin{aligned} \mathcal{G}_{N,\alpha } = e^{-B} \mathcal{L}_N e^{B} = \mathcal{G}^{(0)}_{N,\alpha } + \mathcal{G}_{N,\alpha }^{(2)} + \mathcal{G}_{N,\alpha }^{(3)} + \mathcal{G}_{N,\alpha }^{(4)} \end{aligned}$$

with

$$\begin{aligned} \mathcal{G}_{N,\alpha }^{(j)} = e^{-B} \mathcal{L}_N^{(j)} e^{B}\,. \end{aligned}$$

To analyse $\mathcal{G}_{N,\alpha }$ we will need precise informations on the action of the generalized Bogoliubov transformation $e^{B}$ with *B* the antisymmetric operator defined in (33), which are summarized in Sect. 1. Then, in the Sects. 1–1 we prove separate bounds for the operators $\mathcal{G}_{N,\alpha }^{(j)}$, $j=0,2,3,4$, which we combine in Sect. 1 to prove Proposition 1.

The analysis in this section follows closely that of [4, Sect. 7] with some slight modifications due to the different scaling of the interaction potential and the fact that the kernel $\eta _p$ of $e^{B}$ is different from zero for all $p \in \varLambda ^*_+$ (in [4] $\eta _p$ is different from zero only for momenta larger than a sufficiently large cutoff of order one). Moreover, while in three dimensions it was sufficient to choose the function $\eta _p$ appearing in the generalized Bogoliubov transformation with $\Vert \eta \Vert$ sufficiently small but of order one, we need here $\Vert \eta \Vert$ to be of order $N^{-\alpha }$ for some $\alpha >0$ large enough. As discussed in the introduction this is achieved by considering the Neumann problem for the scattering equation in (16) on a ball of radius $\ell =N^{-\alpha }$; as a consequence some terms depending on $\ell$ will be large, compared to the analogous terms in [4].

### Appendix A.1: Generalized Bogoliubov Transformations

In this subsection we collect important properties about the action of unitary operators of the form $e^{B}$, as defined in (34). As shown in [2, Lemma 2.5 and 2.6], we have, if $\Vert \eta \Vert$ is sufficiently small,

104 $$\begin{aligned} \begin{aligned} e^{-B} b_p e^{B}&= \sum _{n=0}^\infty \frac{(-1)^n}{n!} \text {ad}_{B}^{(n)} (b_p) \\ e^{-B} b^*_p e^{B}&= \sum _{n=0}^\infty \frac{(-1)^n}{n!} \text {ad}_{B}^{(n)} (b^*_p) \end{aligned} \end{aligned}$$

where the series converge absolutely. To confirm the expectation that generalized Bogoliubov transformation act similarly to standard Bogoliubov transformations, on states with few excitations, we define (for $\Vert \eta \Vert$ small enough) the remainder operators

105 $$\begin{aligned} d_q =\sum _{m\ge 0}\frac{1}{m!} \Big [\text {ad}_{-B}^{(m)}(b_q) - \eta _q^m b_{\alpha _m q}^{\sharp _m } \Big ],\quad d^*_q =\sum _{m\ge 0}\frac{1}{m!} \Big [\text {ad}_{-B}^{(m)}(b^*_q) - \eta _q^m b_{\alpha _m q}^{\sharp _{m+1}} \Big ] \end{aligned}$$

where $q \in \varLambda ^*_+$, $(\sharp _m, \alpha _m) = (\cdot , +1)$ if *m* is even and $(\sharp _m, \alpha _m) = (*, -1)$ if *m* is odd. It follows then from (104) that

106 $$\begin{aligned} e^{-B} b_q e^{B} = \gamma _q b_q +\sigma _q b^*_{-q} + d_q, \quad e^{-B} b^*_q e^{B} = \gamma _q b^*_q +\sigma _q b_{-q} + d^*_q \end{aligned}$$

where we introduced the notation $\gamma _q = \cosh (\eta _q)$ and $\sigma _q = \sinh (\eta _q)$. It will also be useful to introduce remainder operators in position space. For $x \in \varLambda$, we define the operator valued distributions ${\check{d}}_x, {\check{d}}^*_x$ through

107 $$\begin{aligned} e^{-B} {\check{b}}_x e^{B} = b ( {\check{\gamma }}_x) + b^* ({\check{\sigma }}_x) + {\check{d}}_x, \qquad e^{-B} {\check{b}}^*_x e^{B} = b^* ( {\check{\gamma }}_x) + b ({\check{\sigma }}_x) + {\check{d}}^*_x \end{aligned}$$

where ${\check{\gamma }}_x (y) = \sum _{q \in \varLambda ^*} \cosh (\eta _q) e^{iq \cdot (x-y)}$ and ${\check{\sigma }}_x (y) = \sum _{q \in \varLambda ^*} \sinh (\eta _q) e^{iq \cdot (x-y)}$. The next lemma is taken from [4, Lemma 3.4].

#### Lemma 4

Let $\eta \in \ell ^2 (\varLambda _+^*)$, $n \in {\mathbb {Z}}$. For $p \in \varLambda _+^*$, let $d_p$ be defined as in (106). If $\Vert \eta \Vert$ is small enough, there exists $C > 0$ such that

108 $$\begin{aligned} \begin{aligned} \Vert (\mathcal{N}_+ + 1)^{n/2} d_p \xi \Vert&\le \frac{C}{N} \left[ |\eta _p| \Vert (\mathcal{N}_+ + 1)^{(n+3)/2} \xi \Vert + \Vert \eta \Vert \Vert b_p (\mathcal{N}_+ + 1)^{(n+2)/2} \xi \Vert \right] , \\ \Vert (\mathcal{N}_+ + 1)^{n/2} d_p^* \xi \Vert&\le \frac{C}{N} \, \Vert \eta \Vert \,\Vert (\mathcal{N}_+ +1)^{(n+3)/2} \xi \Vert \end{aligned} \end{aligned}$$

for all $p \in \varLambda ^*_+, \xi \in \mathcal{F}_+^{\le N}$. In position space, with ${\check{d}}_x$ defined as in (107), we find

109 $$\begin{aligned} \Vert (\mathcal{N}_+ + 1)^{n/2} {\check{d}}_x \xi \Vert \le \frac{C }{N}\, \Vert \eta \Vert \Big [ \,\Vert (\mathcal{N}_+ + 1)^{(n+3)/2} \xi \Vert + \Vert b_x (\mathcal{N}_+ + 1) ^{(n+2)/2}\xi \Vert \,. \Big ] \end{aligned}$$

Furthermore, letting $\check{\overline{d}}_x = {\check{d}}_x + (\mathcal{N}_+ / N) b^*({\check{\eta }}_x)$, we find

110 $$\begin{aligned} \begin{aligned} \Vert (\mathcal{N}_+&+ 1)^{n/2} {\check{a}}_y \check{\overline{d}}_x \xi \Vert \\&\le \frac{C}{N} \, \Big [ \, \Vert \eta \Vert ^2 \Vert (\mathcal{N}_+ + 1)^{(n+2)/2} \xi \Vert + \Vert \eta \Vert |{\check{\eta }} (x-y)| \Vert (\mathcal{N}+1)^{(n+2)/2} \xi \Vert \\&\quad + \Vert \eta \Vert \Vert {\check{a}}_x (\mathcal{N}_++1)^{(n+1)/2} \xi \Vert + \Vert \eta \Vert ^2 \Vert {\check{a}}_y (\mathcal{N}_+ + 1)^{(n+3)/2} \xi \Vert \\&\quad + \Vert \eta \Vert \Vert {\check{a}}_x {\check{a}}_y (\mathcal{N}+1)^{(n+2)/2} \xi \Vert \, \Big ] \end{aligned} \end{aligned}$$

and, finally,

111 $$\begin{aligned} \begin{aligned} \Vert (\mathcal{N}_+&+ 1)^{n/2} {\check{d}}_x {\check{d}}_y \xi \Vert \\&\le \frac{C}{N^2} \Big [ \; \Vert \eta \Vert ^2 \Vert (\mathcal{N}_++ 1)^{(n+6)/2} \xi \Vert + \Vert \eta \Vert |{\check{\eta }} (x-y)| \Vert (\mathcal{N}_+ + 1)^{(n+4)/2} \xi \Vert \\&\quad + \Vert \eta \Vert ^2 \Vert {a}_x (\mathcal{N}_+ + 1)^{(n+5)/2} \xi \Vert + \Vert \eta \Vert ^2 \Vert {a}_y (\mathcal{N}_+ + 1)^{(n+5)/2} \xi \Vert \\&\quad + \Vert \eta \Vert ^2\, \Vert {a}_x {a}_y (\mathcal{N}_+ + 1)^{(n+4)/2} \xi \Vert \; \Big ] \end{aligned} \end{aligned}$$

for all $$\xi \in \mathcal{F}^{\le n}_+$$.

A first simple application of Lemma 4 is the following bound on the growth of the expectation of $\mathcal{N}_+$.

#### Lemma 5

Assume *B* is defined as in (33), with $\eta \in \ell ^2 (\varLambda ^*)$ and $\eta _p = \eta _{-p}$ for all $p \in \varLambda ^*_+$. Then, there exists a constant $C > 0$ such that

$$\begin{aligned} \Big | \langle \xi , \big [ e^{-B} \mathcal{N}_+ e^{B} - \mathcal{N}_+ \big ] \xi \rangle \Big | \le \Vert \eta \Vert \Vert (\mathcal{N}_+ + 1)^{1/2} \xi \Vert ^2 \end{aligned}$$

for all $$\xi \in \mathcal{F}_+^{\le N}$$.

#### Proof

With (106) we write

$$\begin{aligned} \begin{aligned} e^{-B}&\mathcal{N}_+ e^{B} - \mathcal{N}_+ \\ = \;&\int _0^1 e^{-sB} [\mathcal{N}_+ ,B] e^{s B}ds \\ =\;&\int _0^1 \sum _{p \in \varLambda ^*_+} \eta _p \, e^{-s B} ( b_p b_{-p} + b^*_p b^*_{-p} ) e^{s B} \, ds \\ = \;&\int _0^1 \sum _{p \in \varLambda ^*_+} \eta _p \, \left[ (\gamma _p^{(s)} b_p + \sigma _p^{(s)} b_{-p}^* + d_p^{(s)}) ( \gamma _p^{(s)} b_{-p} + \sigma _p^{(s)} b_{-p}^* + d_{-p}^{(s)}) + \text{ h.c. }\right] ds \end{aligned} \end{aligned}$$

with $\gamma _p^{(s)} = \cosh (s \eta _p)$, $\sigma _p^{(s)} = \sinh (s \eta _p)$. Using $|\gamma ^{(s)}_p| \le C$ and $|\sigma _p^{(s)}| \le C |\eta _p|$, (108) in Lemma 4 we arrive at

$$\begin{aligned} \begin{aligned} \Big |&\langle \xi , \big [ e^{-B} \mathcal{N}_+ e^{B} - \mathcal{N}_+ \big ] \xi \rangle \Big | \\&\le C \Vert (\mathcal{N}_+ + 1)^{1/2} \xi \Vert \sum _{p \in \varLambda ^*_+} |\eta _p| \left[ |\eta _p| \Vert (\mathcal{N}_+ + 1)^{1/2} \xi \Vert + \Vert b_{p} \xi \Vert \right] \le C \Vert \eta \Vert \Vert (\mathcal{N}_+ + 1)^{1/2} \xi \Vert ^2 \end{aligned} \end{aligned}$$

$$\square $$

### Appendix A.2: Analysis of $\mathcal{G}_{N,\alpha }^{(0)}=e^{-B}\mathcal{L}^{(0)}_N e^{B}$

We define $\mathcal{E}_{N}^{(0)}$ so that

$$\begin{aligned} \begin{aligned} \mathcal{G}^{(0)}_{N,\alpha }&= e^{-B} \mathcal{L}^{(0)}_N e^{B}= \frac{1}{2} \widehat{V} (0) (N+\mathcal{N}_+-1)(N-\mathcal{N}_+) + \mathcal{E}_{N,\alpha }^{(0)}\,. \end{aligned} \end{aligned}$$

where we recall from (13) that

$$\begin{aligned} \mathcal{L}_{N}^{(0)} =\;\frac{1}{2} \widehat{V} (0) (N-1 + \mathcal{N}_+)(N-\mathcal{N}_+ )\,. \end{aligned}$$

#### Proposition 12

Under the assumptions of Proposition 1, there exists a constant $C > 0$ such that

$$\begin{aligned} \pm \mathcal{E}_{N, \alpha }^{(0)} \le C N^{1-\alpha } (\mathcal{N}_+ +1) \end{aligned}$$

for all $\alpha > 0$ and $N \in {\mathbb {N}}$ large enough.

#### Proof

The proof follows [4, Prop. 7.1].

We write

$$\begin{aligned} \mathcal{L}_N^{(0)} = \frac{N(N-1)}{2} \widehat{V} (0) + \frac{N}{2}\widehat{V} (0) \Big [ \sum _{q \in \varLambda ^*_+} b_q^* b_q - \mathcal{N}_+ \Big ]\,. \end{aligned}$$

Hence,

$$\begin{aligned} \begin{aligned} \mathcal{E}_{N}^{(0)}&= \frac{N}{2}\widehat{V} (0) \sum _{q \in \varLambda _+^*} \left[ e^{-B} b_q^* b_q e^{B} - b_q^* b_q \right] - \frac{N}{2} \widehat{V} (0)\left[ e^{-B} \mathcal{N}_+ e^{B} - \mathcal{N}_+ \right] \,. \end{aligned} \end{aligned}$$

To bound the first term we use (106), $|\gamma _q^2 - 1| \le C \eta _q^2$, $|\sigma _q| \le C |\eta _q|$, the first bound in (108), Cauchy–Schwarz and the estimate $\Vert \eta \Vert \le C N^{-\alpha }$. To bound the second term, we use Lemma 5. We conclude that

$$\begin{aligned} |\langle \xi , \mathcal{E}_N^{(0)} \xi \rangle | \le C N^{1-\alpha } \Vert (\mathcal{N}_+ + 1)^{1/2} \xi \Vert ^2\,. \end{aligned}$$

$$\square $$

### Appendix A.3: Analysis of $\mathcal{G}_{N,\alpha }^{(2)}=e^{-B}\mathcal{L}^{(2)}_N e^{B}$

We consider first conjugation of the kinetic energy operator.

#### Proposition 13

Under the assumptions of Proposition 1, there exists $C > 0$ such that

112 $$\begin{aligned} \begin{aligned} e^{-B}\mathcal{K}e^{B} = \;&\mathcal{K}+ \sum _{p \in \varLambda ^*_+} p^2 \eta _p ( b_p b_{-p} + b^*_p b^*_{-p} ) \\ {}&+ \sum _{p \in \varLambda ^*_+} p^2 \eta _p^2 \Big (\frac{N-\mathcal{N}_+}{N}\Big ) \Big (\frac{N-\mathcal{N}_+ -1}{N}\Big ) +\mathcal{E}^{(K)}_{N,\alpha } \end{aligned} \end{aligned}$$

where

113 $$\begin{aligned} \begin{aligned} | \langle \xi , \mathcal{E}^{(K)}_{N,\alpha } \xi \rangle | \le C N^{1/2 -\alpha } \Vert \mathcal{H}_N^{1/2}\xi \Vert \Vert (\mathcal{N}_++1)^{1/2}\xi \Vert + C N^{1-\alpha } \Vert (\mathcal{N}_++1)^{1/2}\xi \Vert ^2 \end{aligned} \end{aligned}$$

for any $\alpha >1$, $\xi \in \mathcal{F}^{\le N}_+$ and $N \in {\mathbb {N}}$ large enough.

#### Proof

We proceed as in the proof of [4, Prop. 7.2]. We write

114 $$\begin{aligned} \begin{aligned}&e^{-B} \mathcal{K}e^{B}-\mathcal{K}\\&= \int _0^1ds \sum _{p \in \varLambda ^*_+} p^2 \eta _p \Big [\big (\gamma _p^{(s)} b_p+ \sigma ^{(s)}_p b^*_{-p}\big ) \big (\gamma _p^{(s)} b_{-p} + \sigma ^{(s)}_p b^*_{p}\big )\, +\text{ h.c. }\Big ]\\&\quad + \int _0^1ds \sum _{p \in \varLambda ^*_+} p^2 \eta _p \big [\big (\gamma _p^{(s)} b_p+ \sigma ^{(s)}_p b^*_{-p}\big ) d_{-p}^{(s)}+ d_p^{(s)} \big ( \gamma _p^{(s)} b_{-p}+ \sigma ^{(s)}_p b^*_{p}\big )+\text{ h.c. }\big ]\\&\quad + \int _0^1ds \sum _{p \in \varLambda ^*_+} p^2 \eta _p\big [ d_p^{(s)} d_{-p}^{(s)} + \text{ h.c. }\big ]\\&=: \text {G}_1+\text {G}_2+\text {G}_3 \end{aligned} \end{aligned}$$

with $\gamma _p^{(s)} = \cosh (s \eta _p)$, $\sigma ^{(s)}_p =\sinh (s \eta _p)$ and where $d^{(s)}_p$ is defined as in (105), with $\eta _p$ replaced by $s \eta _p$. We find

$$\begin{aligned} \begin{aligned} \text {G}_1&= \sum _{p \in \varLambda ^*_+} p^2 \eta _p \big (b_p b_{-p} + b^*_{-p} b^*_{p} \big )+ \sum _{p \in \varLambda ^*_+} p^2 \eta _p^2 \left( 1-\frac{\mathcal{N}_+}{N}\right) +\mathcal{E}^K_{1} \end{aligned} \end{aligned}$$

with

$$\begin{aligned} \begin{aligned} \mathcal{E}^K_{1}=&\, 2 \int _0^1ds \sum _{p \in \varLambda ^*_+} p^2 \eta _p (\sigma ^{(s)}_p)^2 \big (b_p b_{-p} + b^*_{-p} b^*_{p} \big )\\&+\int _0^1ds \sum _{p \in \varLambda ^*_+} p^2 \eta _p \gamma _p^{(s)} \sigma ^{(s)}_p (4b_p^*b_{p}-2N^{-1}a^*_pa_p)\\&+2\int _0^1ds \sum _{p \in \varLambda ^*_+} p^2 \eta _p \left[ (\gamma _p^{(s)}-1) \sigma ^{(s)}_p + (\sigma ^{(s)}_p-s \eta _p) \right] \Big (1-\frac{\mathcal{N}_+}{N}\Big ) \,. \end{aligned} \end{aligned}$$

Since $|\big ((\gamma _p^{(s)})^2-1\big )|\le C \eta _p^2$, $(\sigma ^{(s)}_p)^2\le C \eta _p^2$, $p^2 |\eta _p| \le C$, $\Vert \eta \Vert _\infty \le N^{-\alpha }$, we can estimate

115 $$\begin{aligned} |\langle \xi ,&\mathcal{E}^K_{1} \xi \rangle | \nonumber \\ \le \;&C \sum _{p \in \varLambda ^*_+} p^2 |\eta _p|^3 \Vert b_p\xi \Vert \Vert (\mathcal{N}_++1)^{1/2} \xi \Vert +C \sum _{p \in \varLambda ^*_+} p^2 \eta _p^2 \Vert a_p\xi \Vert ^2+C\sum _{p \in \varLambda ^*_+} p^2 \eta _p^4 \Vert \xi \Vert ^2 \nonumber \\\\ \le \;&C\Vert \eta \Vert \Vert (\mathcal{N}_++1)^{1/2} \xi \Vert ^2 \le C N^{-\alpha } \Vert (\mathcal{N}_++1)^{1/2} \xi \Vert ^2,\nonumber \end{aligned}$$

for any $\xi \in \mathcal{F}_+^{\le N}$. To bound the term $\text {G}_3$ in (114), we switch to position space:

$$\begin{aligned} \begin{aligned} | \langle \xi , \text {G}_3 \xi \rangle | \le \;&CN \int _0^1 ds \int _{\varLambda ^2} dx dy \left[ e^{2N} V(e^N(x-y)) + N^{2\alpha -1} \chi (|x-y|\le N^{-\alpha }) \right] \\&\quad \times \Vert (\mathcal{N}_+ + 1)^{-1/2} {\check{d}}^{(s)}_x {\check{d}}^{(s)}_y \xi \Vert \Vert (\mathcal{N}_+ + 1)^{1/2} \xi \Vert \end{aligned} \end{aligned}$$

With (111), we obtain

116 $$\begin{aligned} \begin{aligned}&| \langle \xi , \text {G}_3 \xi \rangle | \\&\le C N^{1-\alpha } \int _{\varLambda ^2} dx dy \left[ e^{2N} V(e^N(x-y)) + N^{2\alpha -1}\chi (|x-y|\le N^{-\alpha }) \right] \Vert (\mathcal{N}_+ + 1)^{1/2} \xi \Vert ^2 \\&\quad + C N^{-2\alpha }\int _{\varLambda ^2} dx dy \left[ e^{2N} V(e^N(x-y)) + N^{2\alpha -1}\chi (|x-y|\le N^{-\alpha }) \right] \Vert (\mathcal{N}_+ + 1)^{1/2} \xi \Vert \\&\quad \times \Big [ \Vert {\check{a}}_x (\mathcal{N}_++1)\xi \Vert + \Vert {\check{a}}_y (\mathcal{N}_++1) \xi \Vert + \Vert {\check{a}}_x {\check{a}}_y (\mathcal{N}_++1)^{1/2} \xi \Vert \Big ] \\&\le C N^{1-\alpha } \Vert (\mathcal{N}_+ + 1)^{1/2} \xi \Vert ^2 + C N^{1/2-\alpha } \Vert (\mathcal{N}_+ + 1)^{1/2} \xi \Vert \Vert \mathcal{V}_N^{1/2} \xi \Vert \,. \end{aligned}\nonumber \\ \end{aligned}$$

Finally, we consider $\text {G}_2$ in (114). We split it as $\text {G}_2 = \text {G}_{21} + \text {G}_{22} + \text {G}_{23} + \text {G}_{24}$, with

117 $$\begin{aligned} \begin{aligned} \text {G}_{21}&= \int _0^1ds \sum _{p \in \varLambda ^*_+} p^2 \eta _p \left( \gamma _p^{(s)} b_p d_{-p}^{(s)} + \text{ h.c. }\right) , \\ \text {G}_{22}&= \int _0^1ds \sum _{p \in \varLambda ^*_+} p^2 \eta _p \left( \sigma ^{(s)}_p b^*_{-p} d_{-p}^{(s)} + \text{ h.c. }\right) \\ \text {G}_{23}&= \int _0^1ds \sum _{p \in \varLambda ^*_+} p^2 \eta _p \left( \gamma _p^{(s)} d_p^{(s)} b_{-p}+ \text{ h.c. }\right) , \\ \text {G}_{24}&= \int _0^1ds \sum _{p \in \varLambda ^*_+} p^2 \eta _p \left( \sigma ^{(s)}_p d_p^{(s)} b^*_{p}+ \text{ h.c. }\right) \,. \end{aligned} \end{aligned}$$

We consider $\text {G}_{21}$ first. We write

$$\begin{aligned} \text {G}_{21} = \; - \sum _{p \in \varLambda ^*_+} p^2 \eta _p^2 \, \frac{\mathcal{N}_+ + 1}{N} \frac{N-\mathcal{N}_+}{N} + \left[ \mathcal{E}_{2}^K + \text{ h.c. }\right] \end{aligned}$$

where $$\mathcal{E}_{2}^K = \sum _{j=1}^3 \mathcal{E}_{2j}^K$$, with

118 $$\begin{aligned} \begin{aligned} \mathcal{E}_{21}^K = \;&\frac{1}{2N} \sum _{p \in \varLambda ^*_+} p^2 \eta _p^2 (\mathcal{N}_++1) \big ( b^*_p b_p - \frac{1}{N} a_p^* a_p\big ) , \\ \mathcal{E}_{22}^K = \;&\int _0^1 ds \sum _{p \in \varLambda ^*_+} p^2 \eta _p (\gamma _p^{(s)} - 1) b_p d_{-p}^{(s)} \,, \\ \mathcal{E}_{23}^K = \;&\int _0^1 ds \sum _{p \in \varLambda _+^*} p^2 \eta _p b_p \overline{d}_{-p}^{(s)} \,. \end{aligned} \end{aligned}$$

and where we introduced the notation $\overline{d}^{(s)}_{-p} = d_{-p}^{(s)} + s \eta _p (\mathcal{N}_+ / N) b_p^*$. With (29), we find

119 $$\begin{aligned} |\langle \xi , \mathcal{E}_{21}^K \xi \rangle | \le C \sum _{p \in \varLambda ^*_+} \eta _p \Vert a_p \xi \Vert ^2 \le C N^{-\alpha }\Vert \mathcal{N}_+^{1/2} \xi \Vert ^2 \end{aligned}$$

Using $|\gamma _p^{(s)} - 1| \le C \eta _p^2$ and (108), we obtain

120 $$\begin{aligned} \begin{aligned} |\langle \xi , \mathcal{E}_{22}^K \xi \rangle |&\le \sum _{p \in \varLambda ^*_+} p^2 |\eta _p|^3 \Vert \mathcal{N}_+^{1/2} \xi \Vert \Vert d^{(s)}_{-p} \xi \Vert \le C N^{-3\alpha } \Vert (\mathcal{N}_+ + 1)^{1/2} \xi \Vert ^2. \end{aligned} \end{aligned}$$

To control the third term in (118), we use (30) and we switch to position space. We find

121 $$\begin{aligned} \begin{aligned} \mathcal{E}_{23}^K = \;&-N \int _0^1 ds \int _{\varLambda ^2} dx dy \, e^{2N} V(e^N(x-y)) f_{N,\ell } (x-y) {\check{b}}_x \check{\overline{d}}^{\,(s)}_y \\&+ N \int _0^1 ds e^{2N} \lambda _\ell \int _{\varLambda ^2} dx dy \, \chi _\ell (x-y) f_{N,\ell } (x-y) {\check{b}}_x \check{\overline{d}}^{\,(s)}_y \\ =;&\mathcal{E}_{231}^K+\mathcal{E}_{232}^K \,. \end{aligned} \end{aligned}$$

With (110) and $|{{\check{\eta }}}(x-y)| \le C N$, we obtain

122 $$\begin{aligned} \begin{aligned} |\langle \xi , \mathcal{E}_{231}^K \xi \rangle | \le \;&N \int _0^1 ds \int _{\varLambda ^2} dx dy \, e^{2N} V(e^N(x-y)) \\&\quad \times \Vert (\mathcal{N}_+ + 1)^{1/2} \xi \Vert \Vert (\mathcal{N}_+ + 1)^{-1/2} {\check{a}}_x \check{\overline{d}}^{(s)}_y \xi \Vert \\ \le \;&C N^{1-\alpha } \Vert (\mathcal{N}_+ + 1)^{1/2} \xi \Vert ^2 + C N^{1/2-\alpha } \Vert (\mathcal{N}_+ + 1)^{1/2} \xi \Vert \Vert \mathcal{V}_N^{1/2} \xi \Vert . \end{aligned} \end{aligned}$$

As for $\mathcal{E}_{232}^K$, with (110) and Lemma 1 (recalling $\ell = N^{-\alpha })$, we find

123 $$\begin{aligned} \begin{aligned} |\langle \xi , \mathcal{E}_{232}^K \xi \rangle | \le \;&C N^{-\alpha } \Vert (\mathcal{N}_+ + 1)^{1/2} \xi \Vert ^2 \\&+ \int _{\varLambda ^2} dx dy \, \chi (|x-y| \le N^{-\alpha })\Vert (\mathcal{N}_+ + 1)^{1/2} \xi \Vert \Vert {\check{a}}_x {\check{a}}_y \mathcal{N}_+^{1/2} \xi \Vert \end{aligned} \end{aligned}$$

To bound the last term on the r.h.s. of (123) we use Hölder’s and Sobolev inequality $\Vert u \Vert _q \le C q^{1/2} \Vert u \Vert _{H^1}$, valid for any $2 \le q <\infty$. We find

$$\begin{aligned} \begin{aligned} \int _{\varLambda ^2}&dx dy \, \chi (|x-y| \le N^{-\alpha })\Vert (\mathcal{N}_+ + 1)^{1/2} \xi \Vert \Vert {\check{a}}_x {\check{a}}_y \mathcal{N}_+^{1/2} \xi \Vert \\&\le C \Vert (\mathcal{N}_+ + 1)^{1/2} \xi \Vert \int _{\varLambda } dx \left( \int _{\varLambda } dy \, \chi (|x-y| \le N^{-\alpha })\right) ^{1-1/q}\left( \int _{\varLambda } dy \, \Vert {\check{a}}_x {\check{a}}_y \mathcal{N}_+^{1/2} \xi \Vert ^q\right) ^{1/q}\\&\le CN^{2\alpha /q -2\alpha } \Vert (\mathcal{N}_+ + 1)^{1/2} \xi \Vert \int _{\varLambda } dx \left( \int _{\varLambda }dy \, \Vert {\check{a}}_x {\check{a}}_y \mathcal{N}_+^{1/2} \xi \Vert ^q\right) ^{1/q}\\&\le C q^{1/2}N^{2\alpha /q -2\alpha } \Vert (\mathcal{N}_+ + 1)^{1/2} \xi \Vert \\&\quad \times \left[ \int _{\varLambda ^2} dxdy \, \Vert {\check{a}}_x\nabla _y{\check{a}}_y\mathcal{N}_+^{1/2}\xi \Vert ^2+\int _{\varLambda ^2} dxdy \, \Vert {\check{a}}_x {\check{a}}_y \mathcal{N}_+^{1/2} \xi \Vert ^2\right] ^{1/2} \\&\le Cq^{1/2}N^{2\alpha /q-2\alpha } \Vert (\mathcal{N}_+ + 1)^{1/2} \xi \Vert \left[ \ \Vert \mathcal{K}^{1/2}\mathcal{N}_+ \xi \Vert +\Vert \mathcal{N}_+^{3/2} \xi \Vert \right] \,. \end{aligned}\end{aligned}$$

Choosing $q=\log N$, we get

124 $$\begin{aligned} \begin{aligned} \int _{\varLambda ^2}&dx dy \, \chi (|x-y| \le N^{-\alpha })\Vert (\mathcal{N}_+ + 1)^{1/2} \xi \Vert \Vert {\check{a}}_x {\check{a}}_y (\mathcal{N}_++1)^{1/2} \xi \Vert \\ \le&\; CN^{1-2\alpha }(\log N)^{1/2} \Vert (\mathcal{N}_+ + 1)^{1/2} \xi \Vert \Vert \mathcal{K}^{1/2} \xi \Vert . \end{aligned} \end{aligned}$$

Therefore, for any $\xi \in \mathcal{F}^{\le N}_+$,

$$\begin{aligned} |\langle \xi , \mathcal{E}_{232}^K \xi \rangle | \le \; N^{1-2\alpha } (\log N)^{1/2} \Vert \mathcal{K}^{1/2}\xi \Vert \Vert (\mathcal{N}_+ + 1)^{1/2} \xi \Vert + N^{-\alpha } \Vert (\mathcal{N}_+ + 1)^{1/2} \xi \Vert ^2 \,. \end{aligned}$$

Combining the last bound with (119), (120) and (122), we conclude that

125 $$\begin{aligned} \begin{aligned} | \langle \xi , \mathcal{E}_2^K \xi \rangle | \le C N^{1-\alpha } \Vert (\mathcal{N}_+ + 1)^{1/2} \xi \Vert ^2 + C N^{1/2-\alpha }\Vert \mathcal{H}_N^{1/2} \xi \Vert \Vert (\mathcal{N}_+ + 1)^{1/2} \xi \Vert \,. \end{aligned} \end{aligned}$$

for any $\alpha >1$, $N \in {\mathbb {N}}$ large enough, $\xi \in \mathcal{F}^{\le N}_+$.

The term $\text {G}_{22}$ in (117) can be bounded using (108). We find

126 $$\begin{aligned} \begin{aligned} |\langle \xi ,\text {G}_{22}\xi \rangle |&\le CN^{-2\alpha }\Vert (\mathcal{N}_++1)^{1/2}\xi \Vert ^2\,. \end{aligned} \end{aligned}$$

We split $\text {G}_{23} = \mathcal{E}_{31}^K + \mathcal{E}_{32}^K + \text{ h.c. }$, with

$$\begin{aligned} \begin{aligned} \mathcal{E}_{31}^K = \;&\int _0^1ds \sum _{p \in \varLambda ^*_+} p^2 \eta _p \big (\gamma _p^{(s)}-1\big ) d_p^{(s)}b_{-p} \, , \quad \mathcal{E}_{32}^K = \int _0^1ds \sum _{p \in \varLambda _+^*} p^2 \eta _p d_p^{(s)} b_{-p} \end{aligned} \end{aligned}$$

With (108), we find

$$\begin{aligned} \begin{aligned} |\langle \xi , \mathcal{E}_{31}^K \xi \rangle |&\le C \int _0^1ds\; \sum _{p \in \varLambda ^*_+} p^2 |\eta _p |^3 \Vert (d_p^{(s)})^* \xi \Vert \Vert b_{-p} \xi \Vert ds \le C N^{-3\alpha } \Vert (\mathcal{N}_+ + 1)^{1/2} \xi \Vert ^2 \end{aligned} \end{aligned}$$

To estimate $\mathcal{E}_{32}^K$, we use (30) and we switch to position space. Proceeding as we did in (121), (122), (123), we obtain

$$\begin{aligned}\begin{aligned} |\langle \xi , \mathcal{E}_{32}^K \xi \rangle |&\le CN\int _0^1 ds \int _{\varLambda ^2} dx dy \left[ e^{2N} V(e^N(x-y)) + N^{2\alpha -1} \chi (|x-y| \le N^{-\alpha }) \right] \\&\quad \times \Vert (\mathcal{N}_+ + 1)^{1/2} \xi \Vert \Vert (\mathcal{N}_+ + 1)^{-1/2} {\check{d}}_x^{(s)} {\check{b}}_y \xi \Vert \,. \end{aligned} \end{aligned}$$

With (109) and (124) we find

$$\begin{aligned}\begin{aligned} |\langle \xi , \mathcal{E}_{32}^K \xi \rangle |&\le C N^{-\alpha } \int _{\varLambda ^2} dx dy \left[ e^{2N} V(e^N(x-y)) + N^{2\alpha -1} \chi (|x-y|\le N^{-\alpha }) \right] \\&\quad \times \Vert (\mathcal{N}_++1)^{1/2} \xi \Vert \left[ \Vert {\check{a}}_y (\mathcal{N}_++1) \xi \Vert + \Vert {\check{a}}_x {\check{a}}_y (\mathcal{N}_++1)^{1/2} \xi \Vert \right] \\&\le C N^{1-\alpha } \Vert (\mathcal{N}_+ + 1)^{1/2} \xi \Vert ^2 + C N^{1/2-\alpha } \Vert (\mathcal{N}_+ + 1)^{1/2} \xi \Vert \Vert \mathcal{V}_N^{1/2} \xi \Vert \\&\quad + C N^{1-2\alpha } (\log N)^{1/2} \Vert (\mathcal{N}_++1)^{1/2}\xi \Vert \Vert \mathcal{K}^{1/2}\xi \Vert \,. \end{aligned} \end{aligned}$$

Combining the bounds for $\mathcal{E}_{31}^K$ and $\mathcal{E}_{32}^K$ , we conclude that, if $\alpha > 1$,

127 $$\begin{aligned} \begin{aligned} | \langle \xi , \text {G}_{23} \xi \rangle | \le C N^{1/2-\alpha } \Vert (\mathcal{N}_+ + 1)^{1/2} \xi \Vert \Vert \mathcal{H}_N^{1/2} \xi \Vert +CN^{1-\alpha }\Vert (\mathcal{N}_+ + 1)^{1/2} \xi \Vert ^2 \end{aligned} \end{aligned}$$

To bound $\text {G}_{24}$ in (117), we use (108), the bounds (28) and $\Vert \eta \Vert ^2_{H_1} \le C N$, and the commutator (14):

$$\begin{aligned} \begin{aligned} |\langle&\xi ,\text {G}_{24}\xi \rangle | \\&\le C \int _0^1 ds \sum _{p \in \varLambda ^*_+} p^2 \eta _p^2 \Vert (\mathcal{N}_++1)^{1/2}\xi \Vert \Vert (\mathcal{N}_++1)^{-1/2} d_p^{(s)}b^*_{p}\xi \Vert \\&\le C \Vert (\mathcal{N}_++1)^{1/2}\xi \Vert \sum _{p \in \varLambda ^*_+} p^2 \eta _p^2 \left[ |\eta _p| \Vert (\mathcal{N}_+ + 1)^{1/2} \xi \Vert +N^{-1} \Vert \eta \Vert \Vert b_p b_p^* (\mathcal{N}_+ +1)^{1/2} \xi \Vert \right] \\&\le C N^{-\alpha } \Vert (\mathcal{N}_+ + 1)^{1/2} \xi \Vert ^2 \,. \end{aligned} \end{aligned}$$

Together with (117), (125), (126) and (127), this implies that

$$\begin{aligned} \text {G}_2 = - \sum _{p \in \varLambda ^*_+} p^2 \eta _p^2 \, \frac{\mathcal{N}_+ + 1}{N} \frac{N-\mathcal{N}_+}{N}+ \mathcal{E}_4^K \end{aligned}$$

with

128 $$\begin{aligned} | \langle \xi , \mathcal{E}_4^K \xi \rangle | \le C N^{1/2 -\alpha } \Vert \mathcal{H}_N^{1/2}\xi \Vert \Vert (\mathcal{N}_++1)^{1/2}\xi \Vert + C N^{1-\alpha } \Vert (\mathcal{N}_++1)^{1/2}\xi \Vert ^2\,. \end{aligned}$$

Combining (115), (116) and (128), we obtain (112) and (113). $\square$

In the next proposition, we consider the conjugation of the operator

$$\begin{aligned} \begin{aligned} \mathcal{L}^{(2,V)}_{N} =\;&N\sum _{p \in \varLambda _+^*} \widehat{V} (p/e^N) \left[ b_p^* b_p - \frac{1}{N} a_p^* a_p \right] + \frac{N}{2} \sum _{p \in \varLambda ^*_+} \widehat{V} (p/e^N) \left[ b_p^* b_{-p}^* + b_p b_{-p} \right] \end{aligned} \end{aligned}$$

#### Proposition 14

Under the assumptions of Proposition 1, there is a constant $C > 0$ such that

129 $$\begin{aligned} \begin{aligned} e^{-B} \mathcal{L}^{(2,V)}_N e^{B} =\;&N\sum _{p \in \varLambda ^*_+} \widehat{V} (p/e^N)\eta _p \Big (\frac{N-\mathcal{N}_+}{N}\Big )\Big (\frac{N-\mathcal{N}_+-1}{N}\Big )\\&+ N\sum _{p \in \varLambda _+^*} \widehat{V} (p/e^N) a^*_pa_p\left( 1-\frac{\mathcal{N}_+}{N}\right) \\&+ \frac{N}{2}\sum _{p \in \varLambda ^*_+} \widehat{V} (p/e^N) \big ( b_p b_{-p}+ b_{-p}^* b_p^*\big ) +\mathcal{E}_{N}^{(V)} \end{aligned} \end{aligned}$$

where

130 $$\begin{aligned} \begin{aligned} | \langle \xi , \mathcal{E}_{N}^{(V)} \xi \rangle | \le C N^{1/2 -\alpha } \Vert \mathcal{H}_N^{1/2}\xi \Vert \Vert (\mathcal{N}_++1)^{1/2}\xi \Vert + C N^{1-\alpha } \Vert (\mathcal{N}_++1)^{1/2}\xi \Vert ^2\,. \end{aligned} \end{aligned}$$

for any $\alpha > 1$, $\xi \in \mathcal{F}^{\le N}_+$ and $N \in {\mathbb {N}}$ large enough.

#### Proof

We write

131 $$\begin{aligned} \begin{aligned} e^{-B} \mathcal{L}^{(2,V)}_{N} e^{B} =\;&N\sum _{p \in \varLambda ^*_+} \widehat{V} (p/e^N) e^{-B} b_p^* b_p e^{B} - \sum _{p \in \varLambda ^*_+} \widehat{V} (p/e^N) e^{-B} a_p^* a_p e^{B} \\&+ \frac{N}{2} \sum _{p \in \varLambda ^*_+} \widehat{V} (p/e^N) e^{-B} \big [ b_p b_{-p} + b_p^* b_{-p}^* \big ] e^{B} \\ =: \;&\text {F}_1 + \text {F}_2 +\text {F}_3\,. \end{aligned} \end{aligned}$$

With (106), we find

$$\begin{aligned} \begin{aligned} \text {F}_1 =\;&N\sum _{p \in \varLambda _+^*} \widehat{V} (p/e^N) \big [ \gamma _p b_p^* + \sigma _p b_{-p} \big ] \big [ \gamma _p b_p + \sigma _p b_{-p}^*] \\&+N\sum _{ p\in \varLambda _+^*} \widehat{V} (p/e^N) \big [ (\gamma _p b_p^* + \sigma _p b_{-p}) d_p+ d_p^*(\gamma _p b_p + \sigma _p b_{-p}^*)+ d_p^* d_p\big ] \end{aligned} \end{aligned}$$

where $\gamma _p = \cosh \eta _p$, $\sigma _p = \sinh \eta _p$ and the operators $d_p$ are defined in (105). Using $|1-\gamma _p | \le \eta _p^2$, $|\sigma _p| \le C |\eta _p|$ and using Lemma 4 for the terms on the second line, we find

132 $$\begin{aligned} \text {F}_1 = N\sum _{p \in \varLambda _+^*} \widehat{V} (p/e^N) b_p^* b_p + \mathcal{E}_1^V \end{aligned}$$

with $$\pm \mathcal{E}_1^V \le C N^{1-\alpha } (\mathcal{N}_+ + 1)$$.

Let us now consider the second contribution on the r.h.s. of (131). We find

133 $$\begin{aligned} -\text {F}_2 = \sum _{p \in \varLambda ^*_+} \widehat{V} (p/e^N) a_p^* a_p + \mathcal{E}_2^V \end{aligned}$$

with

$$\begin{aligned} \mathcal{E}_2^V = \sum _{p \in \varLambda ^*_+} \widehat{V} (p/e^N)\int _0^1 e^{-sB}(\eta _pb_{-p}b_p + \text{ h.c.}) e^{s B}ds. \end{aligned}$$

With Lemma 2, we easily find $\pm \mathcal{E}_2^V \le C N^{-\alpha } (\mathcal{N}_+ + 1)$.

Finally, we consider the last term on the r.h.s. of (131). With (106), we obtain

134 $$\begin{aligned} \begin{aligned} \text {F}_3 =\;&\frac{N}{2} \sum _{p \in \varLambda ^*_+} \widehat{V} (p/e^N) \left[ \gamma _p b_p + \sigma _p b_{-p}^* \right] \left[ \gamma _p b_{-p} + \sigma _p b_p^* \right] +\text{ h.c. }\\&+ \frac{N}{2} \sum _{p \in \varLambda ^*_+} \widehat{V} (p/e^N) \, \left[ (\gamma _p b_p+ \sigma _p b^*_{-p}) \, d_{-p} + d_p\, (\gamma _p b_{-p} + \sigma _p b^*_{p}) \right] + \text{ h.c. }\\&+\frac{N}{2} \sum _{p \in \varLambda ^*_+} \widehat{V} (p/e^N) d_p d_{-p} + \text{ h.c. }\\ =:&\,\text {F}_{31} + \text {F}_{32}+\text {F}_{33}\,. \end{aligned} \end{aligned}$$

Using $|1-\gamma _p| \le C \eta _p^2$, $|\sigma _p| \le C |\eta _p|$, we obtain

135 $$\begin{aligned} \begin{aligned} \text {F}_{31} = \;&\frac{N}{2}\sum _{p \in \varLambda ^*_+} \widehat{V} (p/e^N) \big ( b_p b_{-p}+ b_{-p}^* b_p^*\big ) +N\sum _{p \in \varLambda ^*_+} \widehat{V} (p/e^N) \eta _p \frac{N-\mathcal{N}_+}{N} + \mathcal{E}^V_3 \end{aligned} \end{aligned}$$

with $\pm \mathcal{E}_3^V \le C N^{1-\alpha }(\mathcal{N}_++1)$. As for $\text {F}_{32}$ in (134), we divide it into four parts

136 $$\begin{aligned} \begin{aligned} \text {F}_{32} = \;&\frac{N}{2} \sum _{p \in \varLambda ^*_+} \widehat{V} (p/e^N) \, \left[ ( \gamma _p b_p+ \sigma _p b^*_{-p}) \, d_{-p} + d_p\, (\gamma _p b_{-p} + \sigma _p b^*_{p}) \right] +\text{ h.c. }\\ =: \;&\text {F}_{321}+\text {F}_{322}+\text {F}_{323}+\text {F}_{324} \,. \end{aligned} \end{aligned}$$

We start with $\text {F}_{321}$, which we write as

$$\begin{aligned} \text {F}_{321} = - N\sum _{p \in \varLambda ^*_+} \widehat{V} (p/e^N) \eta _p \, \left( \frac{N-\mathcal{N}_+}{N}\right) \left( \frac{\mathcal{N}_+ +1}{N}\right) +\mathcal{E}_4^V \end{aligned}$$

where $$\mathcal{E}_4^V = \mathcal{E}_{41}^V + \mathcal{E}_{42}^V + \mathcal{E}_{43}^V + \text{ h.c. }$$, with

$$\begin{aligned} \begin{aligned} \mathcal{E}^V_{41} = \;&\frac{N}{2} \sum _{p \in \varLambda ^*_+} \widehat{V} (p/e^N) \, (\gamma _p - 1) b_p d_{-p} \, , \qquad \mathcal{E}_{42}^V = \frac{N}{2} \sum _{p \in \varLambda _+^*} \widehat{V} (p/e^N) b_p \overline{d}_{-p} \\ \mathcal{E}_{43}^V = \;&- \frac{N}{2} \sum _{p \in \varLambda ^*_+} \widehat{V} (p/e^N) \eta _p \frac{\mathcal{N}_+ + 1}{N} (b_p^* b_p - N^{-1} a_p^* a_p ) \end{aligned} \end{aligned}$$

and with the notation $\overline{d}_{-p} = d_{-p} + N^{-1} \eta _p \, \mathcal{N}_+ b_p^*$. Since $|\gamma _p - 1| \le C \eta _p^2$, $\Vert \eta \Vert _\infty \le C N^{-\alpha }$, we find easily with (108) that

$$\begin{aligned} \begin{aligned} |\langle \xi , \mathcal{E}_{41}^V \xi \rangle |&\le C N^{1-3\alpha }\Vert (\mathcal{N}_+ + 1)^{1/2} \xi \Vert ^2 \,. \end{aligned} \end{aligned}$$

Moreover

$$\begin{aligned} |\langle \xi , \mathcal{E}_{43}^V \xi \rangle | \le C N \sum _{p \in \varLambda ^*_+} \eta _p \Vert a_p \xi \Vert ^2 \le C N^{1-\alpha } \Vert \mathcal{N}_+^{1/2} \xi \Vert ^2\,. \end{aligned}$$

As for $\mathcal{E}_{42}^V$, we switch to position space and we use (110). We obtain

$$\begin{aligned} \begin{aligned} |\langle \xi , \mathcal{E}_{42}^V \xi \rangle |&\le CN \int _{\varLambda ^2} dx dy \, e^{2N} V(e^N(x-y)) \Vert (\mathcal{N}_+ + 1)^{1/2} \xi \Vert \Vert (\mathcal{N}_+ + 1)^{-1/2} {\check{a}}_x \check{\overline{d}}_y \xi \Vert \\&\le C N^{1-\alpha } \int _{\varLambda ^2} dx dy \, e^{2N} V(e^N(x-y)) \Vert (\mathcal{N}_+ + 1)^{1/2} \xi \Vert \\&\quad \times \Big [ \Vert (\mathcal{N}_+ + 1)^{1/2} \xi \Vert + \Vert {\check{a}}_x \xi \Vert +\Vert {\check{a}}_y \xi \Vert + N^{-1/2}\Vert {\check{a}}_x {\check{a}}_y \xi \Vert \Big ] \\&\le C N^{1-\alpha } \Vert (\mathcal{N}_+ + 1)^{1/2} \xi \Vert ^2 + C N^{1/2-\alpha } \Vert (\mathcal{N}_+ + 1)^{1/2} \xi \Vert \Vert \mathcal{V}_N^{1/2} \xi \Vert \,. \end{aligned} \end{aligned}$$

We conclude that

$$\begin{aligned} \begin{aligned} |\langle \xi , \mathcal{E}^V_4 \xi \rangle | \le C N^{1/2-\alpha } \Vert (\mathcal{N}_+ + 1)^{1/2} \xi \Vert \Vert \mathcal{V}_N^{1/2} \xi \Vert + C N^{1-\alpha } \Vert (\mathcal{N}_+ + 1)^{1/2} \xi \Vert ^2. \end{aligned} \end{aligned}$$

To bound the term $\text {F}_{322}$ in (136), we use (108) and $|\sigma _p|\le C|\eta _p|$; we obtain

$$\begin{aligned} \begin{aligned} |\langle \xi ,\text {F}_{322} \xi \rangle | \le \;&C N \sum _{p \in \varLambda ^*_+} |\eta _p| \Vert b_{-p} \xi \Vert \left[ |\eta _p| \Vert (\mathcal{N}_+ + 1)^{1/2} \xi \Vert + \Vert \eta \Vert \Vert b_{-p} \xi \Vert \right] \\ \le \;&C N^{1-2\alpha } \Vert (\mathcal{N}_+ + 1)^{1/2} \xi \Vert ^2 \,. \end{aligned} \end{aligned}$$

Let us now consider the term $\text {F}_{323}$ on the r.h.s. of (136). We write $\text {F}_{323} = \mathcal{E}_{51}^V + \mathcal{E}_{52}^V + \text{ h.c. }$, with

$$\begin{aligned} \begin{aligned} \mathcal{E}_{51}^V = \frac{N}{2} \sum _{p \in \varLambda ^*_+} \widehat{V} (p/e^N) \, (\gamma _p-1) \, d_p b_{-p} \, , \qquad \mathcal{E}_{52}^V = \frac{N}{2} \sum _{p \in \varLambda ^*_+} \widehat{V} (p/e^N) \, d_p b_{-p} \,. \end{aligned} \end{aligned}$$

With $|\gamma _p - 1| \le C \eta _p^2$ and (108) we obtain

$$\begin{aligned} |\langle \xi , \mathcal{E}_{51}^V \xi \rangle | \le C N\sum _{p \in \varLambda ^*_+} \eta _p^2 \,\Vert d^*_p \xi \Vert \Vert a_p \xi \Vert \le C N^{1-3\alpha } \Vert (\mathcal{N}_+ + 1)^{1/2} \xi \Vert ^2 \,. \end{aligned}$$

We find, switching to position space and using (109),

$$\begin{aligned} \begin{aligned}&|\langle \xi , \mathcal{E}_{52}^V \xi \rangle |\\&\le CN \int _{\varLambda ^2} dx dy \, e^{2N} V(e^N(x-y)) \Vert (\mathcal{N}_+ + 1)^{1/2} \xi \Vert \Vert (\mathcal{N}_+ +1)^{-1/2} {\check{d}}_x {\check{a}}_y \xi \Vert \\&\le CN^{1-\alpha } \Vert (\mathcal{N}_+ + 1)^{1/2} \xi \Vert \int _{\varLambda ^2} dx dy \, e^{2N} V(e^N(x-y)) \left[ \Vert {\check{a}}_y \xi \Vert + N^{-1/2}\Vert {\check{a}}_x {\check{a}}_y \xi \Vert \right] \\&\le C N^{1-\alpha } \Vert (\mathcal{N}_+ + 1)^{1/2} \xi \Vert ^2 + C N^{1/2-\alpha }\Vert (\mathcal{N}_+ + 1)^{1/2} \xi \Vert \Vert \mathcal{V}_N^{1/2} \xi \Vert \,. \end{aligned} \end{aligned}$$

Hence,

$$\begin{aligned} | \langle \xi , \text {F}_{323} \xi \rangle | \le C N^{1-\alpha } \Vert (\mathcal{N}_+ + 1)^{1/2} \xi \Vert ^2 + C N^{1/2-\alpha }\Vert (\mathcal{N}_+ + 1)^{1/2} \xi \Vert \Vert \mathcal{V}_N^{1/2} \xi \Vert \end{aligned}$$

To estimate the term $\text {F}_{324}$ in (136) we use (108) and the bound

$$\begin{aligned} \begin{aligned} \sum _{ p\in \varLambda _+^*}|\widehat{V}(p/e^N)||\eta _p|&\le C \sum _{ \begin{array}{c} p\in \varLambda _+^*,\, |p|\le e^N \end{array}} \frac{1}{p^2} + C\sum _{ \begin{array}{c} p\in \varLambda _+^*,\, |p|> e^N \end{array}} \frac{|\widehat{V}(p/e^N)|}{p^2} \\&\le C N + C \Bigg ( \sum _{ \begin{array}{c} p\in \varLambda _+^* \end{array}} |\widehat{V}(p/e^N)|^2 \Bigg )^{1/2} \Bigg ( \sum _{ \begin{array}{c} p\in \varLambda _+^*,\, |p|> e^N \end{array}} \frac{1}{p^4} \Bigg )^{1/2} \\&\le C N \end{aligned}\end{aligned}$$

We find

$$\begin{aligned} \begin{aligned} |\langle \xi , \text {F}_{324} \xi \rangle |&\le \; CN\sum _{p \in \varLambda ^*_+} \big |\widehat{V} (p/e^N) \big ||\eta _p|\Vert (\mathcal{N}_++1)^{1/2}\xi \Vert \Vert (\mathcal{N}_++1)^{-1/2} d_p\, b^*_{p}\xi \Vert \\&\le CN\sum _{p \in \varLambda ^*_+} \big |\widehat{V} (p/e^N) \big ||\eta _p|\Vert (\mathcal{N}_++1)^{1/2}\xi \Vert \\&\quad \times \left[ |\eta _p| \Vert (\mathcal{N}_+ + 1)^{1/2} \xi \Vert + N^{-1}\Vert \eta \Vert \Vert b_p b^*_p (\mathcal{N}_+ + 1)^{1/2} \xi \Vert \right] \\&\le CN\sum _{p \in \varLambda ^*_+} \big |\widehat{V} (p/e^N) \big ||\eta _p|\Vert (\mathcal{N}_++1)^{1/2}\xi \Vert \\&\quad \times \left[ |\eta _p| \Vert (\mathcal{N}_+ + 1)^{1/2} \xi \Vert + N^{-1}\Vert \eta \Vert \Vert (\mathcal{N}_+ + 1)^{1/2} \xi \Vert + \Vert \eta \Vert \Vert a_p \xi \Vert \right] \\&\le \; C N^{1-\alpha }\Vert (\mathcal{N}_++1)^{1/2}\xi \Vert ^{2}\,. \end{aligned} \end{aligned}$$

Combining the last bounds, we arrive at

$$\begin{aligned} \text {F}_{32} = N\sum _{p \in \varLambda ^*_+} \widehat{V} (p/e^N) \eta _p \left( \frac{N-\mathcal{N}_+}{N} \right) \left( \frac{-\mathcal{N}_+ -1}{N} \right) + \mathcal{E}_6^V \end{aligned}$$

with

137 $$\begin{aligned} | \langle \xi , \mathcal{E}^V_6 \xi \rangle | \le C N^{1-\alpha } \Vert (\mathcal{N}_+ + 1)^{1/2} \xi \Vert ^2 + C N^{1/2-\alpha }\Vert (\mathcal{N}_+ + 1)^{1/2} \xi \Vert \Vert \mathcal{V}_N^{1/2} \xi \Vert \,. \end{aligned}$$

To control the last contribution $\text {F}_{33}$ in (134), we switch to position space. With (111) and (25) we obtain

$$\begin{aligned} \begin{aligned} |\langle \xi , \text {F}_{33} \xi \rangle |&\le CN \Vert (\mathcal{N}_+ + 1)^{1/2} \xi \Vert \int _{\varLambda ^2} dx dy \, e^{2N} V(e^N(x-y)) \Vert (\mathcal{N}_+ + 1)^{-1/2} {\check{d}}_x {\check{d}}_y \xi \Vert \\&\le C N^{1-\alpha } \Vert (\mathcal{N}_+ + 1)^{1/2} \xi \Vert ^2 + C N^{1/2-2\alpha } \Vert (\mathcal{N}_++1)^{1/2} \xi \Vert \Vert \mathcal{V}_N^{1/2} \xi \Vert \,. \end{aligned} \end{aligned}$$

The last equation, combined with (134), (135) and (137), implies that

$$\begin{aligned} \begin{aligned} \text {F}_3 = \;&\frac{N}{2} \sum _{p \in \varLambda _+^*} \widehat{V} (p/e^N) (b_p b_{-p} + b^*_{-p} b^*_p) \\&+ N\sum _{p \in \varLambda ^*_+} \widehat{V} (p/e^N) \eta _p \left( \frac{N-\mathcal{N}_+}{N} \right) \left( \frac{N-\mathcal{N}_+ - 1}{N} \right) + \mathcal{E}_7^V \end{aligned} \end{aligned}$$

with

$$\begin{aligned} | \langle \xi , \mathcal{E}^V_7 \xi \rangle | \le C N^{1-\alpha } \Vert (\mathcal{N}_+ + 1)^{1/2} \xi \Vert ^2 + C N^{1/2-\alpha }\Vert (\mathcal{N}_+ + 1)^{1/2} \xi \Vert \Vert \mathcal{V}_N^{1/2} \xi \Vert \,. \end{aligned}$$

Together with (132) and with (133), and recalling that $b_p^* b_p -N^{-1} a_p^* a_p = a_p^* a_p (1- \mathcal{N}_+ / N)$, we obtain (129) with (130). $\square$

### Appendix A.4: Analysis of $\mathcal{G}_{N,\alpha }^{(3)}=e^{-B}\mathcal{L}^{(3)}_N e^{B}$

We consider here the conjugation of the cubic term $\mathcal{L}_N^{(3)}$, defined in (13).

#### Proposition 15

Under the assumptions of Proposition 1, there exists a constant $C > 0$ such that

$$\begin{aligned} \mathcal{G}_{N,\alpha }^{(3)} = e^{-B}\mathcal{L}^{(3)}_N e^{B} = \sqrt{N} \sum _{p,q \in \varLambda ^*_+ : p + q \not = 0} \widehat{V} (p/e^N) \left[ b_{p+q}^* a_{-p}^* a_q + \text{ h.c. }\right] + \mathcal{E}^{(3)}_{N} \end{aligned}$$

where

138 $$\begin{aligned} | \langle \xi , \mathcal{E}_{N}^{(3)} \xi \rangle | \le C N^{1/2-\alpha }\Vert (\mathcal{N}_+ + 1)^{1/2} \xi \Vert \Vert \mathcal{V}_N^{1/2} \xi \Vert + C N^{1-\alpha } \Vert (\mathcal{N}_+ + 1)^{1/2} \xi \Vert ^2 \end{aligned}$$

for any $\alpha > 1$ and $N \in {\mathbb {N}}$ large enough.

#### Proof

This proof is similar to the proof of [4, Prop. 7.5]. Expanding $e^{-B} a_{-p}^* a_q e^B$, we arrive at

139 $$\begin{aligned} \begin{aligned} \mathcal{E}^{(3)}_{N}&= \sqrt{N} \sum _{p,q \in \varLambda ^*_+ : p+q \not = 0} \widehat{V} (p/e^N) \big ((\gamma _{p+q}-1) b^*_{p+q} + \sigma _{p+q} b_{-p-q} + d_{p+q}^* \big ) \, a_{-p}^* a_q \\&\quad + \sqrt{N} \sum _{p,q \in \varLambda _+^* , p+q \not = 0} \widehat{V} (p/e^N) \eta _p \, e^{-B}b^*_{p+q}e^{B} \int _0^1 ds\, e^{-sB} b_{p} b_{q} e^{sB}\\&\quad + \sqrt{N} \sum _{p,q \in \varLambda _+^* , p+q \not = 0} \widehat{V} (p/e^N) \eta _q \, e^{-B} b^*_{p+q}e^{B} \int _0^1 ds\, e^{-sB}b_{-p}^*b^*_{-q} e^{sB} \\&\quad + \text{ h.c. }\\&=: \; \mathcal{E}^{(3)}_1 + \mathcal{E}_2^{(3)} + \mathcal{E}_3^{(3)} + \text{ h.c. }\end{aligned} \end{aligned}$$

where, as usual, $\gamma _p = \cosh \eta (p)$, $\sigma _p = \sinh \eta (p)$ and $d_p$ is as in (105). We consider $\mathcal{E}_1^{(3)}$. To this end, we write

$$\begin{aligned} \begin{aligned} \mathcal{E}^{(3)}_1 =\;&\sqrt{N} \sum _{p,q \in \varLambda ^*_+ : p+q \not = 0} \widehat{V} (p/e^N) \big ((\gamma _{p+q}-1) b^*_{p+q} + \sigma _{p+q} b_{-p-q} + d_{p+q}^* \big ) \, a_{-p}^* a_q \\ =:&\; \mathcal{E}^{(3)}_{11} + \mathcal{E}^{(3)}_{12} +\mathcal{E}^{(3)}_{13} \,. \end{aligned}\end{aligned}$$

Since $|\gamma _{p+q}-1|\le |\eta _{p+q}|^2$ and $\Vert \eta \Vert \le C N^{-\alpha }$, we find

140 $$\begin{aligned} |\langle \xi , \mathcal{E}^{(3)}_{11} \xi \rangle | \le CN \Vert \eta \Vert ^2 \Vert (\mathcal{N}_++1)^{1/2} \xi \Vert ^2 \le C N^{1-2\alpha } \Vert (\mathcal{N}_+ + 1)^{1/2} \xi \Vert ^2 \,. \end{aligned}$$

As for $\mathcal{E}^{(3)}_{12}$, we commute $a^*_{-p}$ through $b_{-p-q}$ (recall $q \not = 0$). With $|\sigma _{p+q}| \le C |\eta _{p+q}|$, we obtain

141 $$\begin{aligned} |\langle \xi , \mathcal{E}^{(3)}_{12} \xi \rangle | \le C N^{1-\alpha } \Vert (\mathcal{N}_++1)^{1/2} \xi \Vert ^2 \,. \end{aligned}$$

We decompose now $\mathcal{E}^{(3)}_{13} = \mathcal{E}^{(3)}_{131} + \mathcal{E}^{(3)}_{132}$, with

$$\begin{aligned} \begin{aligned} \mathcal{E}^{(3)}_{131} =\;&\sqrt{N} \sum _{p,q \in \varLambda ^*_+ : p+q \not = 0} \widehat{V} (p/e^N) \, {\bar{d}}^*_{p+q} a^*_{-p} a_q \\ \mathcal{E}^{(3)}_{132} = \;&-\frac{(\mathcal{N}_++1)}{N}\sqrt{N} \sum _{p,q \in \varLambda ^*_+ : p+q \not = 0} \widehat{V} (p/e^N) \eta _{p+q} \, b_{-p-q} a^*_{-p} a_q \,. \end{aligned}\end{aligned}$$

where we defined $d^*_{p+q}= \overline{d}^*_{p+q} - \frac{(\mathcal{N}_++1)}{N}\, \eta _{p+q} b_{-p-q}$. The term $\mathcal{E}^{(3)}_{132}$ is estimated similarly to $\mathcal{E}_{12}^{(3)}$, moving $a_{-p}^*$ to the left of $b_{-p-q}$; we find $\pm \mathcal{E}^{(3)}_{132} \le C N^{1-\alpha } (\mathcal{N}_+ + 1)$. We bound $\mathcal{E}^{(3)}_{131}$ in position space. We find

$$\begin{aligned}\begin{aligned}&|\langle \xi , \mathcal{E}^{(3)}_{131} \xi \rangle |\\&\quad \le N^{1/2}\int _{\varLambda ^2} dx dy \,e^{2N}V(e^N(x-y)) \Vert {\check{a}}_x \xi \Vert \Vert {\check{a}}_y \check{{\bar{d}}}_x \xi \Vert \\&\quad \le CN^{1/2-\alpha } \int _{\varLambda ^2} dx dy \,e^{2N} V(e^N(x-y))\Vert {\check{a}}_x \xi \Vert \\&\qquad \times \big [ \,\Vert (\mathcal{N}_++1)\xi \Vert + N^{-1} \Vert {\check{a}}_x (\mathcal{N}_+ + 1)^{1/2} \xi \Vert +\Vert \eta \Vert \Vert {\check{a}}_y (\mathcal{N}_+ + 1)^{1/2} \xi \Vert + \Vert {\check{a}}_x {\check{a}}_y \xi \Vert \big ] \\&\quad \le C N^{1-\alpha } \Vert (\mathcal{N}_+ + 1)^{1/2} \xi \Vert ^2+CN^{1/2-\alpha }\Vert (\mathcal{N}_+ + 1)^{1/2} \xi \Vert \Vert \mathcal{V}_N^{1/2} \xi \Vert \,. \end{aligned}\end{aligned}$$

With (140) and (141) we obtain

142 $$\begin{aligned} |\langle \xi , \mathcal{E}^{(3)}_1\xi \rangle | \le C N^{1/2-\alpha } \Vert \mathcal{V}_N^{1/2}\xi \Vert \Vert (\mathcal{N}_+ +1)^{1/2} \xi \Vert + C N^{1-\alpha } \Vert (\mathcal{N}_+ +1)^{1/2} \xi \Vert ^2 \,. \end{aligned}$$

Next, we focus on $\mathcal{E}^{(3)}_2$, defined in (139). With Eq. (106), we find

143 $$\begin{aligned} \begin{aligned} \mathcal{E}^{(3)}_2 =\;&\sqrt{N}\sum _{p,q \in \varLambda _+^* , p+q \not = 0} \widehat{V} (p/e^N) \eta _p \, e^{-B}b^*_{p+q}e^{B}\\&\,\times \int _0^1 ds\, \big ( \gamma ^{(s)}_p \gamma ^{(s)}_{q} b_{p} b_q + \sigma ^{(s)}_p \sigma ^{(s)}_{q}b^*_{-p} b^*_{-q} +\gamma ^{(s)}_p \sigma ^{(s)}_{q} b^*_{-q} b_{p} + \sigma ^{(s)}_p \gamma ^{(s)}_{q} b^*_{-p} b_{q} \big ) \\&\,+\sqrt{N} \sum _{p,q \in \varLambda _+^* , p+q \not = 0} \widehat{V} (p/e^N) \eta _p \, e^{-B}b^*_{p+q}e^{B} \int _0^1 ds\, \gamma ^{(s)}_p \sigma ^{(s)}_{q} [b_{p}, b^*_{-q}] \\&\,+ \sqrt{N} \sum _{p,q \in \varLambda _+^* , p+q \not = 0} \widehat{V} (p/e^N) \eta _p \, e^{-B}b^*_{p+q}e^{B}\\&\,\times \int _0^1 ds\, \Big [ d^{(s)}_p \big ( \gamma ^{(s)}_{q} b_{q} + \sigma ^{(s)}_{q} b^*_{-q} \big ) + \big ( \gamma ^{(s)}_p b_{p} + \sigma ^{(s)}_p b^*_{-p} \big ) d^{(s)}_{q} + d^{(s)}_{p} d^{(s)}_{q} \Big ]\\ =:&\; \mathcal{E}^{(3)}_{21} + \mathcal{E}^{(3)}_{22} + \mathcal{E}^{(3)}_{23} \end{aligned}\end{aligned}$$

with $\gamma ^{(s)}_p = \cosh (s \eta _p)$, $\sigma ^{(s)}_p = \sinh (s \eta _p)$ and $d^{(s)}_p$ defined as in (105), with $\eta$ replaced by $s \eta$. With Lemma 2, we get

144 $$\begin{aligned} |\langle \xi , \mathcal{E}^{(3)}_{21} \xi \rangle | \le \; C N^{1-\alpha } \Vert (\mathcal{N}_++1)^{1/2}\xi \Vert ^2 \,. \end{aligned}$$

Since $$[b_{p},b^*_{-q}] = - a^*_{-q} a_{p} /N $$ for $$p \ne -q$$, we find

145 $$\begin{aligned} |\langle \xi , \mathcal{E}^{(3)}_{22} \xi \rangle | \le CN^{-2\alpha } \Vert (\mathcal{N}_+ + 1)^{1/2} \xi \Vert ^2 \,. \end{aligned}$$

As for the third term on the r.h.s. of (143), we switch to position space. We find

$$\begin{aligned} \begin{aligned} \mathcal{E}^{(3)}_{23} =\;&\sqrt{N} \int _{\varLambda ^3} dx dy dz\, e^{2N} V(e^N(x-z)) {\check{\eta }} (y-z) \,e^{-B} {\check{b}}^*_x e^{B}\\&\times \int _0^1 ds\, \Big [ {\check{d}}^{(s)}_y \big ( b({\check{\gamma }}^{(s)}_{x}) +b^*({\check{\sigma }}^{(s)}_{x}) \big ) + \big ( b({\check{\gamma }}^{(s)}_{y}) +b^*({\check{\sigma }}^{(s)}_{y}) \big ) \check{ d}^{(s)}_x + {\check{d}}^{(s)}_y {\check{d}}^{(s)}_x \Big ]\,. \end{aligned}\end{aligned}$$

Using the bounds (109), (110), (111) and Lemma 2 we arrive at

$$\begin{aligned}\begin{aligned}&|\langle \xi , \mathcal{E}^{(3)}_{23} \xi \rangle | \\&\le C \sqrt{N} \int _{\varLambda ^3} dx dy dz \, e^{2N} V(e^N(x-z)) | {\check{\eta }} (y-z)| \Vert {\check{b}}_x e^{B} \xi \Vert \int _0^1 ds \\&\quad \times \Big [ \Vert {\check{d}}^{(s)}_y \big ( {\check{b}}_{x} + b({\check{r}}^{(s)}_x) + b^*({\check{\sigma }}^{(s)}_x)\big )\xi \Vert +\Vert \big ( {\check{b}}_{y} + b({\check{r}}^{(s)}_y) + b^*({\check{\sigma }}^{(s)}_y)\big ) {\check{d}}^{(s)}_x \xi \Vert + \Vert {\check{d}}^{(s)}_x {\check{d}}^{(s)}_y \xi \Vert \Big ] \\&\le C \sqrt{N} \int _{\varLambda ^3} dx dy dz \, e^{2N} V(e^N(x-z)) | {\check{\eta }} (y-z)| \Vert {\check{b}}_x e^{B} \xi \Vert \Big [ N^{-1} |{\check{\eta }} (x-y)| \Vert (\mathcal{N}_+ +1) \xi \Vert \\&\quad + \Vert \eta \Vert \Vert {\check{b}}_{x} {\check{b}}_{y} \xi \Vert + \Vert \eta \Vert \Vert (\mathcal{N}_++1) \xi \Vert + \Vert \eta \Vert \Vert {\check{b}}_x (\mathcal{N}_++1)^{1/2} \xi \Vert + \Vert \eta \Vert \Vert {\check{b}}_y (\mathcal{N}_++1)^{1/2} \xi \Vert \Big ] \\&\le C N^{1-\alpha } \Vert \mathcal{N}_+^{1/2} e^{B} \xi \Vert \Vert (\mathcal{N}_++ 1) \xi \Vert \\&\le C N^{1-\alpha } \Vert (\mathcal{N}_++1)^{1/2} \xi \Vert ^2 \, \end{aligned} \end{aligned}$$

where ${\check{r}}$ indicates the function in $L^2 (\varLambda )$ with Fourier coefficients $r_p =1-\gamma _p$, and the fact that $\Vert {\check{\eta }}\Vert , \Vert {\check{r}}\Vert , \Vert {\check{\sigma }}\Vert \le C N^{-\alpha }$. Combined with (144) and (145), the last bound implies that

146 $$\begin{aligned} \begin{aligned} \pm \mathcal{E}_2^{(3)} \le&C N^{1-\alpha }(\mathcal{N}_+ + 1) \,. \end{aligned}\end{aligned}$$

To bound the last contribution on the r.h.s. of (139), it is convenient to bound (in absolute value) the expectation of its adjoint

$$\begin{aligned} \begin{aligned} \mathcal{E}^{(3)*}_3 =\;&\sqrt{N} \sum _{p,q \in \varLambda _+^* , p+q \not = 0} \widehat{V} (p/e^N) \eta _q \int _0^1 ds\, e^{-sB} b_{-q}e^{sB}\\&\times \big ( \gamma ^{(s)}_p b_{-p} + \sigma ^{(s)}_p b^*_{p} + d^{(s)}_{-p}\big ) \big ( \gamma _{p+q} b_{p+q} + \sigma _{p+q} b^*_{-p-q} + d_{p+q}\big ) \\ =\;&\sqrt{N} \sum _{p,q \in \varLambda _+^* , p+q \not = 0} \widehat{V} (p/e^N) \eta _q \, \int _0^1 ds\,e^{-sB} b_{-q}e^{sB}\\&\times \Big [ \, \gamma ^{(s)}_p \gamma _{p+q} b_{-p} b_{p+q} + \sigma ^{(s)}_p \sigma _{p+q} b^*_{p} b^*_{-p-q} + \gamma ^{(s)}_p \sigma _{p+q} b^*_{-p-q} b_{-p}+ \gamma _{p+q} \sigma ^{(s)}_p b^*_{p} b_{p+q} \\&+ d^{(s)}_{-p} \big ( \gamma _{p+q} b_{p+q} + \sigma _{p+q} b^*_{-p-q}\big ) + \big ( \gamma ^{(s)}_p b_{-p} + \sigma ^{(s)}_p b^*_{p}\big ) d_{p+q} + d^{(s)}_{-p} d_{p+q}\Big ] \\&+\sqrt{N} \sum _{p,q \in \varLambda _+^* , p+q \not = 0} \widehat{V} (p/e^N) \eta _q \, \int _0^1 ds\,e^{-sB} b_{-q}e^{sB} \gamma ^{(s)}_p \sigma _{p+q} [b_{-p},b^*_{-p-q}] \\ =:&\, \mathcal{E}_{31}^{(3)} + \mathcal{E}_{32}^{(3)} \,. \end{aligned} \end{aligned}$$

Since $$q \ne 0$$, $$[b_{-p},b^*_{-p-q}] = - a^*_{-p-q} a_{-p} /N $$. Thus, we can estimate

147 $$\begin{aligned} \begin{aligned} | \langle \xi ,&\mathcal{E}_{32}^{(3)} \xi \rangle | \\&\le CN^{-1/2} \int _0^1 ds \sum _{p,q \in \varLambda _+^* , p+q \not = 0} | \eta _q | | \eta _{p+q}| \, \Vert a_{-p-q}\,e^{-sB} b^*_{-q}e^{sB} \xi \Vert \Vert a_{-p} \xi \Vert \\&\le C \Vert \eta \Vert ^2 \Vert (\mathcal{N}_++1)^{1/2} \xi \Vert ^2 \le CN^{-2\alpha } \Vert (\mathcal{N}_+ + 1)^{1/2} \xi \Vert ^2 \,. \end{aligned}\end{aligned}$$

To bound the expectation of $\mathcal{E}^{(3)}_{31}$, we switch to position space. We find

$$\begin{aligned} \begin{aligned}&|\langle \xi , \mathcal{E}^{(3)}_{31} \xi \rangle | \\&\quad \le N^{1/2}\int _0^1 ds\, \int _{\varLambda ^2} dx dy \, e^{2N} V(e^N(x-y))\Vert b^*({\check{\eta }}_{x}) e^{sB} \xi \Vert \Big [ \Vert {\check{b}}_{x}{\check{b}}_{y} \xi \Vert \\&\qquad + \Vert \eta \Vert \Vert {\check{b}}_x (\mathcal{N}_++1)^{1/2} \xi \Vert + \Vert \eta \Vert \Vert {\check{b}}_y (\mathcal{N}_++1)^{1/2} \xi \Vert + N^{-1}|{{\check{\eta }}}(x-y)| \Vert (\mathcal{N}_++1) \xi \Vert \Big ] \,. \end{aligned} \end{aligned}$$

With Lemma 2, we conclude that

148 $$\begin{aligned} |\langle \xi , \mathcal{E}_{31}^{(3)} \xi \rangle | \le C N^{1/2-\alpha } \Vert \mathcal{V}_N^{1/2}\xi \Vert \Vert (\mathcal{N}_+ +1)^{1/2} \xi \Vert + C N^{1-\alpha } \Vert (\mathcal{N}_+ +1)^{1/2} \xi \Vert ^2 \,. \end{aligned}$$

From (147) and (148) we obtain

$$\begin{aligned} |\langle \xi , \mathcal{E}^{(3)}_3\xi \rangle | \le C N^{1/2-\alpha } \Vert \mathcal{V}_N^{1/2}\xi \Vert \Vert (\mathcal{N}_+ +1)^{1/2} \xi \Vert + C N^{1-\alpha } \Vert (\mathcal{N}_+ +1)^{1/2} \xi \Vert ^2 \, . \end{aligned}$$

Together with (139), (142) and (146), we arrive at (138). $\square$

### Appendix A.5: Analysis of $\mathcal{G}_{N,\alpha }^{(4)}=e^{-B}\mathcal{L}^{(4)}_N e^{B}$

Finally, we consider the conjugation of the quartic term $\mathcal{L}_N^{(4)}$. We define the error operator $\mathcal{E}_N^{(4)}$ through

$$\begin{aligned} \begin{aligned} \mathcal{G}_{N,\alpha }^{(4)} = e^{-B} \mathcal{L}^{(4)}_{N} e^{B} = \;&\mathcal{V}_N + \frac{1}{2} \sum _{\begin{array}{c} q \in \varLambda ^*_+, r\in \varLambda ^* \\ r\ne -q \end{array}} \widehat{V} (r/e^N) \eta _{q+r} \eta _q \left( 1-\frac{\mathcal{N}_+ }{N} \right) \left( 1 - \frac{\mathcal{N}_+ +1}{N} \right) \\&+ \frac{1}{2}\sum _{\begin{array}{c} q \in \varLambda ^*_+, r \in \varLambda ^*: \\ r\ne -q \end{array}} \widehat{V} (r/e^N) \, \eta _{q+r} \left( b_q b_{-q} + b^*_q b^*_{-q} \right) + \mathcal{E}^{(4)}_{N} \end{aligned} \end{aligned}$$

#### Proposition 16

Under the assumptions of Proposition 1 there exists a constant $C > 0$ such that

149 $$\begin{aligned} |\langle \xi , \mathcal{E}^{(4)}_N\xi \rangle | \le C N^{1/2-\alpha } \Vert \mathcal{V}_N^{1/2}\xi \Vert \Vert (\mathcal{N}_+ +1)^{1/2} \xi \Vert + C N^{1-\alpha } \Vert (\mathcal{N}_+ +1)^{1/2} \xi \Vert ^2 \end{aligned}$$

for any $\alpha > 1$, $\xi \in \mathcal{F}^{\le N}_+$ and $N \in {\mathbb {N}}$ large enough.

To show Proposition 16, we use the following lemma, whose proof can be obtained as in [4, Lemma 7.7].

#### Lemma 6

Let $\eta \in \ell ^2 (\varLambda ^*)$ as defined in (27). Then there exists a constant $C > 0$ such that

$$\begin{aligned} \begin{aligned} \Vert (\mathcal{N}_++1)^{n/2} e^{-B}&{\check{b}}_x {\check{b}}_y e^{B}\xi \Vert \\&\le C \Big [\, N \Vert (\mathcal{N}_+ +1)^{n/2} \xi \Vert + \Vert {\check{a}}_y (\mathcal{N}_+ +1)^{(n+1)/2} \xi \Vert \\&\quad + \Vert {\check{a}}_x (\mathcal{N}_+ +1)^{(n+1)/2} \xi \Vert + \Vert {\check{a}}_x {\check{a}}_y (\mathcal{N}_++1)^{n/2} \xi \Vert \Big ] \end{aligned}\end{aligned}$$

for all $$\xi \in \mathcal{F}_+^{\le N}$$, $$n \in {\mathbb {Z}}$$.

#### Proof of Proposition 16

We follow the proof of [4, Prop. 7.6]. We write

$$\begin{aligned} \mathcal{G}^{(4)}_{N,\alpha } = \mathcal{V}_N + W_1 + W_2 + W_3 + W_4 \end{aligned}$$

with

150 $$\begin{aligned} \begin{aligned} \text {W}_1 = \;&\frac{1}{2} \sum _{q \in \varLambda _+^* , r \in \varLambda ^* : r \not = -q} \widehat{V} (r/e^N) \eta _{q+r} \int _0^1 ds \, \big ( e^{-s B} b_{q} b_{-q}\,e^{sB} + \text {h.c.} \big ) \\ \text {W}_2 = \;&\sum _{p,q \in \varLambda _+^* , r \in \varLambda ^* : r \not = p,-q} \widehat{V} (r/e^N)\, \eta _{q+r} \int _0^1 ds \, \big ( e^{-s B} b^*_{p+r} b^*_{q} e^{s B} a^*_{-q-r} a_p + \text {h.c.} \big )\\ \text {W}_3 = \;&\sum _{p,q\in \varLambda ^*_+, r \in \varLambda ^* : r \not = -p -q} \widehat{V} (r/e^N) \eta _{q+r} \eta _p \, \\&\quad \times \int _0^1 ds\,\int _0^s d\tau \, \big (e^{-sB} b^*_{p+r} b^*_q e^{sB} e^{-\tau B} b^*_{-p} b^*_{-q-r} e^{\tau B}+ \text{ h.c. }\big ) \\ \text {W}_4 = \;&\sum _{p,q\in \varLambda ^*_+, r \in \varLambda ^* : r \not = -p -q} \widehat{V} (r/e^N) \, \eta ^2_{q+r} \\&\quad \times \int _0^1 ds\,\int _0^s d\tau \, \big ( e^{-sB} b^*_{p+r} b^*_q e^{sB} e^{-\tau B} b_{p} b_{q+r} e^{\tau B} + \text{ h.c. }\big ) \,. \end{aligned} \end{aligned}$$

Let us first consider the term $\text {W}_1$. With (106), we find

151 $$\begin{aligned} \begin{aligned} \text {W}_1 = \;&\frac{1}{2} \sum _{q \in \varLambda _+^* , r \in \varLambda ^* : r \not = -q} \widehat{V} (r/e^N) \eta _{q+r} \int _0^1 ds (\gamma ^{(s)}_q)^2 (b_q b_{-q} + \text{ h.c.}) \\&+ \frac{1}{2} \sum _{q \in \varLambda _+^* , r \in \varLambda ^* : r \not = -q} \widehat{V} (r/e^N) \eta _{q+r} \int _0^1 ds \, \gamma _q^{(s)} \sigma ^{(s)}_q \big ( [b_q , b_q^*] + \text{ h.c. }\big ) \\&+ \frac{1}{2} \sum _{q \in \varLambda _+^* , r \in \varLambda ^* : r \not = -q} \widehat{V} (r/e^N) \eta _{q+r} \int _0^1 ds \, \gamma _q^{(s)} \big ( b_q d_{-q}^{(s)}+\text{ h.c. }\big ) + \mathcal{E}_{10}^{(4)} \\ =:&\; \text {W}_{11} + \text {W}_{12} + \text {W}_{13} + \mathcal{E}^{(4)}_{10} \end{aligned} \end{aligned}$$

where

152 $$\begin{aligned} \mathcal{E}_{10}^{(4)} = \; \mathcal{E}_{101}^{(4)} + \mathcal{E}_{102}^{(4)} + \mathcal{E}_{103}^{(4)} + \mathcal{E}_{104}^{(4)} + \mathcal{E}_{105}^{(4)} \end{aligned}$$

with

153 $$\begin{aligned} \mathcal{E}^{(4)}_{101}= & {} \frac{1}{2} \sum _{q \in \varLambda _+^* , r \in \varLambda ^* : r \not = -q} \widehat{V} (r/e^N) \eta _{q+r} \int _0^1 ds \Big [ 2 \gamma _q^{(s)} \sigma _q^{(s)} b_q^* b_q + (\sigma _q^{(s)})^2 b_{-q}^* b_q^* +\text{ h.c. }\Big ] \nonumber \\ \mathcal{E}^{(4)}_{102}= & {} \frac{1}{2} \sum _{q \in \varLambda _+^* , r \in \varLambda ^* : r \not = -q} \widehat{V} (r/e^N) \eta _{q+r} \int _0^1 ds \, \sigma _q^{(s)} \big ( b_{-q}^* d_{-q}^{(s)} + \text{ h.c. }\big ) \nonumber \\ \mathcal{E}^{(4)}_{103}= & {} \frac{1}{2} \sum _{q \in \varLambda _+^* , r \in \varLambda ^* : r \not = -q} \widehat{V} (r/e^N) \eta _{q+r} \int _0^1 ds \, \sigma _q^{(s)} \big ( d^{(s)}_q b_q^* + \text{ h.c. }\big ) \nonumber \\ \mathcal{E}^{(4)}_{104}= & {} \frac{1}{2} \sum _{q \in \varLambda _+^* , r \in \varLambda ^* : r \not = -q} \widehat{V} (r/e^N) \eta _{q+r} \int _0^1 ds \, \gamma _q^{(s)} \big ( d^{(s)}_q b_{-q} + \text{ h.c. }\big ) \nonumber \\ \mathcal{E}^{(4)}_{105}= & {} \frac{1}{2} \sum _{q \in \varLambda _+^* , r \in \varLambda ^* : r \not = -q} \widehat{V} (r/e^N) \eta _{q+r} \int _0^1 ds \big ( d^{(s)}_q d^{(s)}_{-q} + \text{ h.c. }\big ) \,. \end{aligned}$$

With

154 $$\begin{aligned} \frac{1}{N}\sup _{q \in \varLambda _+^*} \sum _{r \in \varLambda _+^*} |\widehat{V} (r/e^N)| |\eta _{q+r}| \le C \, < \infty \end{aligned}$$

uniformly in $N \in {\mathbb {N}}$, we can estimate the first term in (153) by

$$\begin{aligned} \begin{aligned}|\langle \xi , \mathcal{E}^{(4)}_{101} \xi \rangle |&\le C N^{1-\alpha }\Vert (\mathcal{N}_+ + 1)^{1/2} \xi \Vert ^2\,. \end{aligned} \end{aligned}$$

Using (154) and (108) we also find

$$\begin{aligned} \begin{aligned} |\langle \xi , \mathcal{E}_{102}^{(4)} \xi \rangle |&\le C N^{1-2\alpha } \Vert (\mathcal{N}_+ + 1)^{1/2} \xi \Vert ^2 \,.\end{aligned} \end{aligned}$$

For the third term in (153) we switch to position space and use (109):

$$\begin{aligned} \begin{aligned} |\langle \xi , \mathcal{E}_{103}^{(4)} \xi \rangle |&\le \frac{1}{2} \int dx dy e^{2N} V(e^N (x-y)) |{{\check{\eta }}}(x-y)| \\&\quad \times \int _0^1 ds \, \Vert (\mathcal{N}+1)^{-1/2} {\check{d}}_y b^*({\check{\sigma }}_x^{(s)})\xi \Vert \Vert (\mathcal{N}+1)^{1/2}\xi \Vert \\&\le C \Vert {{\check{\eta }}} \Vert _\infty \Vert \eta \Vert \int dx dy e^{2N} V(e^N (x-y)) \Vert (\mathcal{N}_++1)^{1/2}\xi \Vert \int _0^1ds \\&\quad \times \Big [ \Vert b^*({\check{\sigma }}^{(s)}_x) \xi \Vert + \frac{1}{N} |{\check{\eta }}^{(s)}(x-y)| \Vert (\mathcal{N}+1)^{1/2}\xi \Vert + \frac{1}{\sqrt{N}} \Vert b^*({\check{\sigma }}^{(s)}_x) {\check{b}}_y \xi \Vert \Big ] \\&\le C N^{1-\alpha }\Vert (\mathcal{N}_++1)^{1/2} \xi \Vert ^2 \,. \end{aligned} \end{aligned}$$

Consider now the fourth term in (153). We write $\mathcal{E}_{104}^{(4)} = \mathcal{E}_{1041}^{(4)} + \mathcal{E}_{1042}^{(4)}$, with

$$\begin{aligned} \begin{aligned} \mathcal{E}_{1041}^{(4)}&= \frac{1}{2} \sum _{q \in \varLambda _+^* , r \in \varLambda ^* : r \not = -q} \widehat{V} (r/e^N) \eta _{q+r} \int _0^1 ds \, (\gamma _q^{(s)} -1) d^{(s)}_q b_{-q} \\ \mathcal{E}_{1042}^{(4)}&= \frac{1}{2} \sum _{q \in \varLambda _+^* , r \in \varLambda ^* : r \not = -q} \widehat{V} (r/e^N) \eta _{q+r} \int _0^1 ds \, d^{(s)}_q b_{-q} \end{aligned} \end{aligned}$$

With $|\gamma _q^{(s)} - 1| \le C |\eta _q|^2$, (154) and $\Vert d^*_q \xi \Vert \le C \Vert \eta \Vert \Vert (\mathcal{N}_++1)^{1/2}\xi \Vert$, we find

$$\begin{aligned}| \langle \xi , \mathcal{E}_{1041}^{(4)} \xi \rangle | \le C N^{1-3\alpha }\Vert (\mathcal{N}_+ + 1)^{1/2} \xi \Vert ^2 \end{aligned}$$

As for $\mathcal{E}_{1042}^{(4)}$, we switch to position space. Using (25) and (109), we obtain

$$\begin{aligned} \begin{aligned}&| \langle \xi , \mathcal{E}_{1042}^{(4)} \xi \rangle | \\&\quad = \Big |\frac{1}{2} \int _0^1 ds \int _{\varLambda ^2} dx dy \,e^{2N} V(e^N(x-y)) {\check{\eta }} (x-y) \langle \xi , {\check{d}}^{(s)}_x {\check{b}}_y \xi \rangle \Big | \\&\quad \le CN \int _0^1 ds \int _{\varLambda ^2} dx dy \,e^{2N} V(e^N(x-y)) \Vert (\mathcal{N}_+ + 1)^{1/2} \xi \Vert \Vert (\mathcal{N}_+ + 1)^{-1/2} {\check{d}}^{(s)}_x {\check{b}}_y \xi \Vert \\&\quad \le C N\Vert \eta \Vert \int _0^1 ds \int _{\varLambda ^2} dx dy \,e^{2N} V(e^N(x-y)) \Vert (\mathcal{N}_+ + 1)^{1/2} \xi \Vert \\&\qquad \times N^{-1}\left[ \Vert {\check{a}}_y \mathcal{N}_+ \xi \Vert + \Vert {\check{a}}_x {\check{a}}_y \mathcal{N}_+^{1/2} \xi \Vert \right] \\&\quad \le C N^{1-\alpha } \Vert (\mathcal{N}_+ + 1)^{1/2} \xi \Vert ^2 + CN^{1/2-\alpha } \Vert (\mathcal{N}_+ + 1)^{1/2} \xi \Vert \Vert \mathcal{V}_N^{1/2} \xi \Vert \end{aligned} \end{aligned}$$

Let us consider the last term in (153). Switching to position space and using (111) in Lemma 4 and again (25), we arrive at

$$\begin{aligned} \begin{aligned}&|\langle \xi , \mathcal{E}_{105}^{(4)} \xi \rangle | \\&\le C N\int _{\varLambda ^2} dx dy \, e^{2N} V(e^N(x-y)) \Vert (\mathcal{N}_+ + 1)^{1/2} \xi \Vert \int _0^1 ds \Vert (\mathcal{N}_+ + 1)^{-1/2} {\check{d}}^{(s)}_x {\check{d}}^{(s)}_y \xi \Vert \\&\le CN \Vert \eta \Vert \Vert (\mathcal{N}_+ + 1)^{1/2} \xi \Vert \int _{\varLambda ^2} dx dy \, e^{2N}V(e^N(x-y)) \\&\quad \times \left[ \Vert (\mathcal{N}_+ + 1)^{1/2} \xi \Vert + \Vert \eta \Vert \Vert {\check{a}}_x \xi \Vert + \Vert \eta \Vert \Vert {\check{a}}_y \xi \Vert + N^{-1/2}\Vert \eta \Vert \Vert {\check{a}}_x {\check{a}}_y \xi \Vert \right] \\&\le C N^{1-\alpha } \Vert (\mathcal{N}_++ 1)^{1/2} \xi \Vert ^2 + CN^{1/2-2\alpha }\Vert (\mathcal{N}_+ + 1)^{1/2} \xi \Vert \Vert \mathcal{V}_N^{1/2} \xi \Vert \,. \end{aligned} \end{aligned}$$

Summarizing, we have shown that (152) can be bounded by

155 $$\begin{aligned} \begin{aligned} |\langle \xi , \mathcal{E}_{10}^{(4)}\xi \rangle | \le&C N^{1/2-\alpha } \Vert \mathcal{V}_N^{1/2}\xi \Vert \Vert (\mathcal{N}_+ +1)^{1/2} \xi \Vert + C N^{1-\alpha } \Vert (\mathcal{N}_+ +1)^{1/2} \xi \Vert ^2 \end{aligned}\end{aligned}$$

for any $\alpha > 1$, $\xi \in \mathcal{F}^{\le N}_+$. Next, we come back to the terms $\text {W}_{11}, \text {W}_{12}, \text {W}_{13}$ introduced in (151). Using (154) and $|\gamma _q^{(s)} -1| \le C \eta _q^2$, we can write

156 $$\begin{aligned} \text {W}_{11} = \frac{1}{2} \sum _{q \in \varLambda _+^* , r \in \varLambda ^* : r \not = -q} \widehat{V} (r/e^N) \eta _{q+r} (b_q b_{-q} + \text{ h.c.}) + \mathcal{E}_{11}^{(4)}\,, \end{aligned}$$

where $\mathcal{E}_{11}^{(4)}$ is such that

$$\begin{aligned} \begin{aligned} | \langle \xi , \mathcal{E}_{11}^{(4)} \xi \rangle |&\le C N^{1-2\alpha } \Vert (\mathcal{N}_+ + 1) \xi \Vert ^2\,. \end{aligned} \end{aligned}$$

Next, we can decompose the second term in (151) as

157 $$\begin{aligned} \text {W}_{12} = \frac{1}{2} \sum _{q \in \varLambda _+^* , r \in \varLambda ^* : r \not = -q} \widehat{V} (r/e^N) \eta _{q+r} \eta _q \left( 1- \frac{\mathcal{N}_+}{N} \right) + \mathcal{E}_{12}^{(4)} \end{aligned}$$

where $$\pm \mathcal{E}^{(4)}_{12} \le C N^{-\alpha } \mathcal{N}_+ + N^{1-3\alpha }$$.

The third term on the r.h.s. of (151) can be written as

158 $$\begin{aligned} \text {W}_{13} = - \frac{1}{2} \sum _{q \in \varLambda _+^* , r \in \varLambda ^* : r \not = -q} \widehat{V} (r/e^N) \eta _{q+r} \eta _q \left( 1- \frac{\mathcal{N}_+}{N} \right) \frac{\mathcal{N}_+ +1}{N} + \mathcal{E}^{(4)}_{13} \end{aligned}$$

where $$\mathcal{E}^{(4)}_{13} = \mathcal{E}_{131}^{(4)} + \mathcal{E}_{132}^{(4)} + \mathcal{E}_{133}^{(4)} + \mathcal{E}_{134}^{(4)}$$, with

$$\begin{aligned} \begin{aligned} \mathcal{E}^{(4)}_{131} = \;&\frac{1}{2} \sum _{q \in \varLambda _+^* , r \in \varLambda ^* : r \not = -q} \widehat{V} (r/e^N) \eta _{q+r} \int _0^1 ds \, (\gamma _q^{(s)} -1) b_q d_{-q}^{(s)} +\text{ h.c. }\\ \mathcal{E}^{(4)}_{132} = \;&\frac{1}{2} \sum _{q \in \varLambda _+^* , r \in \varLambda ^* : r \not = -q} \widehat{V} (r/e^N) \eta _{q+r} \int _0^1 ds \, b_q \left[ d_{-q}^{(s)} + s \eta _q \frac{\mathcal{N}_+}{N} b_{q}^* \right] + \text{ h.c. }\\ \mathcal{E}^{(4)}_{133} = \;&- \frac{1}{2} \sum _{q \in \varLambda _+^* , r \in \varLambda ^* : r \not = -q} \widehat{V} (r/e^N) \eta _{q+r} \eta _q b^*_q b_{q} \frac{\mathcal{N}_+ +1}{N} \\ \mathcal{E}^{(4)}_{134} = \;&\frac{1}{2N} \sum _{q \in \varLambda _+^* , r \in \varLambda ^* : r \not = -q} \widehat{V} (r/e^N) \eta _{q+r} \eta _q a_q^* a_q \frac{\mathcal{N}_+ +1}{N} \,. \end{aligned} \end{aligned}$$

With (154), we immediately find

$$\begin{aligned} \pm \mathcal{E}_{133}^{(4)} \le C N^{1-\alpha } (\mathcal{N}_+ + 1) , \qquad \pm \mathcal{E}_{134}^{(4)} \le C N^{-\alpha } (\mathcal{N}_+ + 1)\,. \end{aligned}$$

With $|\gamma _q^{(s)} -1| \le C \eta _q^2$, Lemma 4 and, again, (154), we also obtain

$$\begin{aligned} \begin{aligned} |\langle \xi , \mathcal{E}_{131}^{(4)} \xi \rangle |&\le C N^{1-3\alpha } \Vert (\mathcal{N}_+ + 1)^{1/2} \xi \Vert ^2\,. \end{aligned} \end{aligned}$$

Let us now consider $\mathcal{E}_{132}^{(4)}$. In position space, with $\check{\overline{d}}^{(s)}_y = d^{(s)}_y + (\mathcal{N}_+ / N) b^* ({\check{\eta }}_{y})$ and using (110), we obtain

$$\begin{aligned} \begin{aligned} |\langle \xi , \mathcal{E}_{132}^{(4)} \xi \rangle |&= \Big | \frac{1}{2} \int _0^1 ds \int _{\varLambda ^2} dx dy\, e^{2N} V(e^N(x-y)) {\check{\eta }} (x-y) \langle \xi , {\check{b}}_x \check{\overline{d}}^{(s)}_y \xi \rangle \Big | \\&\le C N^{1-\alpha } \int _{\varLambda ^2} dx dy \,e^{2N} V(e^N(x-y)) \Vert (\mathcal{N}_+ + 1)^{1/2} \xi \Vert \\&\quad \times \left[ \Vert (\mathcal{N}_+ + 1)^{1/2} \xi \Vert + \Vert {\check{a}}_y\xi \Vert + \Vert {\check{a}}_x \xi \Vert +N^{-1} \Vert {\check{a}}_x {\check{a}}_y \mathcal{N}_+^{1/2} \xi \Vert \right] \\&\le C N^{1-\alpha }\Vert (\mathcal{N}_+ + 1)^{1/2} \xi \Vert ^2 + C N^{1/2-\alpha } \Vert (\mathcal{N}_+ + 1)^{1/2} \xi \Vert \Vert \mathcal{V}_N^{1/2} \xi \Vert \,. \end{aligned} \end{aligned}$$

It follows that

$$\begin{aligned} | \langle \xi , \mathcal{E}_{13}^{(4)} \rangle | \le C N^{1/2 -\alpha } \Vert \mathcal{V}_N^{1/2} \xi \Vert \Vert (\mathcal{N}_++1)^{1/2}\xi \Vert +CN^{1-\alpha }\Vert (\mathcal{N}_++1)^{1/2}\xi \Vert ^2 . \end{aligned}$$

With (155), (156), (157), (158), we obtain

159 $$\begin{aligned} \begin{aligned} \text {W}_1 = \;&\frac{1}{2} \sum _{q \in \varLambda _+^*, r \in \varLambda ^* : r \not = -q} \widehat{V} (r/e^N) \eta _{q+r} \big (b_q b_{-q} + \text{ h.c. }\big ) \\&+\frac{1}{2} \sum _{q \in \varLambda _+^* , r \in \varLambda ^* : r \not = -q} \widehat{V} (r/e^N) \eta _{q+r} \eta _q \left( 1- \frac{\mathcal{N}_+}{N} \right) \left( 1- \frac{\mathcal{N}_+ + 1}{ N} \right) + \mathcal{E}^{(4)}_1 \end{aligned} \end{aligned}$$

where

$$\begin{aligned} | \langle \xi , \mathcal{E}_{1}^{(4)}\xi \rangle | \le C N^{1/2 -\alpha } \Vert \mathcal{V}_N^{1/2} \xi \Vert \Vert (\mathcal{N}_++1)^{1/2}\xi \Vert +CN^{1-\alpha }\Vert (\mathcal{N}_++1)^{1/2}\xi \Vert ^2\,, \end{aligned}$$

Next, we control the term $\text {W}_2$, from (150). In position space, we find

$$\begin{aligned} \begin{aligned} \text {W}_2 = \int _{\varLambda ^2} dx dy\, e^{2N} V(e^N(x-y)) \int _0^1 ds \big ( e^{-sB} {\check{b}}^*_x {\check{b}}^*_y e^{s B} a^* ({\check{\eta }}_{x}) {\check{a}}_y + \text {h.c.} \big ) \end{aligned} \end{aligned}$$

with ${\check{\eta }}_{x} (z) = {\check{\eta }} (x-z)$. By Cauchy–Schwarz, we have

$$\begin{aligned} \begin{aligned} |\langle \xi , \text {W}_2 \xi \rangle |&\le \int _{\varLambda ^2} dx dy \, e^{2N}V(e^N(x-y)) \int _0^1 ds \\&\quad \times \Vert (\mathcal{N}_+ +1)^{1/2} e^{-sB} {\check{b}}_x {\check{b}}_y e^{s B} \xi \Vert \Vert (\mathcal{N}_+ +1)^{-1/2} a^* ({\check{\eta }}_{x}) {\check{a}}_y \xi \Vert \,. \end{aligned} \end{aligned}$$

With

$$\begin{aligned} \Vert (\mathcal{N}_+ +1)^{-1/2} a^* ({\check{\eta }}_{x}) {\check{a}}_y \xi \Vert \le C \Vert \eta \Vert \Vert {\check{a}}_y \xi \Vert \le C N^{-\alpha } \Vert {\check{a}}_y \xi \Vert \end{aligned}$$

and using Lemma 6, we obtain

160 $$\begin{aligned} \begin{aligned} |\langle \xi , \text {W}_2 \xi \rangle |&\le C N^{-\alpha } \int _{\varLambda ^2} dx dy \, e^{2N} V(e^N(x-y)) \Vert {\check{a}}_y \xi \Vert \\&\quad \times \Big \{ N \Vert (\mathcal{N}_+ +1)^{1/2} \xi \Vert + N \Vert {\check{a}}_x \xi \Vert + N \Vert {\check{a}}_y \xi \Vert + N^{1/2} \Vert {\check{a}}_x {\check{a}}_y \xi \Vert \Big \} \\&\le C N^{1-\alpha } \, \Vert (\mathcal{N}_+ +1)^{1/2} \xi \Vert ^2+CN^{1/2-\alpha }\Vert ( \mathcal{N}_+ + 1 )^{1/2} \xi \Vert \Vert \mathcal{V}_N^{1/2}\xi \Vert \,. \end{aligned} \end{aligned}$$

Also for the term $\text {W}_3$ in (150), we switch to position space. We find

$$\begin{aligned} \begin{aligned} \text {W}_3 =&\; \int _{\varLambda ^2} dx dy \, e^{2N} V(e^N(x-y)) \\&\times \int _0^1 ds\, \int _0^s d \tau \, \big ( e^{-sB} {\check{b}}^*_x {\check{b}}^*_y e^{sB} \, e^{- \tau B} b^*({\check{\eta }}_{x}) b^* ({\check{\eta }}_{y}) e^{\tau B} + \text {h.c.} \big ) \,.\end{aligned} \end{aligned}$$

and thus

$$\begin{aligned} \begin{aligned}&\left| \langle \xi , \text {W}_3 \xi \rangle \right| \le \int _{\varLambda ^2} dx dy \, e^{2N} V(e^N(x-y)) \int _0^1 ds\, \int _0^s d \tau \, \Vert (\mathcal{N}_+ +1)^{1/2} e^{-sB} {\check{b}}_x {\check{b}}_y e^{sB} \xi \Vert \\&\quad \times \Vert (\mathcal{N}_+ +1)^{-1/2} e^{- \tau B} b^*({\check{\eta }}_{x})\, b^* ({\check{\eta }}_{y}) e^{ \tau B} \xi \Vert \,. \end{aligned} \end{aligned}$$

With Lemma 2, we find

$$\begin{aligned} \begin{aligned} \Vert (\mathcal{N}_+ +1)^{-1/2} e^{- \tau B} b^*({\check{\eta }}_{x})\, b^*({\check{\eta }}_{y}) e^{ \tau B} \xi \Vert&\le C \Vert \eta \Vert ^2 \Vert (\mathcal{N}_+ +1)^{1/2} \xi \Vert \,. \end{aligned} \end{aligned}$$

Using Lemma 6, we conclude that

161 $$\begin{aligned} \begin{aligned} |\langle \xi , \text {W}_3 \xi \rangle |&\le C \Vert \eta \Vert ^2\, \int _{\varLambda ^2} dx dy \, e^{2N} V(e^N(x-y)) \Vert (\mathcal{N}_+ +1)^{1/2}\xi \Vert \\&\quad \times \Big \{ N \Vert (\mathcal{N}_+ +1)^{1/2} \xi \Vert + N \Vert {\check{a}}_x \xi \Vert + N \Vert {\check{a}}_y \xi \Vert + N^{1/2} \Vert {\check{a}}_x {\check{a}}_y \xi \Vert \Big \} \\&\le C N^{1-2\alpha } \, \Vert (\mathcal{N}_+ +1)^{1/2} \xi \Vert ^2 +CN^{1/2-2\alpha }\Vert \mathcal{V}_N^{1/2} \xi \Vert \Vert (\mathcal{N}_+ +1)^{1/2} \xi \Vert .\end{aligned} \end{aligned}$$

The term $\text {W}_4$ in (150) can be bounded similarly. In position space, we find

$$\begin{aligned} \begin{aligned} \text {W}_4 = \;&\int dxdy \, e^{2N} V(e^N(x-y)) \\&\times \int _0^1 ds \int _0^s d \tau \, \big ( e^{-sB} {\check{b}}^*_x {\check{b}}^*_y \, e^{sB} \, e^{-\tau B} b(\check{\eta ^2_x}) {\check{b}}_y e^{\tau B} + \text {h.c.} \big ) \end{aligned} \end{aligned}$$

with $\check{\eta ^2}$ the function with Fourier coefficients $\eta ^2_q$, for $q \in \varLambda ^*$, and where $\check{\eta ^2_x}(y) := \check{\eta ^2}(x-y)$. Clearly $\Vert \check{\eta ^2_{x}} \Vert \le C \Vert {\check{\eta }}\Vert ^2 \le C N^{-2\alpha }$. With Cauchy–Schwarz and Lemma 2, we obtain

$$\begin{aligned} |\langle \xi , \text {W}_4 \xi \rangle | \le \; C N^{-2\alpha } \int _0^1\, ds \int _0^sd\tau \int \, dx dy \, e^{2N} V(e^N(x-y)) \Vert (\mathcal{N}_+ + 1)^{1/2} {\check{b}}_y {\check{b}}_x e^{sB} \xi \Vert \Vert {\check{b}}_y e^{\tau B} \xi \Vert . \end{aligned}$$

Applying Lemma 6 and then Lemma 2, we obtain

$$\begin{aligned} \begin{aligned} |\langle \xi , \text {W}_4 \xi \rangle | \le&\; C N^{-2\alpha } \int _0^1 ds \int _0^s d\tau \int dx dy\, e^{2N} V(e^N(x-y)) \Vert {\check{b}}_y e^{\tau B} \xi \Vert \\&\quad \times \left\{ N \Vert (\mathcal{N}_+ +1)^{1/2} \xi \Vert + N \Vert {\check{a}}_x \xi \Vert + N \Vert {\check{a}}_y \xi \Vert + N^{1/2} \Vert {\check{a}}_x {\check{a}}_y \xi \Vert \right\} \\ \le \;&C N^{1-2\alpha } \Vert (\mathcal{N}_+ + 1)^{1/2} \xi \Vert ^2 +CN^{1/2-2\alpha } \Vert \mathcal{V}_N^{1/2} \xi \Vert \Vert (\mathcal{N}_+ + 1)^{1/2} \xi \Vert \,. \end{aligned} \end{aligned}$$

From (159), (160), (161) and the last bound, we conclude that

$$\begin{aligned} \begin{aligned} \mathcal{G}^{(4)}_{N,\alpha } = \;&\mathcal{V}_N + \frac{1}{2} \sum _{q \in \varLambda _+^*, r \in \varLambda ^* : r \not = -q} \widehat{V} (r/e^N) \eta _{q+r} \big (b_q b_{-q} + \text{ h.c. }\big ) \\&+\frac{1}{2} \sum _{q \in \varLambda _+^* , r \in \varLambda ^* : r \not = -q} \widehat{V} (r/e^N) \eta _{q+r} \eta _q \left( 1- \frac{\mathcal{N}_+}{N} \right) \left( 1- \frac{\mathcal{N}_+ + 1}{ N} \right) + \mathcal{E}^{(4)}_{N,\alpha } \end{aligned} \end{aligned}$$

where $$\mathcal{E}_{N,\alpha }^{(4)}$$ satisfies (149). $$\square $$

### Appendix A.6: Proof of Proposition 1

With the results established in Sects. 1–1, we cam now show Proposition 1. Propositions 12, 13, 14, 15, 16, imply that

162 $$\begin{aligned} \begin{aligned} \mathcal{G}_{N,\alpha }&= \frac{\widehat{V} (0)}{2}\, (N +\mathcal{N}_+ -1) \, (N-\mathcal{N}_+)\\&\quad + \sum _{p \in \varLambda ^*_+} \,\eta _p \Big [p^2 \eta _p + N\widehat{V} (p/e^N) + \frac{1}{2}\sum _{\begin{array}{c} r \in \varLambda ^*\\ p+r \ne 0 \end{array}} \widehat{V} (r/e^N) \eta _{p+r}\Big ]\Big (\frac{N-\mathcal{N}_+}{N}\Big ) \Big (\frac{N-\mathcal{N}_+ -1}{N}\Big )\\&\quad +\mathcal{K}+N\sum _{p \in \varLambda ^*_+} \widehat{V} (p/e^N) a^*_pa_p\Big (1-\frac{\mathcal{N}_+}{N}\Big ) \\&\quad + \sum _{p \in \varLambda ^*_+} \Big [\; p^2 \eta _p + \frac{N}{2} \widehat{V} (p/e^N) + \frac{1}{2} \sum _{r \in \varLambda ^*:\; p+r \ne 0}\, \widehat{V} (r/e^N) \eta _{p+r} \; \Big ] \big ( b^*_p b^*_{-p} + b_p b_{-p} \big ) \\&\quad + \sqrt{N} \sum _{p,q \in \varLambda ^*_+ :\, p + q \not = 0} \widehat{V} (p/e^N) \left[ b_{p+q}^* a_{-p}^* a_q + \text{ h.c. }\right] +\mathcal{V}_N + \mathcal{E}_{1} \end{aligned} \end{aligned}$$

where

$$\begin{aligned} | \langle \xi , \mathcal{E}_1 \xi \rangle | \le C N^{1/2 -\alpha } \Vert \mathcal{H}_N^{1/2}\xi \Vert \Vert (\mathcal{N}_++1)^{1/2}\xi \Vert + C N^{1-\alpha } \Vert (\mathcal{N}_++1)^{1/2}\xi \Vert ^2 \end{aligned}$$

for any $\alpha >1$ and $\xi \in \mathcal{F}^{\le N}_+$. With (31), we find

$$\begin{aligned} \begin{aligned}&\sum _{p \in \varLambda ^*_+} \eta _p \Big [p^2 \eta _p + N\widehat{V} (p/e^N) + \frac{1}{2} \sum _{r \in \varLambda ^*:\; p+r \ne 0}\, \widehat{V} (r/e^N) \eta _{p+r}\Big ] \\&\quad = \sum _{p \in \varLambda ^*_+} \eta _p \Big [ \;\frac{N}{2} \widehat{V} (p/e^N) +Ne^{2N} \lambda _\ell {{\widehat{\chi }}}_\ell (p) +e^{2N} \lambda _\ell \sum _{q \in \varLambda ^*} {{\widehat{\chi }}}_\ell (p-q) \eta _q - \frac{1}{2} {\widehat{V}}(p/e^N) \eta _0\;\Big ] \end{aligned} \end{aligned}$$

From Lemma 1 and estimating $\Vert \widehat{\chi }_\ell \Vert = \Vert \chi _\ell \Vert \le C N^{-\alpha }$, $\Vert \eta \Vert \le C N^{-\alpha }$ and $\Vert {\widehat{\chi }}_\ell * \eta \Vert = \Vert \chi _\ell {\check{\eta }} \Vert \le \Vert {\check{\eta }} \Vert \le C N^{-\alpha }$, we have

$$\begin{aligned} \Big | Ne^{2N} \lambda _\ell \sum _{p \in \varLambda ^*_+} \eta _p \widehat{\chi }_\ell (p) \Big | \le CN^{2\alpha }\Vert {\widehat{\chi }}_\ell \Vert \Vert \eta \Vert \le C,\end{aligned}$$

and

$$\begin{aligned} \Big | e^{2N} \lambda _\ell \sum _{\begin{array}{c} p \in \varLambda ^*_+,\, q \in \varLambda ^* \end{array}} {{\widehat{\chi }}}_\ell (p-q) \eta _q\eta _p \Big | \le C N^{2\alpha -1} \Vert \hat{\chi }_\ell *\eta \Vert \Vert \eta \Vert \le CN^{-1}\,. \end{aligned}$$

Moreover, using (154) and the bound (32) we find

$$\begin{aligned} \Big | \frac{1}{2} \sum _{p \in \varLambda ^*_+} {\widehat{V}}(p/e^N) \eta _p \eta _0 \Big | \le C N^{1-2\alpha }\,. \end{aligned}$$

We obtain

$$\begin{aligned} \begin{aligned}&\sum _{p \in \varLambda ^*_+} \eta _p \Big [p^2 \eta _p + N\widehat{V} (p/e^N) + \frac{1}{2} \sum _{\begin{array}{c} r \in \varLambda ^*\\ p+r \in \varLambda ^*_+ \end{array}} \widehat{V} (r/e^N) \eta _{p+r}\Big ]\Big (\frac{N-\mathcal{N}_+}{N}\Big ) \Big (\frac{N-\mathcal{N}_+ -1}{N}\Big ) \\&\qquad = \frac{N}{2} \sum _{p \in \varLambda ^*_+} \widehat{V} (p/e^N) \eta _p \left( \frac{N-\mathcal{N}_+}{N} \right) \left( \frac{N-\mathcal{N}_+ - 1}{N} \right) +\mathcal{E}_2 \end{aligned} \end{aligned}$$

with $\pm \mathcal{E}_2 \le C$ for all $\alpha \ge 1/2$. On the other hand, using (32) we have

$$\begin{aligned} \begin{aligned} \frac{N}{2} \sum _{p \in \varLambda ^*_+} \widehat{V} (p/e^N) \eta _p =\;&\frac{N}{2} \big ( {\widehat{V}}(\cdot / e^N) *\eta \big )(0) - \frac{N}{2} \widehat{V} (0) \eta _0 \\ =\;&\frac{N^2}{2} \Big ( \int dx V(x) f_{\ell }(x) - {\widehat{V}}(0) \Big ) + {\tilde{\mathcal{E}}}_2 \end{aligned}\end{aligned}$$

with $\pm {\tilde{\mathcal{E}}}_2 \le C N^{1-2\alpha }$. With the first bound in (41) we conclude that

163 $$\begin{aligned} \begin{aligned}&\sum _{p \in \varLambda ^*_+} \eta _p \Big [p^2 \eta _p + N\widehat{V} (p/e^N) + \frac{1}{2} \sum _{\begin{array}{c} r \in \varLambda ^*\\ p+r \in \varLambda ^*_+ \end{array}} \widehat{V} (r/e^N) \eta _{p+r}\Big ]\Big (\frac{N-\mathcal{N}_+}{N}\Big ) \Big (\frac{N-\mathcal{N}_+ -1}{N}\Big ) \\&= \frac{1}{2N} \left[ {\widehat{\omega }}_N(0) - N\widehat{V} (0) \right] (N-\mathcal{N}_+ -1) \left( N-\mathcal{N}_+ \right) + \mathcal{E}_3 \end{aligned} \end{aligned}$$

where $\pm \mathcal{E}_3 \le C$, if $\alpha \ge 1/2$. Using (31), we can also handle the fourth line of (162); we find

164 $$\begin{aligned} \begin{aligned}&\sum _{p \in \varLambda ^*_+} \Big [\, p^2 \eta _p + \frac{N}{2} \widehat{V} (p/e^N) + \frac{1}{2} \sum _{r \in \varLambda ^*:\; p+r \in \varLambda ^*_+}\, \widehat{V} (r/e^N) \eta _{p+r} \, \Big ] \big ( b^*_p b^*_{-p} + b_p b_{-p} \big ) \\&= \sum _{p \in \varLambda ^*_+} \Big [ Ne^{2N} \lambda _\ell {\widehat{\chi }}_\ell (p) +e^{2N} \lambda _\ell \sum _{q \in \varLambda ^*} {\widehat{\chi }}_\ell (p-q) \eta _q - \frac{1}{2} \widehat{V} (p/e^N) \eta _{0} \Big ] \big ( b^*_p b^*_{-p} + b_p b_{-p} \big )\,. \end{aligned} \end{aligned}$$

The last two terms on the right hand side of (164) are error terms. With (32) and (154) we have

$$\begin{aligned} \begin{aligned} \Big | \sum _{p \in \varLambda ^*_+}&\widehat{V} (p/e^N) \eta _{0} \big ( b^*_p b^*_{-p} + b_p b_{-p} \big ) \Big | \\&\le C N^{-2\alpha } \bigg [ \sum _{p \in \varLambda ^*_+} \frac{|\widehat{V} (p/e^N)|^2}{p^2} \bigg ]^{1/2} \bigg [ \sum _{p \in \varLambda ^*_+} p^2 \Vert a_p \xi \Vert ^2 \bigg ]^{1/2} \Vert (\mathcal{N}_++1)^{1/2} \xi \Vert \\&\le C N^{1/2 - 2\alpha } \Vert \mathcal{K}^{1/2}\xi \Vert \Vert (\mathcal{N}_++1)^{1/2} \xi \Vert \,. \end{aligned}\end{aligned}$$

The second term on the right hand side of (164) can be bounded in position space:

$$\begin{aligned}\begin{aligned}&\Big | \langle \xi ,\; e^{2N} \lambda _\ell \sum _{p\in \varLambda ^*_+} ({\widehat{\chi }}_\ell * \eta )(p) (b_p^* b_{-p}^* + b_p b_{-p}) \xi \rangle \Big | \\&\quad \le CN^{2\alpha -1} \Vert (\mathcal{N}_+ + 1)^{1/2} \xi \Vert \int _{\varLambda ^2} dxdy \,\chi _\ell (x-y) |{\check{\eta }}(x-y)|\Vert (\mathcal{N}_+ + 1)^{-1/2}{\check{b}}_x{\check{b}}_y\xi \Vert \\&\quad \le CN^{\alpha -1}\Vert (\mathcal{N}_+ + 1)^{1/2} \xi \Vert \left[ \int _{\varLambda ^2} dxdy \, \chi _\ell (x-y) \Vert (\mathcal{N}_+ + 1)^{-1/2}{\check{a}}_x{\check{a}}_y\xi \Vert ^2\right] ^{1/2}. \end{aligned}\end{aligned}$$

The term in parenthesis can be bounded similarly as in (80). Namely,

$$\begin{aligned} \int _{\varLambda ^2} dxdy \, \chi _\ell (x-y) \Vert (\mathcal{N}_+ + 1)^{-1/2}{\check{a}}_x{\check{a}}_y\xi \Vert ^2 \le Cq N^{-2\alpha /q'}\Vert \mathcal{K}^{1/2}\xi \Vert ^2 \end{aligned}$$

for any $q >2$ and $1< q' < 2$ with $1/q +1/q' =1$. Choosing $q =\log N$, we get

$$\begin{aligned} \begin{aligned} \Big | \langle \xi ,e^{2N} \lambda _\ell&\sum _{p\in \varLambda ^*_+} ({\widehat{\chi }}_\ell * \eta )(p) (b_p^* b_{-p}^* + b_p b_{-p}) \xi \rangle \Big | \\&\le C N^{-1} (\log N)^{1/2} \Vert (\mathcal{N}_+ + 1)^{1/2} \xi \Vert \Vert \mathcal{K}^{1/2}\xi \Vert \,, \end{aligned}\end{aligned}$$

and, from (164), we conclude that

165 $$\begin{aligned} \begin{aligned} \sum _{p \in \varLambda ^*_+}&\Big [p^2 \eta _p + \frac{N}{2} \widehat{V} (p/e^N) + \frac{1}{2} \sum _{\begin{array}{c} r \in \varLambda ^* : \\ p+r \in \varLambda ^*_+ \end{array}} \widehat{V} (r/e^N) \eta _{p+r} \Big ] \big ( b^*_p b^*_{-p} + b_p b_{-p} \big ) \\&= \sum _{p \in \varLambda ^*_+} Ne^{2N} \lambda _\ell {\widehat{\chi }}_\ell (p)\big ( b^*_p b^*_{-p} + b_p b_{-p} \big ) + \mathcal{E}_4\,, \end{aligned}\end{aligned}$$

with

$$\begin{aligned} | \langle \xi , \mathcal{E}_4 \xi \rangle | \le C N^{-1} (\log N)^{1/2} \Vert (\mathcal{N}_++1)^{1/2}\xi \Vert \Vert \mathcal{K}^{1/2}\xi \Vert .\end{aligned}$$

if $\alpha > 1$. Combining (162) with (163) and (165), and using the definition (39) we conclude that

166 $$\begin{aligned} \begin{aligned} \mathcal{G}_{N,\alpha } = \;&\frac{1}{2} \,{\widehat{\omega }}_N(0) (N-1)\Big (1-\frac{\mathcal{N}_+}{N}\Big ) + \Big [N \widehat{V} (0)- \frac{1}{2} \,{\widehat{\omega }}_N(0) \Big ]\, \mathcal{N}_+\Big (1-\frac{\mathcal{N}_+}{N}\Big )\\&+ N \sum _{p \in \varLambda ^*_+} \widehat{V} (p/e^N) a^*_pa_p \left( 1- \frac{\mathcal{N}_+}{N} \right) + \frac{1}{2} \sum _{ p\in \varLambda _+^*}{\widehat{\omega }}_N(p)(b_pb_{-p}+\text{ h.c.})\\&+ \sqrt{N} \sum _{p,q \in \varLambda ^*_+ : p + q \not = 0} \widehat{V} (p/e^N) \left[ b_{p+q}^* a_{-p}^* a_q + \text{ h.c. }\right] \\&+\mathcal{K}+\mathcal{V}_N + \mathcal{E}_5 \,, \end{aligned} \end{aligned}$$

with

$$\begin{aligned}\begin{aligned} | \langle \xi , \mathcal{E}_5 \xi \rangle | \le \;&C N^{1/2 -\alpha } \Vert \mathcal{H}_N^{1/2}\xi \Vert \Vert (\mathcal{N}_++1)^{1/2}\xi \Vert + C N^{1-\alpha } \Vert (\mathcal{N}_++1)^{1/2}\xi \Vert ^2 \\&+ C N^{-1} (\log N)^{1/2} \Vert \mathcal{K}^{1/2}\xi \Vert \Vert (\mathcal{N}_++1)^{1/2}\xi \Vert + C \Vert \xi \Vert ^2 \,, \end{aligned} \end{aligned}$$

for any $\alpha > 1$. Observing that $|\widehat{V} (p/e^N) - \widehat{V} (0)| \le C |p| e^{-N}$ in the second line on the r.h.s. of (166), we arrive at $\mathcal{G}_{N,\alpha } = \mathcal{G}^\text {eff}_{N,\alpha } + \mathcal{E}_{\mathcal{G}}$, with $\mathcal{G}^\text {eff}_{N,\alpha }$ defined as in (42) and with $\mathcal{E}_{\mathcal{G}}$ that satisfies (43).

## Appendix B: Properties of the Scattering Function

Let *V* be a potential with finite range $R_0>0$ and scattering length ${{\mathfrak {a}}}$. For a fixed $R>R_0$, we study properties of the ground state $f_R$ of the Neumann problem

167 $$\begin{aligned} \Big ( -\varDelta + \frac{1}{2} V(x) \Big ) f_{R}(x) = \lambda _{R}\, f_{R}(x) \end{aligned}$$

on the ball $|x|\le R$, normalized so that $f_{R}(x)=1$ for $|x|=R$. Lemma 1, parts (i)–(iv), follows by setting $R=e^N \ell$ in the following lemma.

### Lemma 7

Let $V\in L^3({\mathbb {R}}^2)$ be non-negative, compactly supported and spherically symmetric, and denote its scattering length by ${{\mathfrak {a}}}$. Fix $R>0$ sufficiently large and denote by $f_{R}$ the Neumann ground state of (167). Set $w_{R}=1 - f_{R}$. Then we have

$$\begin{aligned} 0\le f_R(x)\le 1 \end{aligned}$$

Moreover, for *R* large enough there is a constant $C>0$ independent of *R* such that

168 $$\begin{aligned} \left| \,\lambda _{R} - \frac{2}{R^2 \log (R/{{\mathfrak {a}}})} \left( 1 + \frac{3}{4}\frac{1}{\log (R/{{\mathfrak {a}}})} \right) \,\right| \le \frac{C}{R^2} \frac{1}{\log ^3(R/{{\mathfrak {a}}})} \,. \end{aligned}$$

and

169 $$\begin{aligned} \left| \int \text {d}x\, V(x) f_{R}(x) - \frac{4\pi }{\log (R/{{\mathfrak {a}}})} \right| \le \frac{C}{\log ^2 (R/{{\mathfrak {a}}})} \,. \end{aligned}$$

Finally, there exists a constant $C>0$ such that

170 $$\begin{aligned} \begin{aligned} |w_{R}(x)|&\le \chi (|x|\le R_0)+ C\, \frac{\log (|x|/R) }{\log ({{\mathfrak {a}}}/R)} \,\chi (R_0 \le |x| \le R ) \\ |\nabla w_{R}(x)|&\le \frac{C}{\log (R /{{\mathfrak {a}}})} \frac{ \chi (|x|\le R)}{|x| + 1} \end{aligned} \end{aligned}$$

for *R* large enough.

To show Lemma 7 we adapt to the two dimensional case the strategy used in [8, Lemma A.1] for the three dimensional problem. We will use some well known properties of the zero energy scattering equation in two dimensions, summarized in the following lemma.

### Lemma 8

Let $V \in L^3 ({\mathbb {R}}^2)$ non-negative, with $\text {supp } V \subset B_{R_0} (0)$ for an $R_0 > 0$. Let ${{\mathfrak {a}}}\le R_0$ denote the scattering length of *V*. For $R > R_0$, let $\phi _R : {\mathbb {R}}^2 \rightarrow {\mathbb {R}}$ be the radial solution of the zero energy scattering equation

171 $$\begin{aligned} \left[ -\varDelta + \frac{1}{2} V \right] \phi _R= 0 \end{aligned}$$

normalized such that $\phi _R (x) = 1$ for $|x| = R$. Then

172 $$\begin{aligned} \phi _R(x) = \frac{\log (|x| / {{\mathfrak {a}}})}{\log (R/{{\mathfrak {a}}})} \end{aligned}$$

for all $|x| > R_0$. Moreover, $|x| \rightarrow \phi _R (x)$ is monotonically increasing and there exists a constant $C > 0$ (depending only on *V*) such that

173 $$\begin{aligned} \phi _R (x) \ge \phi _R (0) \ge \frac{C}{\log (R/ {{\mathfrak {a}}})} \end{aligned}$$

for all $x \in {\mathbb {R}}^2$. Furthermore, there exists a constant $C > 0$ such that

174 $$\begin{aligned} |\nabla \phi _R(x)| \le \frac{C}{|\log (R/{{\mathfrak {a}}})|} \frac{1}{|x|+1} \end{aligned}$$

for all $$x \in {\mathbb {R}}^2$$.

### Proof

The existence of the solution of (171), the expression (172), the fact that $\phi _R (x) \ge 0$ and the monotonicity are standard (see, for example, Theorem C.1 and Lemma C.2 in [17]). The bound (173) for $\phi _R (0)$ follows from (172), comparing $\phi _R (0)$ with $\phi _R (x)$ at $|x| = R_0$, with Harnack’s inequality (see [24, Theorem C.1.3]). Finally, (174) follows by rewriting (171) in integral form

$$\begin{aligned} \phi _R (x) = 1 - \frac{1}{4\pi } \int _{{\mathbb {R}}^2} \log \Big ( R/|x-y| \Big ) V(y) \phi _R (y) \text {d}y \,. \end{aligned}$$

For $|x| \le R_0$, this leads (using that $\phi _R (y) \le \log (R_0/{{\mathfrak {a}}}) / \log (R/{{\mathfrak {a}}})$ for all $|y| \le R_0$ and the local integrability of $|.|^{-3/2}$) to

$$\begin{aligned} |\nabla \phi _R (x) | \le C \int \frac{V(y) \phi _R(y)}{|x-y|} dy \le \frac{C \Vert V \Vert _3}{\log (R/{{\mathfrak {a}}})} \end{aligned}$$

Combining with the bound for $|x| > R_0$ obtained differentiating (172), we obtain the desired estimate. $\square$

### Proof of Lemma 7

By standard arguments (see for example [17, proof of theorem C1]), $f_R(x)$ is spherically symmetric and non-negative. We now start by proving an upper bound for $\lambda _R$, consistent with (168). To this end, we calculate the energy of a suitable trial function. For $k \in {\mathbb {R}}$ we define

$$\begin{aligned} \psi _k (x)= J_0(k |x|) - \frac{J_0(k {{\mathfrak {a}}})}{Y_0(k{{\mathfrak {a}}})} Y_0(k|x|)\,. \end{aligned}$$

with $J_0$ and $Y_0$ the zero Bessel functions of first and second type, respectively. Note that

$$\begin{aligned} - \varDelta \psi _k (x) = k^2 \psi _k (x)\,. \end{aligned}$$

and $\psi _k (x)=0$ if $|x| =a$. We define $k= k(R)$ to be the smallest positive real number satisfying $\partial _r \psi _R (x)=0$ for $|x| = R$. One can check that

175 $$\begin{aligned} \left| \,k^2 - \frac{2}{R^2 \log (R/{{\mathfrak {a}}})} \left( 1 + \frac{3}{4}\frac{1}{\log (R/{{\mathfrak {a}}})} \right) \,\right| \le \frac{C}{R^2} \frac{1}{\log ^3 (R/{{\mathfrak {a}}})} \end{aligned}$$

in the limit $R\rightarrow \infty$. To prove (175), we observe that

176 $$\begin{aligned} \partial _r \psi _k (x)\Big |_{|x| = R} = - k J_1 (kR) + k \frac{J_0(k {{\mathfrak {a}}})}{Y_0(k {{\mathfrak {a}}})} Y_1(kR) \,, \end{aligned}$$

and we expand for $kR, k{{\mathfrak {a}}}\ll 1$ using (with $\gamma$ the Euler constant)

177 $$\begin{aligned} \begin{aligned}&\left| J_0(r) -1 +\frac{r^2}{4} \right| \le C r^4 \,, \left| J_1(r) - \frac{r}{2} \Big (1-\frac{r^2}{8}\Big ) \right| \le C r^5\,, \\&\left| Y_0(r) - \frac{2}{\pi }\log (r e^\gamma /2) \right| \le C r^2 \log (r) \,, \\&\left| Y_1(r) + \frac{2}{\pi }\frac{1}{r} \left( 1-\frac{r^2}{2} \Big (1-\frac{r^2}{8}\Big ) \log (re^\gamma /2) +\frac{r^2}{4}\right) \right| \le C r^3. \end{aligned}\end{aligned}$$

With (177) one finds that (176)

178 $$\begin{aligned} \begin{aligned} \partial _r \psi _R&(x)\Big |_{|x| = R} \\ = \;&-\frac{1}{2 kR \log (k{{\mathfrak {a}}}e^\gamma / 2)} \\&\cdot \left\{ \frac{(kR)^4}{8} \log (R/{{\mathfrak {a}}}) - (kR)^2 \left[ \log (R/{{\mathfrak {a}}}) - \frac{1}{2} \right] + 2 + \mathcal{O}( (kR)^4 + (k{{\mathfrak {a}}})^2) \right\} \end{aligned} \end{aligned}$$

The smallest solution of

$$\begin{aligned} \frac{(kR)^4}{8} \log (R/{{\mathfrak {a}}}) - (kR)^2 \left[ \log (R/{{\mathfrak {a}}}) - \frac{1}{2} \right] + 2 = 0 \end{aligned}$$

is such that

179 $$\begin{aligned} (kR)^2 = \frac{2}{\log (R/{{\mathfrak {a}}})} \left[ 1 + \frac{3}{4 \log (R/{{\mathfrak {a}}})} \right] + \mathcal{O}(\log ^{-3} (R/{{\mathfrak {a}}})) \end{aligned}$$

in the limit of large *R*. Inserting in (178), we find that the r.h.s. changes sign around the value of *k* defined in (179). By the intermediate value theorem, we conclude that there is a $k = k (R) > 0$ satisfying (175), such that $\partial _r \psi _{k(R)} (x) = 0$ if $|x| = R$.

Now, let $\phi _R (x)$ be the solution of the zero energy scattering equation (171), with $\phi _R(x)=1$ for $|x| = R$. We set

180 $$\begin{aligned} \varPsi _R (x) := \psi _{k} (m_R(x)) = J_0 (k m_R (x)) - \frac{J_0(k{{\mathfrak {a}}})}{Y_0(k{{\mathfrak {a}}})} Y_0(k m_R(x))\,, \end{aligned}$$

with $k = k(R)$ satisfying (175) and

$$\begin{aligned} m_R(x):= {{\mathfrak {a}}}\exp \big ( \log (R/{{\mathfrak {a}}}) \phi _{R}(x)\big )\,. \end{aligned}$$

With this choice we have $m_R(x)=|x|$ outside the range of the potential; hence $\varPsi _R (x)=\psi _{k} (x)$ for $R_0 \le |x| \le R$. In particular, $\varPsi _R$ satisfies Neumann boundary conditions at $|x|=R$.

From (172), (173) and the monotonicity of $\phi _R$, we get

181 $$\begin{aligned} C {{\mathfrak {a}}}\le m_R(x) \le R_0 \qquad \text {for all} \quad 0\le |x| \le R_0 \, \end{aligned}$$

and for a constant $C > 1$, independent of *R*. From (174) we also get

182 $$\begin{aligned} |\nabla m_R(x)| \le C \qquad \text {for all } \quad 0\le |x| \le R \,. \end{aligned}$$

With the notation ${{\mathfrak {h}}}= -\varDelta + \frac{1}{2} V$, we now evaluate ${\bigl \langle {\varPsi _R, {{\mathfrak {h}}} \varPsi _R}\bigr \rangle }$. To this end we note that

183 $$\begin{aligned} {\bigl \langle {\varPsi _R, {\mathfrak {h}} \varPsi _R}\bigr \rangle } = \int _{|x| < R_0} \overline{\varPsi _R(x)} ( {\mathfrak {h}} \varPsi _R(x)) dx + k^2 \int _{|x| \ge R_0} |\varPsi _R(x)|^2 \, dx \,. \end{aligned}$$

Let us consider the region $|x| < R_0$. From (180) and (177) we find, first of all,

184 $$\begin{aligned} \left| \varPsi _R(x) + \frac{\log (m_R(x)/{{\mathfrak {a}}})}{\log (k {{\mathfrak {a}}}e^\gamma /2)}\right| \le C ( k m_R (x))^2\,, \end{aligned}$$

Next, we compute $-\varDelta \varPsi _R (x)$. With

$$\begin{aligned} J'_0(r)&=- J_1(r)&J'_1(r) =\frac{1}{2} \big ( J_0(r)- J_2(r)\big ) \\ Y'_0(r)&= - Y_1(r)&Y'_1(r) =\frac{1}{2} \big ( Y_0(r)- Y_2(r)\big ) \,. \end{aligned}$$

we obtain (here, we use the notation $m'_R$ and $m''_R$ for the radial derivatives of the radial function $m_R$)

$$\begin{aligned} \begin{aligned} - \varDelta \varPsi _R (x) =\;&- \partial ^2_r \varPsi _R (x) - \frac{1}{|x|} \partial _r \varPsi _R (x) \\ =\;&- k m_R'' (x) \big [ -J_1(k m_R (x) ) + \frac{J_0(k{{\mathfrak {a}}})}{Y_0(k {{\mathfrak {a}}})} Y_1(km_R(x)) \big ] \\&- \frac{1}{2} k^2 \big (m_R'(x)\big )^2 \big [ J_2(k m_R(x) ) - \frac{J_0(k {{\mathfrak {a}}})}{Y_0(k {{\mathfrak {a}}})} Y_2(km_R(x)) \big ] \\&- \frac{1}{2} k^2 \big (m_R'(x)\big )^2 \big [ -J_0(k m_R(x) ) + \frac{J_0(k{{\mathfrak {a}}})}{Y_0(k{{\mathfrak {a}}})} Y_0(km_R (x)) \big ] \\&- \frac{k m_R'(x)}{|x|} \big [ -J_1(k m_R(x) ) + \frac{J_0(k{{\mathfrak {a}}})}{Y_0(k{{\mathfrak {a}}})} Y_1(km_R(x)) \big ] \,. \end{aligned}\end{aligned}$$

We note that, using the scattering equation (171),

185 $$\begin{aligned} m_{R}'' - \frac{(m_R')^2}{m_R} + \frac{1}{|x|} m_R' = \frac{1}{2} V m_R \, \phi _R \log (R/{{\mathfrak {a}}})= \frac{1}{2} V m_R \log (m_R/{{\mathfrak {a}}})\,. \end{aligned}$$

Now we write

186 $$\begin{aligned} \begin{aligned}&- \varDelta \varPsi _R (x) \\&= \Big [ -k \Big ( m''_R (x) + \frac{m'_R(x)}{|x|} \Big )Y_1(km_R (x)) + \frac{k^2}{2} (m'_R(x))^2 Y_2(km_R(x)) \Big ] \frac{J_0(k{{\mathfrak {a}}})}{Y_0(k {{\mathfrak {a}}})} + g_R(x) \end{aligned}\end{aligned}$$

where $$g_R(x)= \sum _{i=1}^3 g_R^{(i)}(x)$$ with

$$\begin{aligned} \begin{aligned} g_R^{(1)}(x)&= k \,\Big ( m''_R(x) +\frac{m'_R(x)}{|x|} \Big ) J_1(k m_R(x)) \\ g_R^{(2)}(x)&= - \frac{1}{2} k^2 (m'_R(x))^2 J_2(k m_R(x)) \\ g_R^{(3)}(x)&= -\frac{1}{2} k^2 (m'_R(x))^2 \Big ( -J_0(k m_R(x) +\frac{J_0(k{{\mathfrak {a}}})}{Y_0(k{{\mathfrak {a}}})} Y_0(km_R (x)) \Big )= \frac{k^2}{2} (m'_R(x))^2 \varPsi _R (x) \,. \end{aligned}\end{aligned}$$

With (185), (177) and (181), (182), we find

$$\begin{aligned} |g_R^{(1)}(x) | \le C k^2 \Big ( (m'_R(x))^2 + \frac{1}{2} V(x) m_R^2 (x) \log (m_R(x)/{{\mathfrak {a}}})\Big ) \le C k^2 (1+V(x)) \,. \end{aligned}$$

Next, with $| J_2(r) - r^2/8 | \le C r^4$ we get

$$\begin{aligned} |g_R^{(2)}(x) |\le C k^4 (m'_R(x))^2 (m_R(x))^2 \le C k^4\,. \end{aligned}$$

With (184), we can also bound

$$\begin{aligned} |g_R^{(3)}(x)| \le C k^2 (m'_R(x))^2 \frac{\log (m_R(x)/{{\mathfrak {a}}})}{\log (k {{\mathfrak {a}}})} \le C k^2 \log ^{-1}(k {{\mathfrak {a}}})\,. \end{aligned}$$

We conclude that $|g_R(r)| \le C (1+V(x)) k^2$ for all $r \le R_0$ and *R* large enough. Finally, using Eq. (185), the expansion for $Y_1(r)$ in Eq. (177), and the bound

$$\begin{aligned} \Big |Y_2(r) + \frac{4}{\pi }\frac{1}{r^2} \Big | \le C \,, \end{aligned}$$

we can rewrite the first term on the r.h.s. of (186) as

187 $$\begin{aligned} \begin{aligned}&\Big [ -k \Big ( m''_R(x) + \frac{m'_R(x)}{|x|} \Big )Y_1(km_R(x)) + \frac{k^2}{2} (m'_R(x))^2 Y_2(km_R(x)) \Big ] \frac{J_0(k{{\mathfrak {a}}})}{Y_0(k {{\mathfrak {a}}})} \\&\qquad = \frac{1}{\pi }V (x) \log (m_R(x)/{{\mathfrak {a}}}) \frac{J_0(k{{\mathfrak {a}}})}{Y_0(k {{\mathfrak {a}}})} + h_R(x) \end{aligned}\end{aligned}$$

with $|h_R(x)|\le C (1+V(x)) k^2$ for all $r \le R_0$, *R* large enough. With the identities (186) and (187) we obtain

$$\begin{aligned} \bigg | - \varDelta \varPsi _R (x)- \frac{1}{\pi }\frac{J_0(k{{\mathfrak {a}}})}{Y_0(k{{\mathfrak {a}}})} V (x) \log (m_R(x)/{{\mathfrak {a}}}) \bigg | \le C (1+V(x)) k^2\,, \end{aligned}$$

for all $|x| \le R_0$ and for *R* sufficiently large. With (184), we conclude that, for $0 \le |x| \le R_0$,

188 $$\begin{aligned} \Big |(- \varDelta + \frac{1}{2} V )\varPsi _R (x) \Big | \le C (1+V(x)) k^2 \,. \end{aligned}$$

With (183), (188) and the upper bound

189 $$\begin{aligned} |\varPsi _R(r)| \le \frac{C}{|\log (k {{\mathfrak {a}}})|} \end{aligned}$$

for all $|x| \le R_0$ (which follows from (184) and (181)), we get

$$\begin{aligned} {\bigl \langle {\varPsi _R, {{\mathfrak {h}}} \varPsi _R}\bigr \rangle } \le k^2 {\bigl \langle {\varPsi _R, \varPsi _R}\bigr \rangle }+ \frac{C k^2}{|\log (k{{\mathfrak {a}}}) |} \int _{|x| \le R_0} (1+ V(x)) \, \text {d}x \,. \end{aligned}$$

On the other hand, Eq.(184), together with $m_R(x)=|x|$ for $|x| \ge R_0$, implies the lower bound

$$\begin{aligned} {\bigl \langle {\varPsi _R,\varPsi _R}\bigr \rangle } \ge \int _{R_0 \le |x| \le R} |\varPsi _R(x)|^2 \text {d}x \ge \frac{C}{|\log (k{{\mathfrak {a}}})|^2} \int _{R_0 \le |x| \le R} \log ^2(|x|/{{\mathfrak {a}}}) \text {d}x \ge CR^2 \,. \end{aligned}$$

Hence, with (175), we conclude that

190 $$\begin{aligned} \begin{aligned} \lambda _{R} \le \frac{{\bigl \langle {\varPsi _R, {\mathfrak {h}} \varPsi _R}\bigr \rangle }}{{\bigl \langle {\varPsi _R,\varPsi _R}\bigr \rangle }}&\le k^2 \, \left( 1 + \frac{C\, |\log (k{{\mathfrak {a}}})|}{R^2} \right) \\&\le \frac{2}{R^2 \log (R/{{\mathfrak {a}}})} \left( 1 + \frac{3}{4}\frac{1}{\log (R/{{\mathfrak {a}}})}+ \frac{C}{\log ^2(R/{{\mathfrak {a}}})} \right) \, \end{aligned}\end{aligned}$$

in agreement with (168).

To prove the lower bound for $\lambda _R$ it is convenient to show some upper and lower bounds for $f_R$. We start by considering $f_R$ outside the range of the potential. We denote $\varepsilon _R= \sqrt{\lambda _R}\, R$. Keeping into account the boundary conditions at $|x| = R$, we find, for $R_0 \le |x| \le R$,

$$\begin{aligned} f_{R} (x) =A_R \, J_0( \varepsilon _R\,|x| /R) + B_R \, Y_0( \varepsilon _R\, |x| /R)\,, \end{aligned}$$

with

$$\begin{aligned} A_R =\left( J_0(\varepsilon _R) - J_1(\varepsilon _R)\, \frac{Y_0(\varepsilon _R)}{Y_1(\varepsilon _R)} \right) ^{-1} \,, \end{aligned}$$

and

$$\begin{aligned} B_R =\left( Y_0(\varepsilon _R) - \frac{J_0(\varepsilon _R)}{J_1(\varepsilon _R)} Y_1(\varepsilon _R) \right) ^{-1} \,. \end{aligned}$$

From (190), we have $|\varepsilon _R|\le C\, |\log (R/{{\mathfrak {a}}})|^{-1/2}$. Thus, we can expand $f_R$ for large *R*, using (177) and, for $Y_0$, the improved bound

$$\begin{aligned} \left| Y_0 (r) -\frac{2}{\pi }\log (re^\gamma /2) \left( 1-\frac{1}{4} r^2\right) \right| \le C \, r^2\, , \end{aligned}$$

we find

191 $$\begin{aligned} \begin{aligned} \Big | A_R - 1 + \frac{\varepsilon _R^2}{4} \Big ( 2 \log (\varepsilon _R e^\gamma /2) - 1 \Big ) \Big |&\le C \varepsilon _R^4 (\log \varepsilon _R)^2\,, \\ \Big | B_R - \frac{\pi }{4} \varepsilon _R^2 \left( 1 -\frac{\varepsilon _R^2}{8}\right) \Big |&\le C \varepsilon _R^6 \, . \end{aligned}\end{aligned}$$

which leads to

192 $$\begin{aligned} \begin{aligned}&\left| f_R (x) -1 +\frac{\varepsilon _R^2}{4} \left( 2\log ( R/|x|) - 1 +\frac{x^2}{R^2} \right) - \frac{\varepsilon _R^4}{16} \log (R/|x|) \left( 1 + \frac{ 2x^2}{R^2} \right) \right| \\&\quad \le C \varepsilon _R^4 (\log \varepsilon _R)^2 \,. \end{aligned} \end{aligned}$$

We can also compute the radial derivative

$$\begin{aligned} \partial _r f_R (x)= - \frac{\varepsilon _R}{R} \Big ( A_R \, J_1( \varepsilon _R\,r/R) + B_R \, Y_1( \varepsilon _R\, r/R)\Big )\,. \end{aligned}$$

With the expansions (177) and (191) we conclude that for all $R_0 \le |x| < R$ we have

193 $$\begin{aligned} \left| \partial _r f_R (x)- \frac{\varepsilon _R^2}{2|x|} \left( 1 - \frac{x^2}{R^2} + \frac{\varepsilon _R^2 x^2}{2R^2} \log (R/|x|) \right) \right| \le C \varepsilon _R^4 \log \varepsilon _R\,. \end{aligned}$$

The bound (193) shows that $\partial _r f_R (x)$ is positive, for, say, $R_0< |x| < R/2$. Since $\partial _r f_R (x)$ must have its first zero at $|x| = R$, we conclude that $f_R$ is increasing in |*x*|, on $R_0 \le |x| \le R$. From the normalization $f_R (x) = 1$, for $|x| = R$, we conclude therefore that $f_R (x) \le 1$, for all $R_0 \le |x| \le R$.

From (192) and (190) we obtain, on the other hand, the lower bound

194 $$\begin{aligned} \begin{aligned} f_R (x)&\ge 1 -\frac{\varepsilon _R^2}{2} \log ( R/ |x|) -C \varepsilon _R^4 (\log \varepsilon _R)^2 \\&\ge 1 - \frac{\log ( R/|x|)}{\log (R/{{\mathfrak {a}}})}\left( 1 + \frac{3}{4} \frac{1}{\log (R/{{\mathfrak {a}}})} + \frac{C}{\log ^2(R/{{\mathfrak {a}}})}\right) - C \,\frac{(\log \log (R/{{\mathfrak {a}}}))^2}{\log ^2(R/{{\mathfrak {a}}})}\\&\ge \frac{\log (|x| /{{\mathfrak {a}}})}{\log (R/{{\mathfrak {a}}})} - \frac{3}{4} \frac{\log (R/|x|)}{\log ^2(R/{{\mathfrak {a}}})} - C \frac{\log (R/|x|)}{\log ^3 (R/a)} - C\, \frac{ (\log \log (R/{{\mathfrak {a}}}))^2}{\log ^2(R/{{\mathfrak {a}}})}\,, \end{aligned}\end{aligned}$$

for *R* sufficiently large. Let $R_*=\max \{R_0, e {{\mathfrak {a}}}\}$. Then Eq. (194) implies in particular that, for *R* large enough,

195 $$\begin{aligned} f_R (x) \ge \frac{C}{\log (R/{{\mathfrak {a}}})}\,. \end{aligned}$$

for all $$R_* < |x| \le R$$.

Finally, we show that $f_R (x) \le 1$ also for $|x| \le R_0$. First of all, we observe that, by elliptic regularity, as stated for example in [12, Theorem 11.7, part iv)], there exists $0<\alpha <1$ and $C > 0$ such that

$$\begin{aligned} \left| f_R (x)- f_R(y) \right| \le C \Vert (V- 2 \lambda _R) f_R \Vert _2 \, |x-y|^\alpha \end{aligned}$$

With $\Vert V f_R \Vert _2 \le \Vert V \Vert _3 \Vert f_R \Vert _6 \le C \Vert f_R \Vert _{H^1} \le C (1 + \lambda _R ) \Vert f_R \Vert _2$, we conclude that $0 \le f_R (x) \le 1 + C \Vert f \Vert _2$ for all $|x| \le R_0$ (because we know that $f_R (x) \le 1$ for $R_0 \le |x| \le R$). To improve this bound, we go back to the differential equation (167), to estimate

196 $$\begin{aligned} \varDelta f_R = \frac{1}{2} V f_R - \lambda _R f_R \ge - \lambda _R (1+C \Vert f \Vert _2) \end{aligned}$$

This implies that $f_R (x) + \lambda _R (1+C \Vert f \Vert _2) x^2 /2$ is subharmonic. Using (192), we find $f_R (x) \le 1 - C \varepsilon _R^2$ for $|x| = R_0$. From the maximum principle, we obtain therefore that

197 $$\begin{aligned} f_R (x) \le 1 - C \varepsilon ^2_R + C \lambda _R (1+C \Vert f_R \Vert _2) \end{aligned}$$

for all $|x| \le R_0$. In particular, this implies that $\Vert f_R \mathbf{1}_{|x| \le R_0} \Vert _2 \le C + C \lambda _R \Vert f_R \Vert _2$, and therefore that

$$\begin{aligned} \Vert f_R \mathbf{1}_{R_0 \le |x| \le R} \Vert _2 \ge \Vert f_R \Vert _2 (1 - C \lambda _R) - C \end{aligned}$$

With $f_R (x) \le 1$ for $R_0 \le |x| \le R$, we find, on the other hand, that $\Vert f_R \mathbf{1}_{R_0 \le |x| \le R} \Vert _2 \le C R$. We conclude therefore that $\Vert f_R \Vert _2 \le CR$ and, from (197), that $f_R (x) \le 1 - C \varepsilon _R^2 + C/R \le 1$, for all $|x| \le R_0$, if *R* is large enough.

We are now ready to prove the lower bound for $\lambda _R$. We use now that any function $\varPhi$ satisfying Neumann boundary conditions at $|x|=R$ can be written as $\varPhi (x)=q(x)\varPsi _R(x)$, with $\varPsi _R(x)$ the trial function used for the upper bound and $q>0$ a function that satisfies Neumann boundary condition at $|x|=R$ as well. This is in particular true for the solution $f_R(x)$ of (167). In the following we write

$$\begin{aligned} f_R (x) = q_R (x) \varPsi _R(x) \end{aligned}$$

where $q_R$ satisfies Neumann boundary conditions at $|x|=R$. From (184), we find $|\varPsi _R (x)| \ge C / \log (ka)$. The bound $f_R(x)\le 1$ implies therefore that there exists $c>0$ such that

198 $$\begin{aligned} q_R(x) \le C \log (k{{\mathfrak {a}}}) \qquad \forall \, |x| \le R_0 \,. \end{aligned}$$

From the identity

$$\begin{aligned} {\mathfrak {h}} f_R= ({\mathfrak {h}} \varPsi _R) q_R - (\varDelta q_R) \varPsi _R - 2 \nabla q_R \nabla \varPsi _R \end{aligned}$$

we have

$$\begin{aligned} \int _{|x|\le R} \text {d}x \, f_R {\mathfrak {h}} f_R = \int _{|x|\le R} \text {d}x \, |\nabla q_R|^2 \varPsi _R^2 + \int _{|x|\le R} \text {d}x \, |q_R|^2 \varPsi _R {\mathfrak {h}} \varPsi _R\,. \end{aligned}$$

From (188) and (189), we have

$$\begin{aligned} \left| \varPsi _R (x) ({\mathfrak {h}} \varPsi _R) (x) - k^2 \varPsi ^2_R (x) \right| \le \, C \frac{k^2}{|\log k a |} (1+ V(x)) \chi (|x| \le R_0)\,. \end{aligned}$$

Hence

199 $$\begin{aligned} \int _{|x|\le R} \text {d}x\, f_R {\mathfrak {h}} f_R \ge \; k^2\Vert f_R\Vert ^2 - \frac{C k^2}{|\log k|} \int _{|x|\le R_0} \text {d}x \, (1+V(x)) |q_R (x)|^2 \,.\end{aligned}$$

With (198), we obtain

$$\begin{aligned} \int _{|x|\le R}\text {d}x\, f_R {\mathfrak {h}} f_R \ge k^2\Vert f_R\Vert ^2 - C k^2 \log (k{{\mathfrak {a}}})\,. \end{aligned}$$

With (195) (recalling that $R_*=\max \{R_0, e {{\mathfrak {a}}}\}$), we bound

$$\begin{aligned} \Vert f_R\Vert ^2 \ge \int _{R_* \le |x| \le R} |f_R (x)|^2 \, \text {d}x \ge \frac{C R^2 }{\log ^2(R/{{\mathfrak {a}}})} \end{aligned}$$

and, inserting in (199), we conclude that

$$\begin{aligned} \begin{aligned} \lambda _{R} = \frac{{\bigl \langle {f_R, {\mathfrak {h}} f_R}\bigr \rangle }}{{\bigl \langle {f_R,f_R}\bigr \rangle }}&\ge k^2 \left( 1 - \frac{C \log ^3(R/{{\mathfrak {a}}})}{R^2 } \right) \\ {}&\ge \frac{2}{ R^2 \log (R/{{\mathfrak {a}}})} \,\left( 1+\frac{3}{4}\frac{1}{\log (R/{{\mathfrak {a}}})} -\, \frac{C}{\log ^2 (R/{{\mathfrak {a}}})} \right) \,, \end{aligned}\end{aligned}$$

where in the last inequality we used (175).

To prove (169) we use the scattering equation (167) to write

$$\begin{aligned} \int \text {d}x\, V(x) f_{R}(x) = 2 \int _{|x| \le R} \text {d}x\, \varDelta f_{R}(x) + 2 \int _{|x| \le R} \text {d}x \lambda _{R} f_{R}(x)\,. \end{aligned}$$

Passing to polar coordinates, and using that $\varDelta f_R (x) = |x|^{-1} \partial _r |x| \partial _r f_R (x)$, we find that the first term vanishes. Hence

$$\begin{aligned} \int \text {d}x\, V(x) f_{R}(x)= 2 \lambda _R \int \text {d}x \, f_{R} (x) \,. \end{aligned}$$

With the upper bound $f_R(r) \le 1$ and with (168), we find

$$\begin{aligned} \int \text {d}x\, V(x) f_{R} (x) \le 2 \pi R^2 \lambda _R \le \frac{4 \pi }{\log (R/{{\mathfrak {a}}})} \left( 1 + \frac{C}{\log (R/{{\mathfrak {a}}})} \right) \,. \end{aligned}$$

To obtain a lower bound for the same integral we use that $f_{R}(r) \ge 0$ inside the range of the potential. Outside the range of *V*, we use (192). We find

$$\begin{aligned} \int \text {d}x\, V(x) f_{R}(x) \ge 4 \pi \lambda _R \int _{R_0}^R \text {d}r \, r \, (1- C \varepsilon _R^2 \log (R/r) ) \ge \frac{4 \pi }{\log (R/{{\mathfrak {a}}})} \left( 1 - \frac{C}{\log (R/{{\mathfrak {a}}})} \right) \end{aligned}$$

We conclude that

$$\begin{aligned}\left| \int \text {d}x\, V(x) f_{R}(x) - \frac{4\pi }{\log (R/{{\mathfrak {a}}})} \right| \le \frac{C}{\log ^2 (R/{{\mathfrak {a}}})}.\end{aligned}$$

Finally, we show the bounds in (170). For $r\in [R_0, R]$, from (192) we have

200 $$\begin{aligned} \left| \, w_R (x)- \frac{\log (R/|x|)}{\log (R/{{\mathfrak {a}}})}\,\right| \le \frac{C}{\log (R/{{\mathfrak {a}}})}\,. \end{aligned}$$

As for the derivative of $w_R$ we use (193) to compute

$$\begin{aligned} \left| \partial _r f_R (x)\right| \le \frac{C}{|x|} \frac{1}{\log (R/{{\mathfrak {a}}})}\,. \end{aligned}$$

Moreover $\partial _r f_R (x)= 0$ if $|x| = R$, by construction.

On the other hand, if $|x| \le R_0$, we have $w_R (x)= 1- f_R(x)\le 1$. As for the derivative, we define $\widetilde{f}_R$ on ${\mathbb {R}}_+$ through $\widetilde{f}_R (r) = f_R (x)$, if $|x| = r$, and we use the representation

$$\begin{aligned} \widetilde{f}'_R (r) = \frac{1}{r} \int _0^r \text {d}s \big ( \widetilde{f}''_R(s) s + \widetilde{f}'_R (s) \big )\,. \end{aligned}$$

With (167), we have (with $\widetilde{V}$ defined on ${\mathbb {R}}_+$ through $V(x) = \widetilde{V}(r)$, if $|x| = r$)

$$\begin{aligned} \widetilde{f}''_R (r) + \frac{1}{r} \widetilde{f}'_R (r) = \lambda _R \widetilde{f}_R (r) - \frac{1}{2} \widetilde{V} (r) \widetilde{f}_R (r)\,, \end{aligned}$$

By (200), we can estimate $\widetilde{f}_R (R_0) \le C / \log (R/{{\mathfrak {a}}})$. From (196), we also recall that

$$\begin{aligned} \widetilde{f}_R (r) \le \widetilde{f}_R (R_0) + C R \lambda _R \le C /\log (R/{{\mathfrak {a}}}) \end{aligned}$$

for any $r < R_0$. We conclude therefore that

$$\begin{aligned} \begin{aligned} |\widetilde{f}'_R(r) |&= \Big |\frac{1}{r} \int _0^r \text {d}s s \big ( \lambda _R \widetilde{f}_R(s) - \frac{1}{2} \widetilde{V} (s) \widetilde{f}_R(s)\big ) \Big | \\&\le \frac{\lambda _R}{r} \int _0^r r dr + \frac{C}{r \log (R/{{\mathfrak {a}}})} \int _0^r dr \, r \widetilde{V} (r) \\&\le \frac{C}{\log (R/{{\mathfrak {a}}})} + C\, \Vert V \Vert _2 \frac{\log (R_0/{{\mathfrak {a}}})}{\log (R/{{\mathfrak {a}}})} \le \frac{C}{\log (R/{{\mathfrak {a}}})} \,. \end{aligned} \end{aligned}$$

$$\square $$
